# Genome-Wide Effects of Long-Term Divergent Selection

**DOI:** 10.1371/journal.pgen.1001188

**Published:** 2010-11-04

**Authors:** Anna M. Johansson, Mats E. Pettersson, Paul B. Siegel, Örjan Carlborg

**Affiliations:** 1 Department of Animal Breeding and Genetics, Swedish University of Agricultural Sciences, Uppsala, Sweden; 2 Department of Animal and Poultry Sciences, Virginia Polytechnic Institute and State University, Blacksburg, Virginia, United States of America; 3 Department of Cell and Molecular Biology, Uppsala University, Uppsala, Sweden; University of Arizona, United States of America

## Abstract

To understand the genetic mechanisms leading to phenotypic differentiation, it is important to identify genomic regions under selection. We scanned the genome of two chicken lines from a single trait selection experiment, where 50 generations of selection have resulted in a 9-fold difference in body weight. Analyses of nearly 60,000 SNP markers showed that the effects of selection on the genome are dramatic. The lines were fixed for alternative alleles in more than 50 regions as a result of selection. Another 10 regions displayed strong evidence for ongoing differentiation during the last 10 generations. Many more regions across the genome showed large differences in allele frequency between the lines, indicating that the phenotypic evolution in the lines in 50 generations is the result of an exploitation of standing genetic variation at 100s of loci across the genome.

## Author Summary

Evolution is the process of change in response to selection. Typically, this results in more or less obvious changes to the appearance and physical properties—the phenotype—of an organism. However, these changes reflect underlying changes to the genome of that organism—the genotype. We examine the genomes of two lines of chickens that share a very recent ancestry but have been subjected to 50 generations of selection for high or low body weight, respectively. The effect of selection on the phenotype was dramatic, where on average high line birds are nine times heavier than low line ones. The effect on the genotype was equally dramatic, with a large number of changes distributed all across the genome. We observed more than 100 regions where different genetic variants were established in the two lines of chickens, which is considerable given the number of generations involved.

## Introduction

Evolution is the process by which populations adapt genetically in response to selection. Understanding the genetic mechanisms leading to phenotypic differentiation requires identification of the regions in a genome that are, or have been, under selection. Maynard Smith and Haigh [1] proposed to find these loci by searching for genetic hitch-hiking (now also called “selective-sweeps”[2]). Most reported selective-sweeps surround novel, major effect mutations that appeared on a single haplotype before sweeping through a population. A potentially more common type of sweep starts from standing genetic variation present at the onset of selection - the “soft sweep” [3]–[6]. Domestic animals and plants have been used as models to study both simple monogenic and complex polygenic traits. One of the unique features of these populations is that their reproduction has been under human control for a long time and planned selection of individuals have led to an exceptionally wide range of phenotypes within species.

Here, we report the results of a genome wide scan, using a 60 k SNP chip, in two chicken lines from a long-term, bi-directional, single trait selection experiment. In the Virginia chicken lines used in this study, 40 generations of selection resulted in a nine-fold difference in 56-day body weight (the selected trait) between the lines [7]. Long-term selection experiments, where animal and plant breeders have subjected populations to very strong and meticulously documented directional selection for generations, provide a valuable resource for studying the effects of selection [8], [9]. The resulting populations are examples of accelerated evolution, where the genetic and phenotypic changes that resulted correspond to changes that would most likely take centuries to achieve with the selection pressures in natural populations.

The Virginia lines are a chicken resource population for studying the genetic, genomic and phenotypic effects of long-term, single trait, divergent artificial selection [10]. In 1957, founders for one high- and one low- body weight line were selected from a 7-way cross between partially inbred White Plymouth Rock chickens. Once a year, with some restrictions imposed to minimise inbreeding, the birds with the highest and lowest 8-week body weight within each respective line were selected as parents for the next generation. After more than 40 generations of selection, there was a 9-fold difference in body weight between the lines [7] and a significant selection response continues through 50 generations of selection. Sublines, where selection was relaxed, were established periodically within both the high and low body weight lines to serve as unselected controls. After some generations, the relaxed lines originating from the high line had lower body weights than the line continuously selected for high body weight, and the relaxed lines originating from the low line had heavier body weights than the selected low line [10]. This pattern reinforces the notion that the observed change in phenotype is indeed due to the continuous selection process. The Virginia lines are a valuable resource for studying the effects of selection on the genome. Of particular importance is that the experiment involved bi-directional selection and that the population history, including population sizes, selection intensities as well as expected and observed selection responses each generation are known. This information allows a better separation of the genomic effects of selection and drift than would otherwise be the case. Together with the advent of a new high-density chicken SNP chip the Virginia lines allows a detailed investigation of the effect of selection on the genome that was not previously possible.

One current paradigm for identifying selective sweeps (hitch-hiking) is to scan the genome of a selected population for regions of homozygosity (e.g. Sabeti and co-workers [11]). In these analyses, it is assumed that the selected allele was present on a single haplotype at the beginning of selection, which is the case when selection acts on a novel mutation. When the beneficial allele is present on multiple haplotypes, effects of selection will not be detected using this approach. If there is standing (or cryptic) genetic variation in a population, which is likely when selecting on mutations that have existed in a population for some time before the onset of selection, the expected pattern of fixation is different [3]–[6], [12]. Although little is known on how common it is that selection starts from standing variation, initial studies with soft sweeps based on limited marker sets and partial genome coverage [13], [14] indicate that they might be common. In the Virginia lines, selection started from a mixed population where, at each selected locus, the selected allele might be present on haplotypes from any of the founder lines of the base-population. The selected allele might thus be in high linkage disequilibrium, LD, with some marker alleles (i. e. SNPs) and lower LD with other marker alleles that are physically close on a chromosome. Therefore we would not necessarily expect to observe regions with complete fixation of all SNPs around the selected loci, but instead regions where some SNPs display large frequency differences between lines (in the extreme case fixed for different alleles) and other adjacent SNPs with little frequency differences between lines.

Because evidence for selection is strong in these lines, as shown by the selection response and results from the relaxed lines, our aim was to identify the genetic elements that are the most likely to have been under intense selection by identifying the regions in the genome with the most extreme allele frequency differences between the lines.

Here, we report on a genome-wide scan for soft-sweeps designed to identify those SNPs that are in LD with regions in the chicken genome that have been under selection during the breeding of the Virginia lines. Analysing 57,636 SNPs in individuals from both the high- and low body weight lines after 40 and 50 generations of selection provides a detailed analysis of both past and present genomic effects of selection as well as insights into how selection has acted on the genome in order to achieve the considerable response to selection.

## Results

### Fixations in the two lines

**Figure 1:**
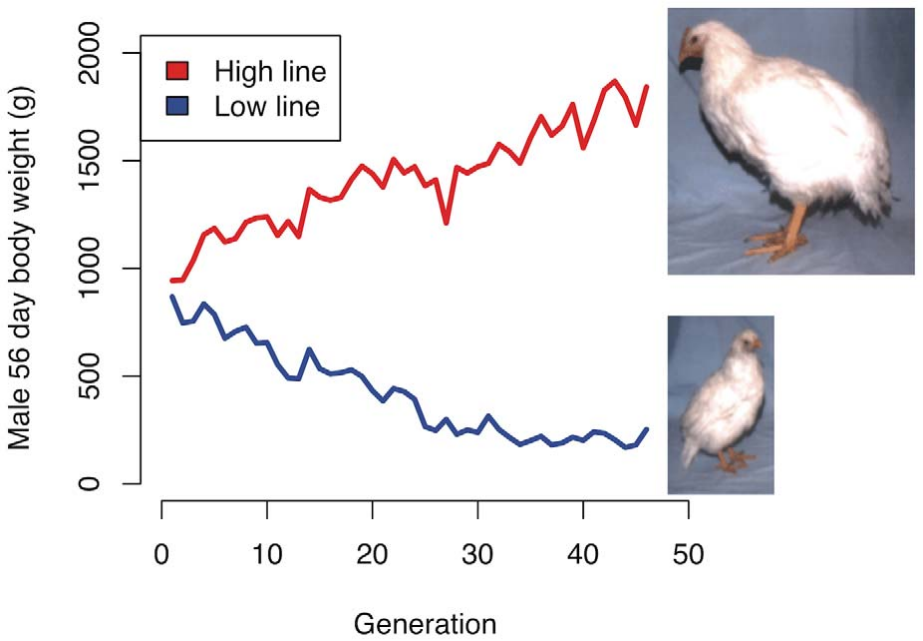
Selection response in the Virginia chicken lines. The red and blue lines show the average male body weight in the high and low body weight lines, respectively. The high line is still responding to selection, whereas the low line appears to have phenotypically plateaued. The photos are from generation 37.

Genotypes from both the high and the low lines were studied at two time-points, namely after 40 and 50 generations of selection. 57,636 SNPs were genotyped in 20 individuals from each line after 40 generations of selection and in 10 individuals from the low line and 49 from the high line after 50 generations of selection. The 60 K SNP chip provides a marker density of approximately 1 marker/15 kb. The extent of LD for the SNPs on this chip in the population is not known, but estimates from genome re-sequencing of the lines suggests an LD block size in these populations of 30 kb (micro chromosomes) - 60 kb (macro chromosomes). The extent of LD is expected to be relatively large due to three relatively recent bottle-necks in these populations from breed-formation, inbreeding of lines used to create the base population as well as limited size of the base-population. It is, however, unlikely that any of the SNPs on the chip is causative, but most causative mutations are likely to be linked with at least one marker. 56,586 SNPs had genotypes in both lines after 40 generations and 56,561 after 50 generations of selection. Of the 32,846 SNPs that were polymorphic in generation 40, 13,579 were polymorphic in both lines, 10,237 only in the low line, 8,032 only in the high line and 998 were fixed for alternative alleles in the two lines. There were more fixed SNPs in the sample from the high line, which was expected based on the empirical observation that the phenotypic response to selection ceased in the low-line about generation 30 (Figure 1). In generation 50, an additional 748 SNPs were fixed for different alleles in the two lines – an increase by 75% – most of which were already fixed in one line at generation 40 (Tables S1 and S2).

### Allele frequency differences between lines and generations

**Figure 2:**
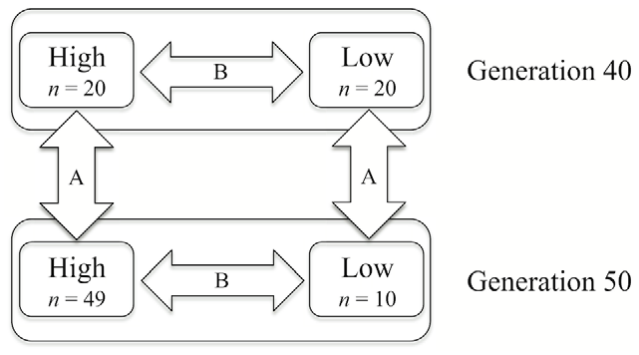
Schematic view of the groups of genotyped individuals and outline of the comparisons made in this study. The number of genotyped birds in each group is shown as *n*. The arrows represent the different comparisons made; A is across time but within the same line, B is between lines within generation.

Figure 2 illustrates the different samples included in the study and the two types of comparisons made using these data. First, allele frequencies at all SNPs were compared across time within each line (arrows labelled A in Figure 2). This comparison identifies the regions within each line with the largest changes in allele frequencies between generations 40 and 50. Then, allele frequencies for all SNPs were compared between the high and low lines at two different time points: generations 40 and 50 (arrows labelled B in Figure 2) to identify where in the genome the SNPs indicate the strongest divergence between the lines. To evaluate the significance of observed differences in allele frequencies between lines and sample points within a line, association analyses using PLINK [15] were performed.

**Figure 3:**
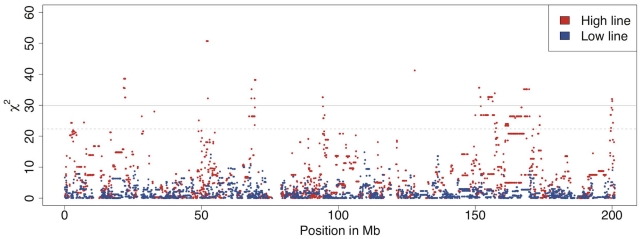
Allele frequency changes between generation 40 and 50 across chromosome 1 as measured by association analysis. There are several regions on chromosome 1 with significant allele frequency differences between generation 40 and 50 for in the high line (red) but not in the low line (blue). The solid grey line indicates the Bonferroni corrected significance level p<0.001 and the dashed grey line the corrected p<0.05.

Within line comparisons of frequencies at generation 40 and 50 (comparisons A in Figure 2) are performed to reveal the effects of recent and ongoing selection. The analyses identified significant differences in many regions dispersed over the entire genome. In the high line, there are highly significant changes in allele frequencies (p<0.001) on 10 chromosomes and significant changes (p<0.05) on 6 additional chromosomes. For example on chromosome 1 (Figure 3) there were six regions with significant differences between generations 40 and 50 in the high line and those regions are thus the most likely to have been under intense recent selection within this line. The low line only shows significant differences (p<0.05) on two chromosomes (for details see ). This lower number of currently affected regions is expected given the low response to selection since about generation 30.

**Figure 4:**
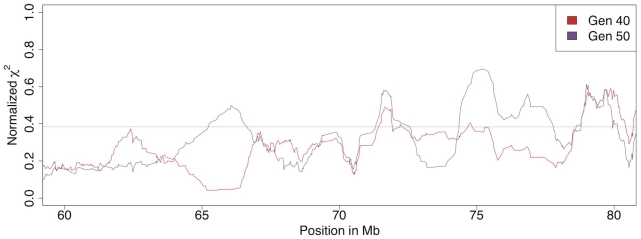
Allele frequency differences between the high and low body weight selected lines. An association test for comparison *B* in Figure 2 shows a big difference in how quickly allele frequencies change between generation 40 and 50 in the high- and low lines. In the region between 60 and 80 Mb on chromosome 4 there are large differences between the results from generations 40 and 50. The sliding window mean of 20 markers is shown as a red line for generation 40 and as a purple line for generation 50. The scale is normalized for easier comparison between the time points because their maximum χ^2^ is different (80 at generation 40 and 118 at generation 50).

Comparisons between the high and low lines at generations 40 and 50 (comparisons B in Figure 2) revealed many highly significant differences between them across the genome at both time points (Figure S2). For example, there were at least ten regions with highly significant allele frequency differences between the lines on chromosome 4 both at generation 40 and 50. These regions were likely to have been under intense selection earlier in the selection process. An example of a region with recent divergence between the lines was between 60 Mb and 80 Mb on chromosome 4 (Figure 4). This could be an interesting region to study further as the different selection response in the lines could be caused by the region containing one or several genes that display genetic background dependent effects (i.e. epistasis). It is noteworthy that despite the relatively low number of individuals, a test for allele frequency differences yields a χ^2^ value of 80 for a SNP fixed for different alleles in the two lines, which is highly significant even with full Bonferroni correction for multiple testing. For comparisons with other studies it is also useful to realize that χ^2^ and p values from the allelic χ^2^-test is the same as a χ^2^-test of Fst, i.e Fst was also highly significant at all the identified regions across the entire genome.

To measure the dynamics within the genomes of the low and high lines, allele frequency changes resulting from 10 generations of selection (from generation 40 to 50) were studied. The loci with the highest rates of allele frequency changes are the most likely regions to contain genes under current selection.

**Figure 5:**
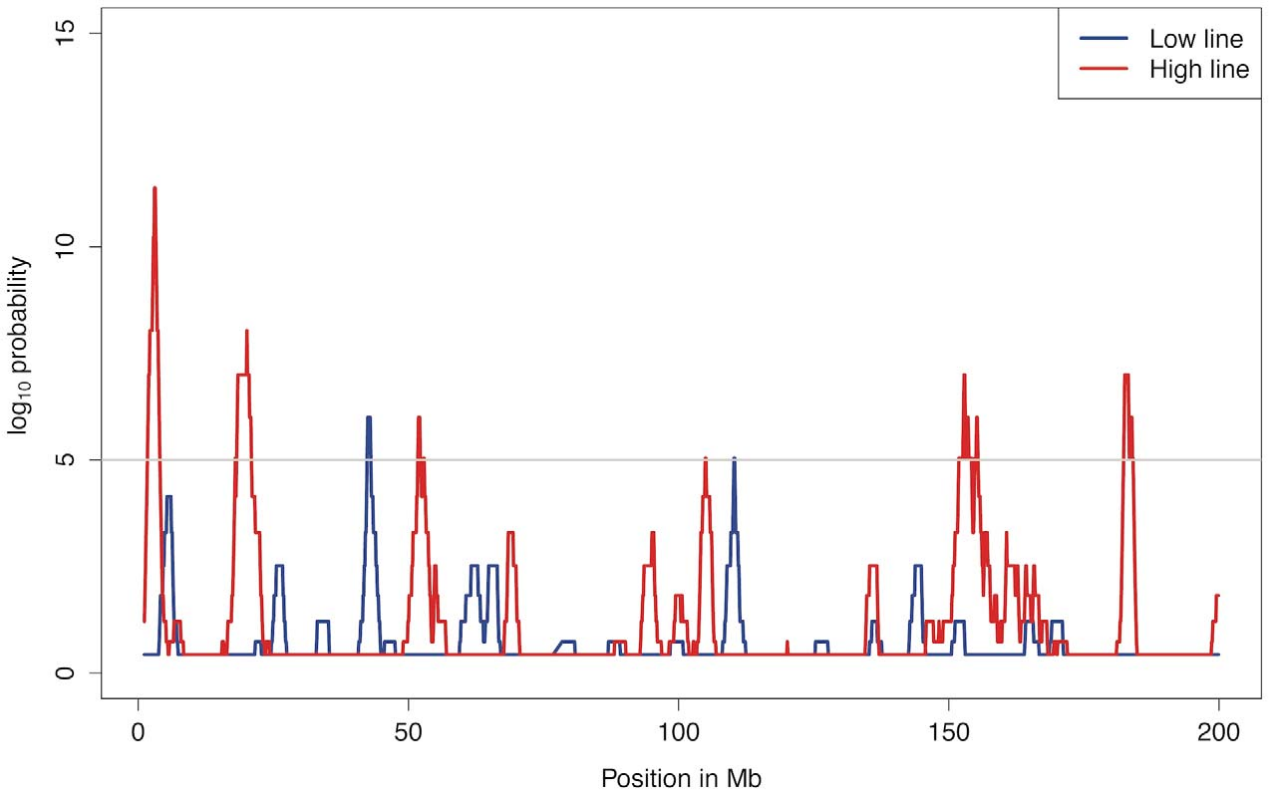
Significance for allele frequency changes on chromosome 1. Average allele frequency changes (comparison A in Figure 2) over blocks of 5 markers were calculated and the blue and red lines indicate the number of loci with unexpectedly high changes in a window of 20 blocks. The light grey line corresponds to a 95% confidence genome wide (p = 10^−5^).

In total, there are 24 regions with significant allele frequency changes in at least one line, spread across the genome. Only one region, the beginning of chromosome 7, was significantly affected in both lines. This lack of correspondence is not entirely unexpected because the lines have undergone a large number of independent fixation events, which makes it unlikely that the same regions are concurrently under selection after 40 generations of divergent selection. Figure 5 shows the results for chromosome 1. The complete results for all chromosomes are provided in Figure S3.

### Simulations

A complicating factor when attempting to identify regions under selection, especially with small effective population sizes, is to discriminate between the effects of selection and drift. Because the full population history of these lines is known, we could use simulations to evaluate how selection and drift were expected to affect the genome. Previous studies to identify QTLs [7], [16], [17] indicate that selection has been strong on many loci in the genome. Using the estimated effects of the QTLs to calculate the selection coefficient (*s*) [18], [19], yields values of s in the range 0.19–0.93 (Table S3). The simulations show that selection on these loci was sufficiently strong to lead to high probability of fixation after only 10–15 generations for the loci with larger effects and well before generation 40 for many other loci (Table S4 and S5). After 40 generations, the loci with the largest selection coefficients (i.e. those representing the effects of significant QTL for the selected trait) always reaches fixation for the selected allele during the simulations with additive alleles. This is illustrated in Figure S4A, S4B, S4C, where selection is applied on the loci *Growth4* (selection coefficient for males, *s_M_* = 0.56, and selection coefficient for females, *s_F_* = 0.34), *Growth6* (*s_M_* = 0.93, *s_F_* = 0.56) and *Growth9* (*s_M_* = 0.79, *s_F_* = 0.48) in the high line. Even for the QTL with the smallest effect, *Growth12* (*s_M_* = 0.31, *s_F_* = 0.19), fixation occurred in 85% of the replicates at generation 40 (Figure S4D). Using a selection coefficient half the size of the smallest QTL (i.e *s_M_* = 0.15, *s_F_* = 0.10) and otherwise the same parameters, gives fixation in 45% of the replicates. Keep in mind that these values are for fixation within a single line, they should be squared to obtain the probability of concurrent fixation in both lines.

The effective population size, *N_e_*, for the selected lines estimated from the number of parents each generation is ∼35 (See Table S6 for details). Calculations of *N_e_* from the actual pedigrees up until generation 48 show higher values (44.5 for the high line and 49.3 for the low line) [20]. This demonstrates that the breeding scheme to limit inbreeding has been successful. Using *Ne* = 35, the *N_e_s* for the previously identified QTL with the smallest effect is, 35×0.19 = 6.6, which is greater than 1 implicating that selection is the predominant force at this locus [21]. The simulations support this, as the selected allele is always the one that becomes fixed even for the QTL with the smallest effect. It should, however, be noted that the simulations use effects estimated for statistically significant QTL for the selected trait in a line-cross experiment. As these might include multiple genes affecting the trait and there will be a large number of additional loci with smaller effect on the trait, there will also be a large number of loci for which a balance between selection of drift will have determined which allele has been fixed at the end of the experiment. Our results do, however, show that the population size has been sufficiently large to prevent genetic drift from overriding the effect of selection for the loci with the largest *s*-values in the selected lines. The simulations also show that for a locus with no selection (i.e. where there is only genetic drift), fixation at this locus in one of the lines only occurs in 10–20% of the replicates when the allele frequencies are intermediate in the base population (3/7 and 4/7) and in approximately 50% of the replicates when the initial frequencies are more uneven (1/7 and 6/7) (Figure S5). The probability of observing fixation of one of the alleles in one line or the same allele in both lines is thus rather high, which is what we observe in the data. Approximately 30% of the SNPs were fixed in one line and not in the other, while at another 45%, they were fixed for the same allele. It should, however, be noted that the group of markers displaying fixation for the same allele in both lines contain both those SNPs that have drifted to fixation and those that were monomorphic in the common base-population. The simulations showed that the probability of fixation of one allele in one line and the other allele in the other line by drift is very low. If the initial allele frequencies in the base-generation are 3/7 and 4/7 (the base population is a mixture of 7 lines) the probability of fixation of different alleles is: 2 * (fixation probability for A) * (fixation probability for a))  = 2*0.038*0.094 = 0.0072≈0.7%, for 2/7 and 5/7 it is 0.4% and for A = 1/7 and a = 6/7 it is 0.2%. The corresponding numbers for fixation of the same allele are 1%, 6% and 27%, respectively. If we assume a uniform distribution of initial frequencies, the expected proportion of loci fixed for the same allele in the two lines would be 11% and the proportion fixed for different alleles in the two lines 0,44%. Since an unknown, but likely substantial, fraction of the SNPs were fixed in the base population, this value cannot easily be compared to the observed data. However, we can compare the observed fixation rate between generation 40 and 50 with the corresponding value from the simulations. In the simulations, the ratio of fixation of the same allele divided by fixation of different alleles is 3.98, again assuming a uniform allele frequency (an assumption that closely matches the true distribution of segregating SNPs in the data [data not shown]), whereas the observed ratio is 2.12. This indicates that about 50% of the fixations for different alleles are due to selection rather than drift. Given the decreased selection response in the low line during this period, it is likely that this figure is lower than the average for the entire selection process.

We can also look at the raw number of expected fixations of different alleles to estimate the proportion of SNPs fixed by drift. In the worst-case scenario, where all 56,000 SNPs would have segregated at intermediate frequencies (we used 3/7 and 4/7 as the founder population was a mixture of 7 partially inbred lines) in the original population, at least 60% of the observed fixations for different alleles at 40 generations would be due to selection. If instead we assume a uniform distribution of allele-frequencies in the base population, the proportion of the markers fixed for alternative alleles due to selection would be 70%.

These two alternative ways of separating the effects of drift to selection are in reasonably good agreement, and indicate that the proportion of fixed SNPs due to selection is in the range of 50% to 70%.

### Heterozygosity in the two lines

The observed mean heterozygosity, *H_o_*, was calculated at all autosomal loci in each line at both time points. *H_o_* at 40 generations was 0.146 and 0.156 in the high and low lines, respectively. After 50 generations, *H_o_* had decreased to 0.130 and 0.142. This decrease in heterozygosity was significantly (p = 0.0003) larger in the high line, and because the population structure is the same in both lines, it is logical that this excess is primarily a function of selection. We also observed a greater loss of genetic variance in the high line during the last generations of selection when the response had weakened in the low line. All this is consistent with the greater response to selection in the high line during those ten generations of the selection experiment. Selection, however, continues in the low line and thus the difference in heterozygosity loss only provides a minimal estimate of the effect of selection.

### Expected number of loci determining the trait

Several theoretical methods exist for estimating the number of genetic factors (loci) that determine a complex trait in an experimental intercross between divergent lines [22], [23], [24], [25]. The procedure of Otto and Jones [25], which takes information about the difference in mean between the parental lines and the effects of known QTL as input to predict the distribution of remaining additive effects, was used to estimate the number of loci affecting body weight in the intercross. When employing the most recent estimates of QTL effects in the lines [17], this method predicted that the selected trait - body weight at 56 days of age - was determined by 121 loci (Table 1). This is consistent with our result from comparison on allele frequencies between the two lines, indicating that the selected trait is determined by a large number of loci. These estimates are, however, only an indication of the true number of data. But it is interesting to note that all data indicate that the number of loci involved is more likely to be large (in the order of 100s) rather than small.

**Table 1:** Quantitative genetic estimations of number of loci determining body weight in the Virginia lines.

Trait	M	a_min_	n_d_	T	n_QTL_
BW14	2.9	2.4	3	2.2	958
BW28	9.6	8.2	3	7.52	327
BW42	21.3	18.3	3	16.8	151
BW56	37.9	34.2	3	32.4	121
BW70	54.7	51.4	3	49.8	137
GR0–14	2.9	2.4	3	2.1	838
GR42–56	16.2	14.5	3	13.7	268
GR56–70	17.8	12.2	3	9.4	79

### Number of loci under concurrent selection

The genome-wide QTL profile from the scan for loci affecting body weight at 56 days of age in an F_2_ intercross between the selected lines [7] reveals about 30 discrete peaks, where there is a significant (nominal p<0.05) additive genetic effect. We expect the distribution of the estimated genetic effects of these loci, even though they do not reach the experiment-wide significance threshold, to have a distribution that resembles that of the genetic effects of the true loci that determine the line difference. The observed distribution is approximately exponential (Figure S6), and as a consequence of this, the relative differences in genetic effects between the ordered loci are more or less constant. The *s*-values for the loci are not dependent on the absolute size of the genetic effects - they are determined by the distribution of the genetic effects for the segregating loci, where in the ordered distribution the locus is and how many loci contribute to the trait. When the distribution of genetic effects is exponential, there is a gradient in the strength of selection on individual loci. The locus with the largest effect will be under more intense selection than the second largest locus and the difference in selection intensity is proportional to the relative difference in their genetic effects. Thus, even though all loci that affect the selected trait will technically be under selection at all times, there will always be a subset of loci under more pronounced selection in the population. In our simulations we show that the loci with the largest effects reach fixation in approximately 10–15 generations in this population. Fixation of these loci will affect the s-values for other loci via, at least, two mechanisms. Firstly, fixation of the strongest loci will increase the relative importance of all other loci. This is because (for additive genes) the selection differential scales with the allelic effect in standard deviations. As major genes are fixed, the genetic variance decreases and, as a consequence, so does the standard deviation, which results in an increase of the strength of selection. In the selection experiment, the standard deviations for 8 week weights for males from generations 20, 40 and 50 were 111, 139, and 179 g. The increase in standard deviation makes sense as we are seeing large phenotypic changes. Decreasing coefficients of variation do, however, indicate a decrease in the genetic variance due to selection. Respective values for the LW line, where there is a plateau at the phenotypic level were 63, 54, and 60 g. The changes in the relative strength of selection for the loci will depend on how their allelic effects scale – will weight increase with a constant amount over time or scale with increasing mean body weights in the population. This is not known, and cannot be estimated, but it is reasonable to expect a scaling with the mean and if so the relative strength of selection will increase over time for these loci. Secondly, earlier studies have shown that extensive capacitating epistasis in important in this population [16, Besnier, Pettersson and Carlborg, in preparation]. Due to genetic interactions, the genetic effects of some loci will increase with the changes in genetic background due to selection. In addition, new mutations that occur during the selection process might create entirely new selected alleles with larger selective advantage. In either case, it is unlikely that the current selection profile across the genome is different from what it was at onset of selection. When studying the effect of 10 generations of selection (from S40 to S50), we observe strong sweep signals in approximately 10 loci, which seems reasonable given the expected distribution of genetic effects.

### Clusters of fixation

**Figure 6:**
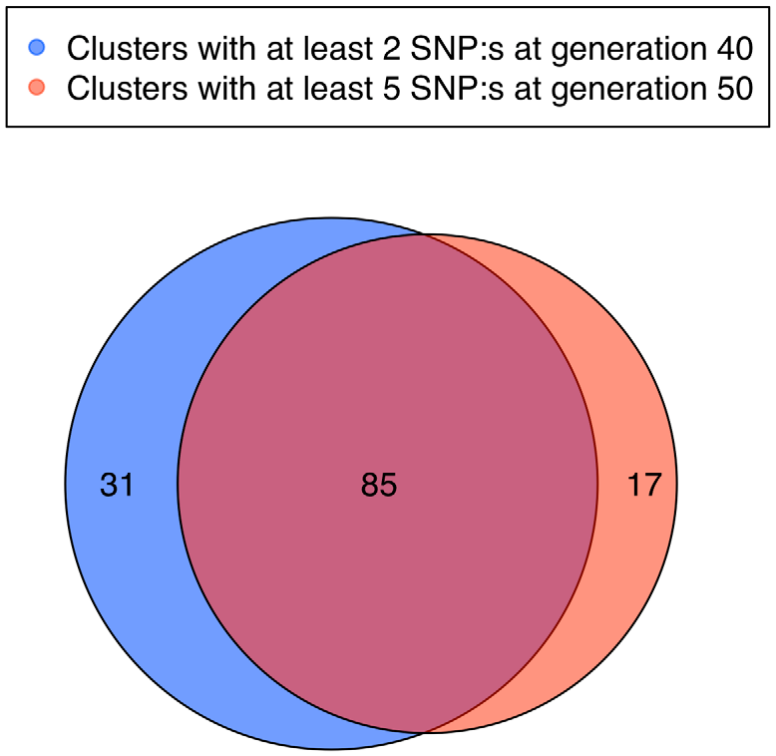
Clusters of SNPs fixed for different alleles in the two lines. Comparisons of clusters consisting of at least 2 SNPs in generation 40 (reddish pink) and at least 5 SNPs in generation 50 (blue). The overlap is considerable, as some clusters already contained more than 5 SNPs at generation 40 and others that with 2 SNPs in generation 40 were extended over those last 10 generations to include at least 5 SNPs in generation 50. Interestingly, several novel sets have appeared, indicating possible sites for ongoing selection. The sets that have not increased are unlikely to be under selection at this time in the selection process.

Using a clustering criterion that required a maximum of 1 Mb between subsequent fixed SNPs, there were 116 clusters of at least two SNPs that included 96.1% of the 998 SNPs fixed for different alleles and covered 10.2% of the genome. This indicates highly non-random spatial distribution of fixed SNPs, which is not what we expect to observe when drift is responsible for a majority of the fixations. Using a more stringent criterion of at least 5 SNPs per cluster, there were 65 clusters including 82.3% of the SNPs and covering 8.6% of the genome (Figure 6). In generation 50, there were 1746 SNPs fixed for different alleles in 163 clusters of at least 2 SNPs or, using the more stringent criterion, 102 clusters with at least 5 SNPs. The number of clusters and proportion of the genome covered is relatively stable to variation in the required number of SNPs in clusters and distance between markers (Table S7). Both in generations 40 and 50, more than half of the clusters with at least 5 SNPs were longer than 1 Mb and about a quarter was larger than 2 Mb (Table S8). The results for clusters with at least 2 SNPs are shown in Table S9. The size in Mb and cM of the 23 clusters longer than 2 Mb at generation 50 can be seen in Table 2. The largest physical cluster was 5.4 Mb long and located on chromosome 2. The largest cluster with respect to recombination distance was 23.3 cM and located on chromosome 24. Nine of the largest clusters overlapped with previously identified QTLs.

**Table 2:** Length in Mb and cM for the 23 selective sweeps that are longer than 2 Mb in generation 50.

Chr.	No of SNPs	Length (Mb)	Length (cM)	Overlapping QTL
1	12	2.01	5.04	Breast muscle, Insulin
1	17	3.53	8.81	
1	15	2.18	5.46	
2	35	5.42	11.93	
2	31	4.23	9.31	*Growth3*, Triglycerides
2	29	3.16	6.94	
4	28	3.53	8.12	*Growth6*, *Growth7*
4	15	2.31	5.30	*Growth6*, *Growth7*
4	23	4.48	10.31	*Growth6*, *Growth7*
4	8	3.54	8.15	*Growth6*, *Growth7*
4	38	4.26	9.80	
4	21	2.33	5.36	Breast muscle
5	46	2.80	7.27	*Growth8*
6	56	4.98	11.96	
6	21	2.73	6.56	
7	26	2.14	7.06	*Growth9*
10	29	2.97	12.20	
13	53	2.53	10.64	
15	68	3.63	19.26	
19	16	2.05	11.07	
24	28	2.54	23.32	
Z	23	3.86		
Z	30	3.40		

The cM/Mb ratio used is from Table 2 in [17] and the overlapping QTL from Table 2 in [7], Fle 3 in [17], and Table 5 in [24].

Depending on the criteria used for clustering, we thus observe between 102 and 163 clusters fixed for alternative alleles in the two lines at generation 50. Irrespective of the criteria used, these clusters contain more than 85% of the SNPs fixed for alternative alleles in the lines. Based on the calculations above, we expect that between 50–70% of the SNPs that are fixed for alternative alleles to be due to selection. If we conservatively assume that the fixed SNPs are distributed randomly inside and outside of clusters, we would then expect between 51 and 114 of the observed clusters to be fixed due to selection, This observation fits well with the expectation of 121 major factors contributing to selection response based on the quantitative genetic theory presented above.

As can be seen in Table 2, the size of the 23 largest clusters, in terms of recombination distances, ranges between 5.0 and 23.3 cM. Since the probability of recombination occurring in a given region increases exponentially with each generation, these regions were most likely fixed rapidly. As expected from population genetics theory (see e.g. [21]), our simulations show that fixation in a single line for a neutral locus takes considerably longer time than for a locus with s-values similar to those in our data. E.g. in 1000 simulated replicates, the first fixation for a neutral locus occurred after 12 generations and it took 35 generation before fixation was reached in 10% of the replicates. This should be compared with the 4 generations it took to reach the first fixation and the 9 generations it took for 10% to be fixed for the locus with the largest effect (Table 3). The probability that a region of 5 cM will remain un-altered by recombination during the sweep to fixation in this population is 0.078 in 3 generations, 0.014 in 5 generations and 2.1*10^−4^ in 10 generations for allele frequencies of 1/7 and 6/7 and 6.0*10^−3^ in 3 generations, 2.0*10^−4^ in 5 generations and 3.8*10^−8^ in 10 generations for allele frequencies of 3/7 and 4/7. This example illustrates how rapidly the probability of un-altered haplotypes decreases with increasing number of generations to fixation. Our results indicate that it is not that probable that 8 regions larger than 10 cM and an additional 10 regions 5–10 cM would have swept through the selected population in the time required for neutral loci to become fixed, and that selection is a more likely explanation for the fixation of these large clusters.

**Table 3:** Estimated fixation times (in generations).

QTL	First fixation (gen)	10% fixation (gen)	*s* (M/F)
*Growth4*	7	11	0.56/0.34
*Growth6*	5	8	0.93/0.56
*Growth9*	4	9	0.79/0.48
*Growth12*	9	16	0.31/0.19
Neutral	12	35	0/0

Times for fixation within a single selected line for QTL with *s* values (male/female) estimated from an F_2_ intercross between the selected lines and a neutral reference (*s* = 0). Estimates are given for when the first and 100th of the 1000 simulated populations are fixed.

Of the 116 clusters identified after 40 generations of selection, 63% contained at least two consecutive fixed SNPs and could therefore be considered as traditional hard sweeps. However, almost two thirds of them had only two consecutive fixed SNPs, and would not be detected under more stringent clustering criteria. The largest stretch of consecutive markers fixed for different alleles is located on chromosome 2 and contains 8 SNPs.

**Figure 7:**
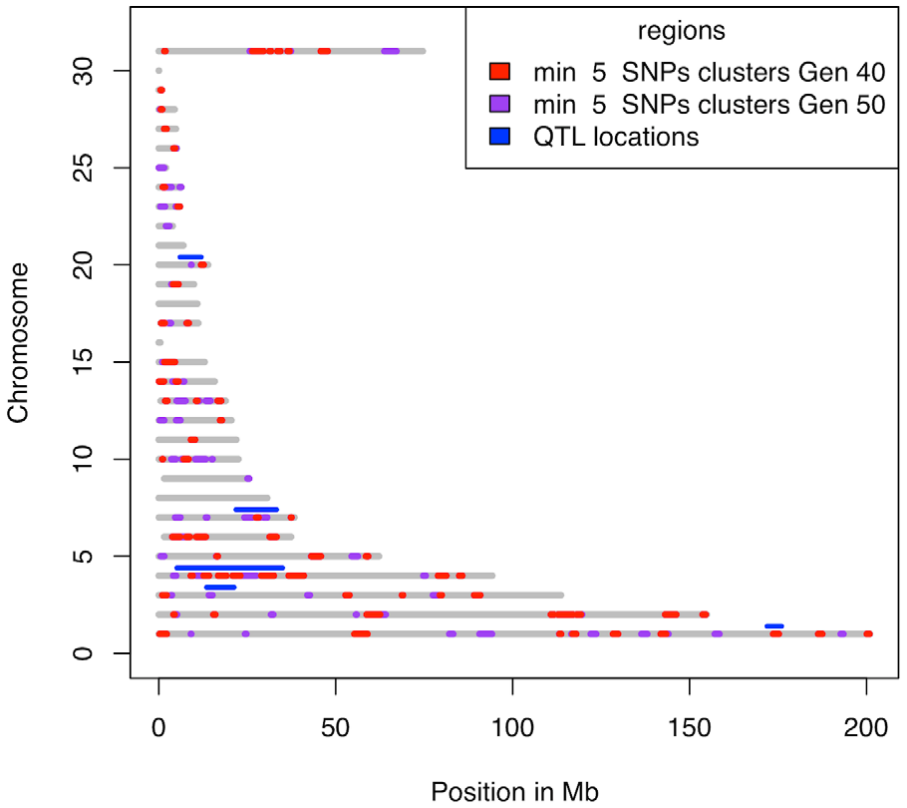
Clusters with at least 5 SNPs fixed for different alleles in the two lines. The grey lines represent the regions on the chromosomes covered by markers. Chromosome Z is numbered 31 and the linkage groups LGE22C19W28_E50C23 and LGE64 are numbered 29 and 30, respectively. Clusters at generation 40 are shown in red with the additional regions covered by clusters at generation 50 shown in purple. The maximum distance between subsequent fixed SNPs is 1 Mb. The significant QTL regions reported in [17] are shown in blue. As can be seen, all QTL regions contain one or several clusters.

In generation 50 those clusters with at least 5 SNPs overlapped to a large extent with clusters that contained at least 2 SNPs in generation 40. There were, however, 17 new clusters (Figure 6), which indicate that there were responses to selection at new loci during the last ten generations. Even though some of these new clusters might be due to drift, a number of them are likely to contain genetic elements that have recently come under effective selection. These could be alleles present already at the beginning, but which were not strongly selected due to a relatively small effect size compared to other loci, that have become more important as the scaled phenotypic variance decreases in response to selection [10] or they could be epistatic loci, the effect of which have increased due to changed genetic background [16]. Some of the loci may also be new favourable mutations, although the present data does not allow us to estimate how frequent these are. Moreover, all significant QTLs identified in the Virginia lines by Wahlberg et al. [17] contained one or several clusters of fixed SNPs (Figure 7).

## Discussion

Improving our understanding of the dynamic changes in allele frequencies that occur across the genome in response to selection is a challenge in genetics. The selective coefficients of loci will not remain constant throughout the time span of a long-term selection experiment. Loci with the largest effects are most likely to be fixed rapidly, resulting in an increase in the proportion of the total variance contributed by loci with smaller effects. Very little, however, is known about how many loci contribute to a complex trait and how many loci are under most intense selection, i.e. undergoing the most rapid allele-frequency changes, at a given point in time. Several recent studies indicate that the number of loci contributing to complex traits is considerable (Maize [26], Illinois corn selection lines [27], height in humans [28]). These insights were, however, gained from studies of the association between phenotypes and genotypes, which implicitly means that there will be limits on the power to detect loci due to sample size. Population history and selection for multiple traits also complicates the picture. Here, we study the genomic effects of intense selection on a single complex trait, which facilitates more precise insights on basic genetic regulation and dynamic changes that occur during selection.

Earlier genetic studies of the Virginia lines have shown that more than 20 genome regions (QTL) are involved in the genetic regulation of the trait under selection, body weight [7], [16], [17], as well as correlated responses including body composition and metabolic traits [29]. Our estimates of the expected number of loci contributing to the trait indicate that there are many loci that remain unidentified. The probability of fixation for alleles with small effects is higher when selection acts on standing genetic variation than on a new mutation, due to the high likelihood of losing a weakly selected new mutation from the gene-pool in the population. Thus, we would expect our approach to identify a larger number of loci than previous QTL mapping experiments that were based on these data because only loci with rather large genetic effect would have reached the detection threshold in those experiments. This is also what we observed. Both the quantitative genetic and molecular assays used to estimate the number of selected genetic elements are in agreement that we have evidence for there being from 50 up to over 100 regions in the genome that have been under strong selection over the first 50 generations of the selection experiment. This study demonstrates that selection on a complex trait will influence more regions than can be identified even in a comprehensive genetic mapping study, and that the genetic regulation of these traits is complex. Our criterion to require fixation for alternative alleles was very stringent and therefore it is likely that additional regions than those reported were actually under selection. This becomes apparent when examining data from generation 50, where 1776 SNPs were fixed for alternative alleles in our samples, including 17 new clusters of at least 5 fixed SNPs that were formed during the 10 last generations of the selection experiment. Some of these new clusters may have been selected already earlier but not strongly enough to reach fixation before 40 generations, while some might be due to new mutations that have occurred recently.

The footprints of selection include regions spread throughout the genome, including previously identified QTLs as well as those hitherto not implicated to affect body weight in chickens. As regions of fixation, of which many certainly contain selected regions, are identified with very high resolution (in many cases the clusters cover <1 Mb), this information can be useful for identifying candidate genes and mutations involved in the phenotypic response to selection. Assigning the functional effects to the identified regions, however, remains a future challenge.

Selection coefficients for the genomic regions (QTL) identified in previous studies of these lines ranged from 0.93 to 0.31 and 0.56 to 0.19 for high line males and females, respectively, with very similar values for the low line (Table S3). Even if some of these selection coefficients are overestimates, they are, as a group, very high and illustrate the massive selective pressure on the genome in these lines. The intensity of selection is the most likely explanation for the remarkable differences in allele frequencies observed across the whole genome.

Selective sweep analyses are powerful in identifying loci that display directional changes in allele frequencies that correlate with the phenotypic responses to selection. With the advent of more affordable methods for high-density genotyping and genome re-sequencing, it is a cost effective approach to identify loci determining complex traits because small samples from existing, divergent populations can be used [30]. The resolution often allows identification of individual genes and thus provides useful insights to the genes and plausible mechanisms involved in the regulation of the traits for which studied populations differ. A major drawback with the sweep analyses is, however, that they do not provide causal evidence for the involvement of particular genetic polymorphisms in phenotypic expression. The divergent populations studied often differ for multiple traits and it is not possible to identify which of these traits that is affected by the polymorphisms. Furthermore, there are no additional insights to the potential genetic mechanisms involved, i.e. whether genes act independently or through interactions in complex gene-networks. This information is, however, provided in e.g. linkage or association studies. Therefore it is necessary to realise that the selective sweep analyses are not a stand-alone method, but rather an addition to the complete set of tools used for understanding the inheritance of complex traits. An example of how sweep and linkage analyses complement each other is obvious in this population. We have earlier used linkage analysis to identify a network of loci that through strong interactions have a major influence on body weight at 56 days of age [16]. Subsequently we replicated the effects and refined their location in an independent advanced intercross line population (Besnier et al, in preparation). The epistatic network contains four loci on chromosomes 3, 4, 7 and 20 and there is a clear overlap between one or several sweeps in each of these regions with the QTL (Figure 7). Combining this information will be a highly useful strategy for identifying the causal mutations underlying the observed genetic interactions.

To conclusively rule out drift as the cause of any given fixation event or other observed change in allele frequencies is not possible. However, all available results indicate that the large phenotypic difference in body weight between the Virginia lines is the result of directional selection acting on a large number regions spread across the genome. The number of loci involved in long-term selection response are likely to be in the 100s for a complex trait and that at any point in time selection is likely to simultaneously act on 10s of loci even in populations of limited size. The identified loci are located with high resolution, which makes them obvious candidate regions for attempts to identify causal mutations. The two lines were from the same founder population and were subjected to 50 generations of artificial selection that have led to changes in trait expression and genetics that may resemble those observed from 1000s of years of natural selection. What we observed is genome wide changes that occurred in an accelerated and directed evolution process. In a broader perspective, the results provide not only insights to the effects of artificial selection, but also what may be expected from natural selection when populations adapt to a new environment. This study shows the inherent power and efficiency in combining data from classic long-term selection experiments with modern genomics tools.

## Materials and Methods

### Birds and genotyping

Genotyping was performed on 20 low and 20 high line chickens from generation S40 (the generation of the parents from the F_2_ cross described in Jacobsson et al. [7]), and 10 low and 10 high line chickens from generation S50. At the later time point we chose to genotype an additional 39 individuals from the high line because this line still exhibited a good response to selection, whereas the low line appeared to have phenotypically plateaued. The genotyping was performed by the company DNA Landmarks with the 60 k chicken chip produced by Illumina Inc for the GWMAS Consortium. The animal husbandry for the later generations were the same as described for the previous generations [10]. All procedures involving animals used in this experiment were carried out in accordance with the Virginia Tech Animal Care Committee animal use protocols.

### Simulations

Individual based simulations with parameters chosen to mimic the Virginia lines were performed with a code written in R [31], in order to evaluate the probability of fixation for selected and neutral loci. The number of selected males and females, calculated proportion of selected and selection intensity, *i*, is given in Table S6. For simplicity, the parameters for generation 5–25 in the selection experiment were used for simulation of selection during all 50 generations, because the effective population size for these generations were close to the effective population size for all generations (34.55) (Table S6). The number of females per male was thus 48/12 = 4 and the number of offspring per female was six, which is the number that gives a population size (6×48 = 288) close to the mean population sizes in the selected lines. The selected lines originated from a founder population formed by crossing seven partially inbred (∼36%) lines. We assume that the inbred lines were fixed for all loci, i.e. the starting haplotype frequencies were multiples of 1/7. Simulations were performed with two linked loci, A and B with alleles A/a and B/b, were selection acts on locus A. The fitness of genotypes AA, Aa and, aa were modelled as 1, 1-*hs* and, 1-*s*, respectively, where *s* is the selection coefficient and *h* is used to model dominance. Note that since the selection intensity is different for males and females, there is one selection coefficient for males, *s_M_*, and another for females, *s_F_*, for each locus. Alleles with additive effects (*h* = 0.5) were assumed for the simulations in this paper. The selection coefficient, *s*, for a given QTL was estimated as *s* = *i*2*a*/σ [18], [19]. The selection coefficients for the 11 QTLs with significant additive effects in Jacobsson et al [7] in the low and high line are given in Table S3. The additive effect, *a*, and the phenotypic standard deviation, σ, for the QTLs are as described in Jacobsson et al [7]. Simulations were performed for the QTL with the largest effect (*Growth6* on chromosome 4), the smallest effect (*Growth12* on chromosome 20) and two additional loci (*Growth4* and *Growth9*, on chromosomes 3 and 7 respectively). Fixation in the simulations was defined as all individuals in the simulated population being homozygous for the same allele. This should be kept in mind when comparing with the observed results, where fixation is measured in a genotyped sample form the selected poplation.

### Association mapping

Association mapping was performed using the software package PLINK v1.07 [15]. The results in the manuscript are based on asymptotic p-values from the χ^2^-test (the assoc option in PLINK). As the number of expected in some cells in the χ^2^-test might be small for some SNPs, we have also computed p-values using a Fisher exact test (the fisher option in PLINK) to see that the results did not change due to this. A comparison of the results from using asymptotic p-values with those using a Fisher exact test reveals that even though p-values for individual SNPs are slightly different using the two tests, the overall conclusion does not change.

URL: http://pngu.mgh.harvard.edu/purcell/plink/

### Fixation, heterozygosity, and clusters

Calculations of fixation, observed heterozygosity and clusters were performed in R [31]. The significance of the difference in the decrease in heterozygosity at each locus between generations 40 and 50 in the high and low lines was tested by a two-sided t-test in R (the function t.test). The length of the clusters in cM was calculated using the chromosome specific ratios of cM/Mb given in Table 2 in [17]. The length of the clusters in cM was then transformed to recombination frequency using Haldanes map function. The clusters will contain different alleles in the two lines if no recombination occurred during the fixation process or if recombination occurred only in homozygous individuals ( =  non-informative). The probability for this was calculated as ((1−*r*)+*r*(*p* ^2^+*q* ^2^))^2*Ng*^, where *r* is the recombination frequency between first and last position in the cluster, *p* and *q* are the haplotype frequencies, *N* is the effective population size and *g* is the number of generations until fixation of the cluster. Allele frequencies of *p* = 1/7, *q* = 6/7 and *p* = 3/7, *q* = 4/7 was used in the calculations and 3, 5 and 10 generations was compared.

### Allele frequency changes

Changes in allele frequencies between generations 40 and 50, and also the average over blocks of 5 SNPs were calculated. The mean allele frequency change in each block is compared to the distribution of all blocks across the genome, and if it lies in the 95:th percentile, it is identified as a potential locus under selection. Thus, the number of selected loci per set of 20 blocks is Poission-distributed with average 1, given the assumption that the blocks are independent.

### Quantitative genetic estimation of the number of loci

The total number of loci affecting a trait was estimated using equations 6 (*n* = *D*/(*M*−*T*) and 12 (*T*≈ (*a_min_n_d_* −*M*)/(*n_d_*−1)) in [25]. The estimated number of loci is *n*, *D* is half the phenotypic difference between the parental lines (here 670.5), *M* is the average additive effect of the detected loci, *T* is the detection threshold, *a_min_* is the smallest additive effect among the detected loci, and *n_d_* is the number of detected loci. Data on additive effects from previously identified QTLs were from Table 3 in [17]. The estimation was done for the body weight traits with at least 3 identified QTLs.

## Supporting Information

Figure S1 Results for all chromosomes for an association test on allele frequency differences between generation 40 and 50 in the high line (red) and the low line (blue). The result for individual SNPs are shown as circles. The grey line indicates the Bonferroni corrected significance level p<0.001, and the dashed grey line p<0.05. For the low line there are significant differences (p<0.05) on chromosome 12 and 15. (Using a Fisher exact test the region on chromosome 12 is not significant with Bonferroni correction but instead a region on chromosome 11 is significant). For the high line there are significant differences at chromosomes 1 (0.001), 2 (0.001), 3 (0.001), 4 (0.05), 5 (0.001), 6 (0.001), 7 (0.001), 8 (0.001), 9 (0.001), 10 (0.05), 12 (0.05), 14 (0.05), 18 (0.001), 20 (0.05), 21 (0.05), 22 (0.001). Using a Fisher exact test the regions on chromosomes 4, 12, and 20 are not significant with Bonferroni correction.
(0.40 MB PDF)

Figure S2 Results for all chromosomes for an association test on allele frequency differences between the high- and low line. The result for individual SNPs are shown as circles and a sliding window mean of 20 markers are shown as a red line for generation 40 and as a purple line for generation 50. The dashed lines indicate the maximum χ^2^ values, which is obtained when a SNP is fixed for different alleles in the high and low line (80 and 118, respectively). The grey line indicates the Bonferroni corrected significance level at p<0.001.
(0.97 MB PDF)

Figure S3 Allele frequency changes between generation 40 and 50 across all chromosomes. Each symbol corresponds to the average frequency change over a block of 5 markers. Colored symbols indicate blocks that belong to the 95:th percentile, compared to all blocks in the entire genome. The blue and red lines indicate the number of outliers present in a window of 20 blocks. The largest changes are in several regions on chromosomes 1, 2 and 3 as well as in regions on chromosomes 7, 9, 11, 12, 18, 20 and 22. Changes on chromosomes 9 and 12 are most prominent in the high line, whereas changes on chromosome 2, 3 and 11 are mostly in the low line. Chromosome 20 is affected in both lines, but in the high line the changes are located towards the end of the chromosome and in the low line the first third is changing rapidly. Similarly, chromosome 22 has distinct regions affected in the different lines. On chromosome 18 a region changes rapidly in both lines, although the favoured allele differs for many, but not all, affected markers.
(0.37 MB PDF)

Figure S4 Simulations in the high line with *h* = 0.5 and selection coefficients from A) *Growth4*, B) *Growth6*, C) *Growth9*, and D) *Growth12* with starting haplotype frequencies of 4003, i.e. 4/7 AB and 3/7 ab. The selection is strong enough to always lead to fixation at locus A except for *Growth12* where fixation is reached in around 85% of replicates. A linked neutral locus often reaches fixation at recombination frequencies below 1–2 cM. The probability of fixation at the linked but unselected locus B is affected by the initial haplotype frequencies. A higher frequency from the beginning leads to a higher probability of fixation.
(0.04 MB PDF)

Figure S5 Simulations with no selection shows that fixation only occurs in 10–20% of the replicates for initial haplotype frequencies of 1006 i.e. 1/7 AB and 6/7 ab (A) and around 50% for initial haplotype frequencies of 3004 i.e. 3/7 AB and 4/7 ab (B).
(0.03 MB PDF)

Figure S6 Distribution of estimated effects in the original F_2_ population. Distribution of the additive effect estimates in the genome scan for QTL affecting body weight at 56 days of age in an F_2_ intercross between the high- and low- Virginia lines. The effects are given on a natural log-scale and ordered by size. The solid line shows the linear logarithmic trend for the effects in the range 10–30. This illustrates that the relative difference between the ordered genetic effects is close to constant. At both ends of the distribution, the differences between the neighbouring effects is greater, which could indicate that these are over- and under- estimates of effects due to sampling. During selection, this distribution indicates that there will always be a smaller set of loci (often 5–10) that will have be dominant over the rest in mediating response to selection, given that the relation between the effects does not change as larger effects go to fixation.
(0.03 MB PDF)

Table S1 Fixation in the low and high line in different generations and sample sizes. The number of fixed alleles is dependent on the sample size, and thus results based on the total number of genotyped individuals is not directly comparable since the number of sampled individuals is not the same at the two time points. For comparison, fixation was also computed in a random sample of 10 individuals from each of the lines at both time points and the general trend is very similar regardless of the sample size. The number of SNPs fixed for different alleles has increased by 75.0% (whole data set) or 63.8% (10+10 individuals) during the ten generations. The number of SNPs fixed for the same allele has increased by 6.7% (whole data set) or 6.6% (10+10 individuals) during the ten generations.
(0.03 MB PDF)

Table S2 Fixation dynamics during 10 generations, depending on sample size. The numbers indicate the number of SNPs that are present in the two compared sets. The set for generation 40 is given before the arrow and generation 50 after the arrow. Comparison with Table S1 shows that the majority of the fixed alleles in generation 40 also are fixed in generation 50, indicating that we have large enough sample size to accurately estimate fixed alleles. *Diff*  =  fixed for different alleles in the high and low line, *Same*  =  fixed for same allele, *H not L* =  fixed in high but not in low, *L not H* =  fixed in low but not high.
(0.03 MB PDF)

Table S3 Selection coefficients for the QTLs in the body weight selected lines, calculated using *i* from generation 5–25 and additive effects for body weight at 56 days from [7] and [17].
(0.03 MB PDF)

Table S4 Number of generations until fixation for different QTLs. All simulations have starting frequencies 4/7 AB and 3/7 ab. A single additive QTL is assumed. H denotes the high line and L the low line.
(0.04 MB PDF)

Table S5 Number of generations until fixation, for different starting frequencies at *Growth9* in the high line, assuming additive QTL effects. Our notation for the starting allele frequencies of two loci A and B with alleles A/a and B/b is a four digit code xyzw, where x is the proportion of haplotype AB, y is the proportion of haplotype Ab, z is the proportion of haplotype aB and w is the proportion of haplotype ab.
(0.02 MB PDF)

Table S6 Population parameters for the body weight selected lines, *p* is proportion selected, *i* is selection intensity calculated from *p*. *p* was calculated separately for males and females by dividing the number of selected by the average number of individuals in each generation (*n* = 268 in the high line and *n* = 309 in the low line, and assuming equal sex ratio in the offspring). The selection intensities, *i*, were retrieved from *p* using the tables on pp 379–380 in Falconer and Mackay [18]. Since the number of males and females selected in each generation are not equal, *i* is different for males and females leading to different *s* for males and females. The effective population size for each of the three generation intervals was estimated as 4*N_m_*N_f_/(*N_m_*+*N_f_*). The effective population size for generations 1–40 estimated as the harmonic mean is 40/((4/27.43)+(26/38.40)+(15/44.80))  = 34.55, whereas until generation 50 it is 40/((4/27.43)+(26/38.4)+(25/44.80))  = 36.21.
(0.03 MB PDF)

Table S7 Clusters as defined by different maximum distance between, and minimum number of, SNPs.
(0.03 MB PDF)

Table S8 Number of clusters with at least 5 SNPs fixed for different alleles in the two lines in generation 40 and 50, together with their length distribution.
(0.02 MB PDF)

Table S9 Number of clusters with at least 2 SNPs fixed for different alleles in the two lines in generation 40 and 50, together with their length distribution.
(0.02 MB PDF)

## References

[1] Maynard Smith J, Haigh J (1974). The hitch-hiking effect of a favourable gene.. Genet Res.

[2] Berry AJ, Ajioka JW, Kreitman M (1991). Lack of polymorphism on the Drosophila fourth chromosome resulting from selection.. Genetics.

[3] Orr HA, Betancourt AJ (2001). Haldane's sieve and adaptation from the standing genetic variation.. Genetics.

[4] Przeworski M, Coop G, Wall JD (2005). The signature of positive selection on standing genetic variation.. Evolution.

[5] Hermisson J, Pennings PS (2005). Soft sweeps: molecular population genetics of adaptation from standing genetic variation.. Genetics.

[6] Pennings PS, Hermisson J (2006a). Soft sweeps II – molecular population genetics of adaptation from recurrent mutation or migration.. Mol Biol Evol.

[7] Jacobsson L, Park HB, Wahlberg P, Fredriksson R, Perez-Enciso M (2005). Many QTLs with minor additive effects are associated with a large difference in growth between two selection lines in chickens.. Genet Res.

[8] Hill WG (2005). A century of corn selection.. Science.

[9] Hill WG, Bunger L (2004). Inferences on the genetics of quantative traits from long-term selection in laboratory and domestic animals.. Plant Breeding Rev.

[10] Dunnington EA, Siegel PB (1996). Long-term divergent selection for eight-week body weight in White Plymouth Rock chickens.. Poult Sci.

[11] Sabeti PC, Varilly P, Fry B, Lohmueller J, Hostetter E (2007). Genome-wide detection and characterization of positive selection in human populations.. Nature.

[12] Pennings PS, Hermisson J (2006b). Soft sweeps III: the signature of positive selection from recurrent mutation.. PLoS Genetics.

[13] Teotónio H, Chelo IM, Bradić M, Rose MR, Long AD (2009). Experimental evolution reveals natural selection on standing genetic variation.. Nat Genet.

[14] Raquin A-L, Brabant P, Rhoné B, Balfourier F, Leroy P (2008). Soft selective sweep near a gene that increases plant height in wheat.. Mol Ecol.

[15] Purcell S, Neale B, Todd-Brown K, Thomas L, Ferreira MAR (2007). PLINK: a toolset for whole-genome association and population-based linkage analysis.. Am J Hum Genet.

[16] Carlborg Ö, Jacobsson L, Åhgren P, Siegel P, Andersson L (2006). Epistasis and the release of genetic variation during long-term selection.. Nat Genet.

[17] Wahlberg P, Carlborg Ö, Foglio M, Tordir X, Syvänen A-C (2009). Genetic analysis of an F2 intercross between two chicken lines divergently selected for body-weight.. BMC Genomics.

[18] Falconer DS, Mackay TFC (1996). Introduction to Quantitative Genetics. 4th ed..

[19] Kimura M, Crow JF (1978). Effect of overall phenotypic selection on genetic change at individual loci.. Proc Natl Acad Sci USA.

[20] Marquez GL, Lewis RM, Wiegland EN, Siegel PB (2009). Inbreeding and population structure in lines of chickens divergently selected for high and low 8-week body weight.. Poultry Science.

[21] Gillespie JH (1998). Population Genetics: A Concise Guide..

[22] Castle WE (1921). An improved method of estimating the number of genetic factors concerned in cases of blending inheritance.. Proc Natl Acad Sci USA.

[23] Wright S (1968). Evolution and the Genetics of Populations: Volume 1, Genetic and biometric foundations..

[24] Zeng ZB (1992). Correcting the bias of Wright estimates of the number of genes affecting a quantitative character—a further improved method.. Genetics.

[25] Otto SP, Jones CD (2000). Detecting the undetected: Estimating the total number of loci underlying a quantitative trait.. Genetics.

[26] Buckler ES, Holland JB, Bradbury PJ, Acharya CB, Brown PJ (2009). The genetic architecture of maize flowering time.. Science.

[27] Laurie CC, Chasalow SD, LeDeaux JR, McCarroll R, Bush D (2004). The genetic architecture of response to long-term artificial selection for oil concentration in the maize kernel.. Genetics.

[28] Weedon MN, Lango H, Lindgren CM, Wallace C, Evans DM (2008). Genome-wide association analysis identifies 20 loci that influence adult height.. Nat Genet.

[29] Park H-B, Jacobsson L, Wahlberg P, Siegel PB, Andersson L (2006). QTL analysis of body composition and metabolic traits in an intercross between chicken lines divergently selected for growth.. Physiol Genomics.

[30] Rubin CJ, Zody MC, Eriksson J, Meadows JR, Sherwood E (2010). Whole-genome resequencing reveals loci under selection during chicken domestication.. Nature.

[31] R Development Core Team (2007). R: A language and environment for statistical computing..

