# Supplementary material for: Genome-Wide Effects of Long-Term Divergent Selection
Source: PLoS Genet. 2010 Nov 4;6(11):e1001188. doi: 10.1371/journal.pgen.1001188 (PMC2973821; doi:10.1371/journal.pgen.1001188)
Supplement: Figure S1 — Results for all chromosomes for an association test on allele frequency differences between generation 40 and 50 in the high line (red) and the low line (blue). The result for individual SNPs are shown as circles. The grey line indicates the Bonferroni corrected significance level p<0.001, and the dashed grey line p<0.05. For the low line there are significant differences (p<0.05) on chromosome 12 and 15. (Using a Fisher exact test the region on chromosome 12 is not significant with Bonferroni correction but instead a region on chromosome 11 is significant). For the high line there are significant differences at chromosomes 1 (0.001), 2 (0.001), 3 (0.001), 4 (0.05), 5 (0.001), 6 (0.001), 7 (0.001), 8 (0.001), 9 (0.001), 10 (0.05), 12 (0.05), 14 (0.05), 18 (0.001), 20 (0.05), 21 (0.05), 22 (0.001). Using a Fisher exact test the regions on chromosomes 4, 12, and 20 are not significant with Bonferroni correction. (0.40 MB PDF) [file pgen.1001188.s001.pdf]

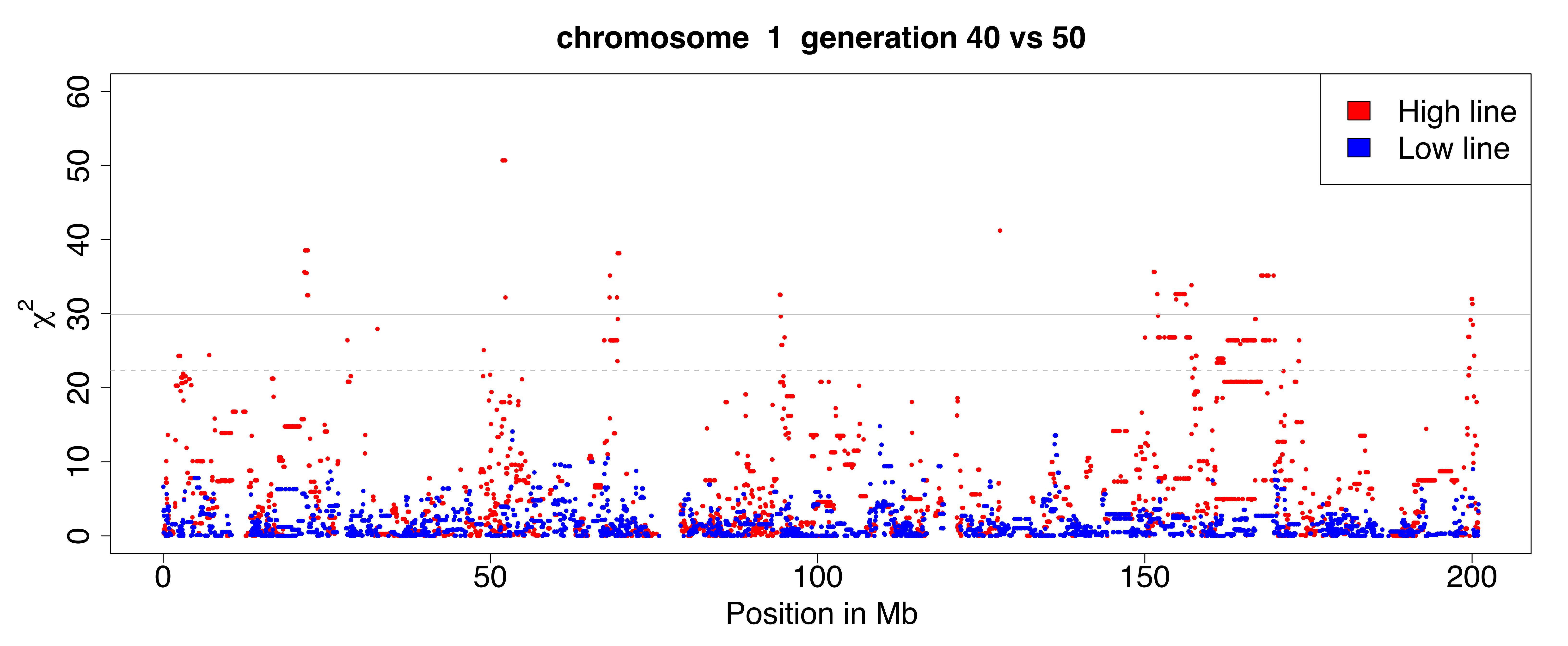
chromosome 1 generation 40 vs 50
High line
Low line
χ^2
0
10
20
30
40
50
60
0
50
100
150
200
Position in Mb

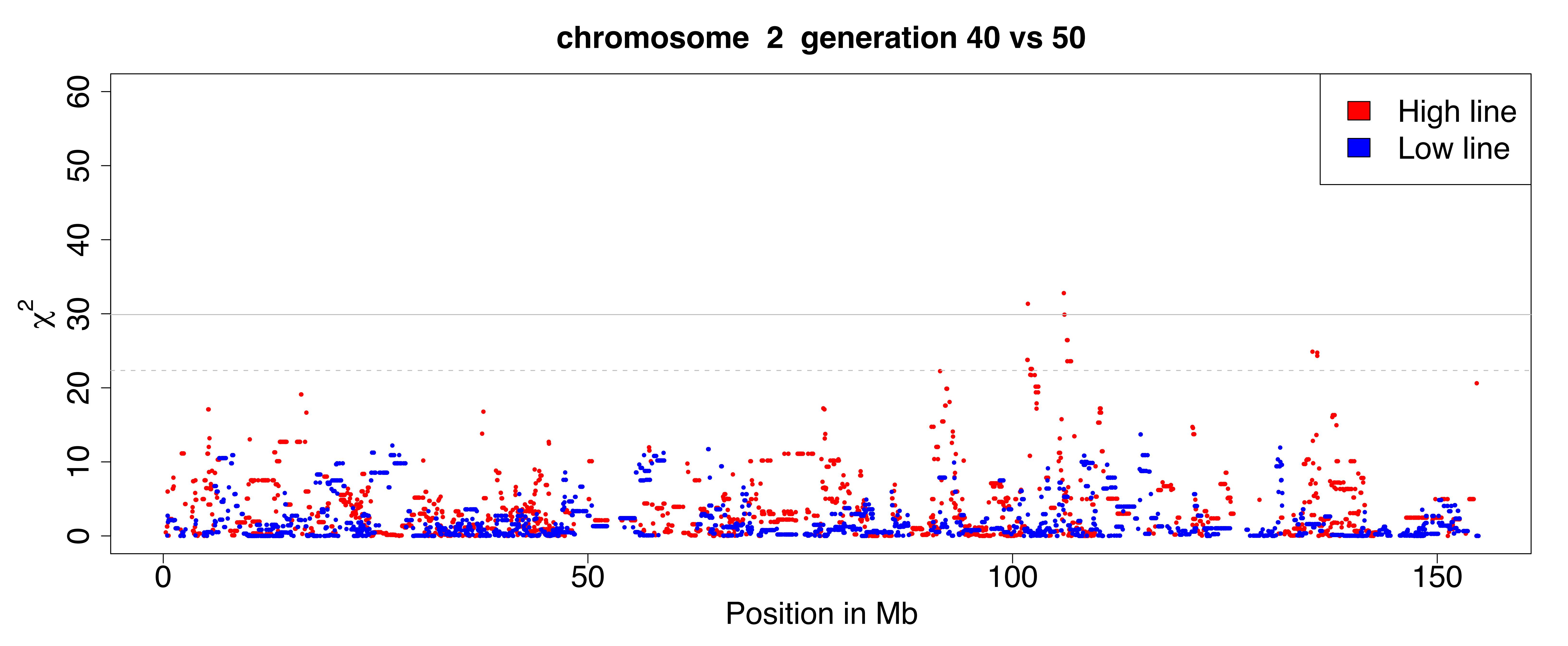

chromosome 2 generation 40 vs 50
High line
Low line
χ^2
0
10
20
30
40
50
60
0
50
100
150
Position in Mb

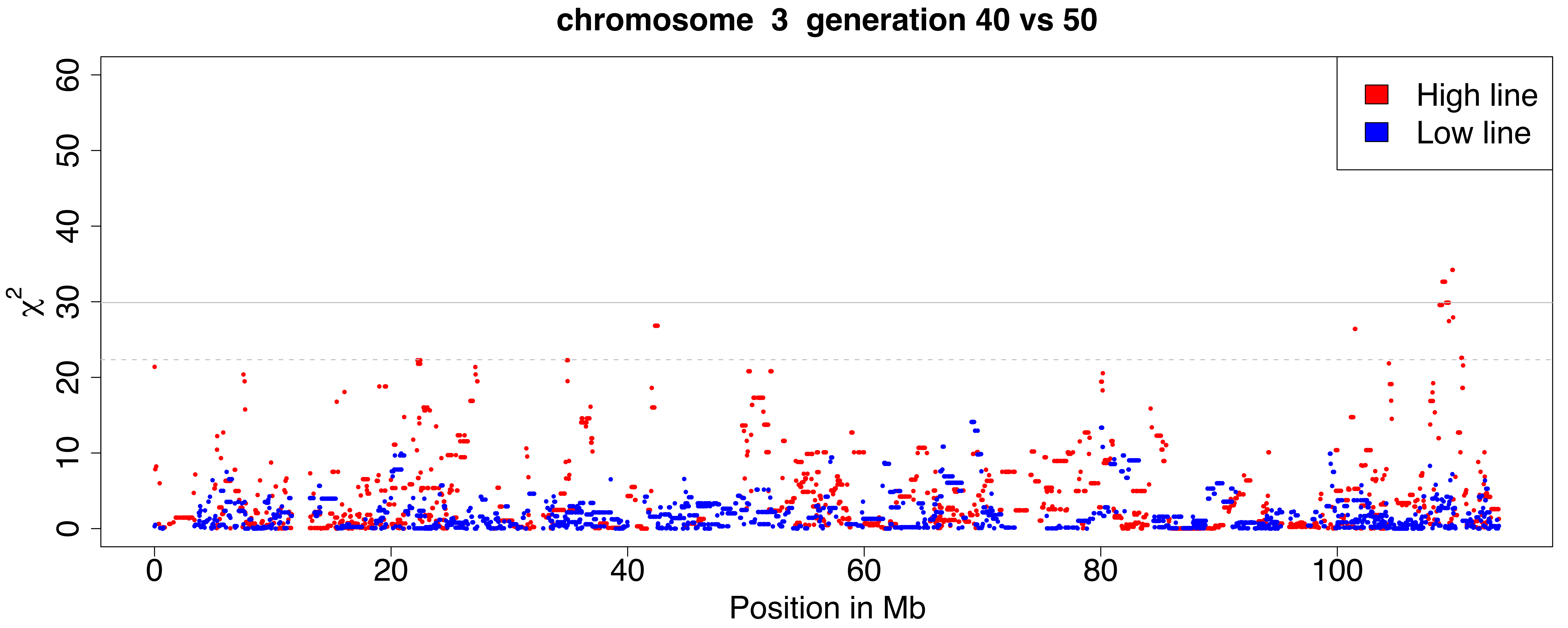

chromosome 3 generation 40 vs 50
High line
Low line
χ^2
0
10
20
30
40
50
60
0
20
40
60
80
100
Position in Mb

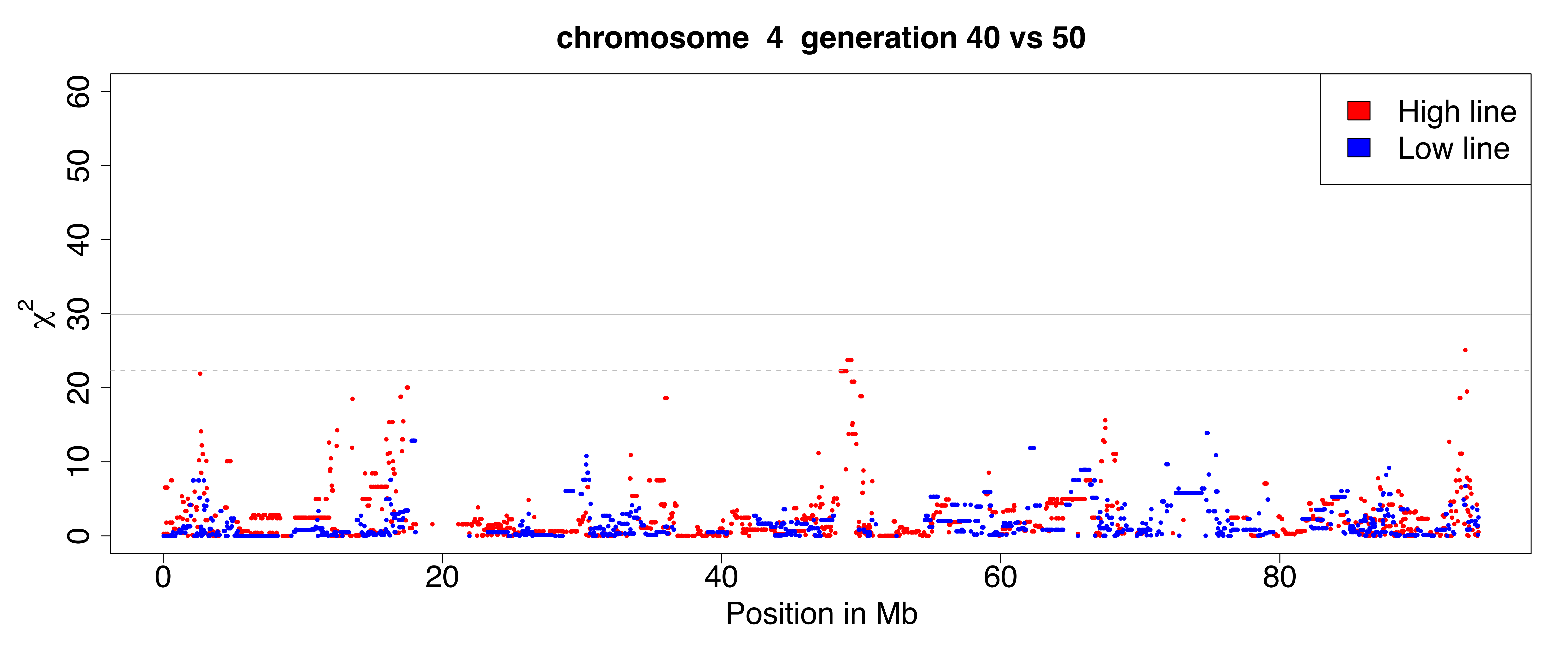
chromosome 4 generation 40 vs 50
High line
Low line
χ^2
0
10
20
30
40
50
60
0
20
40
60
80
Position in Mb

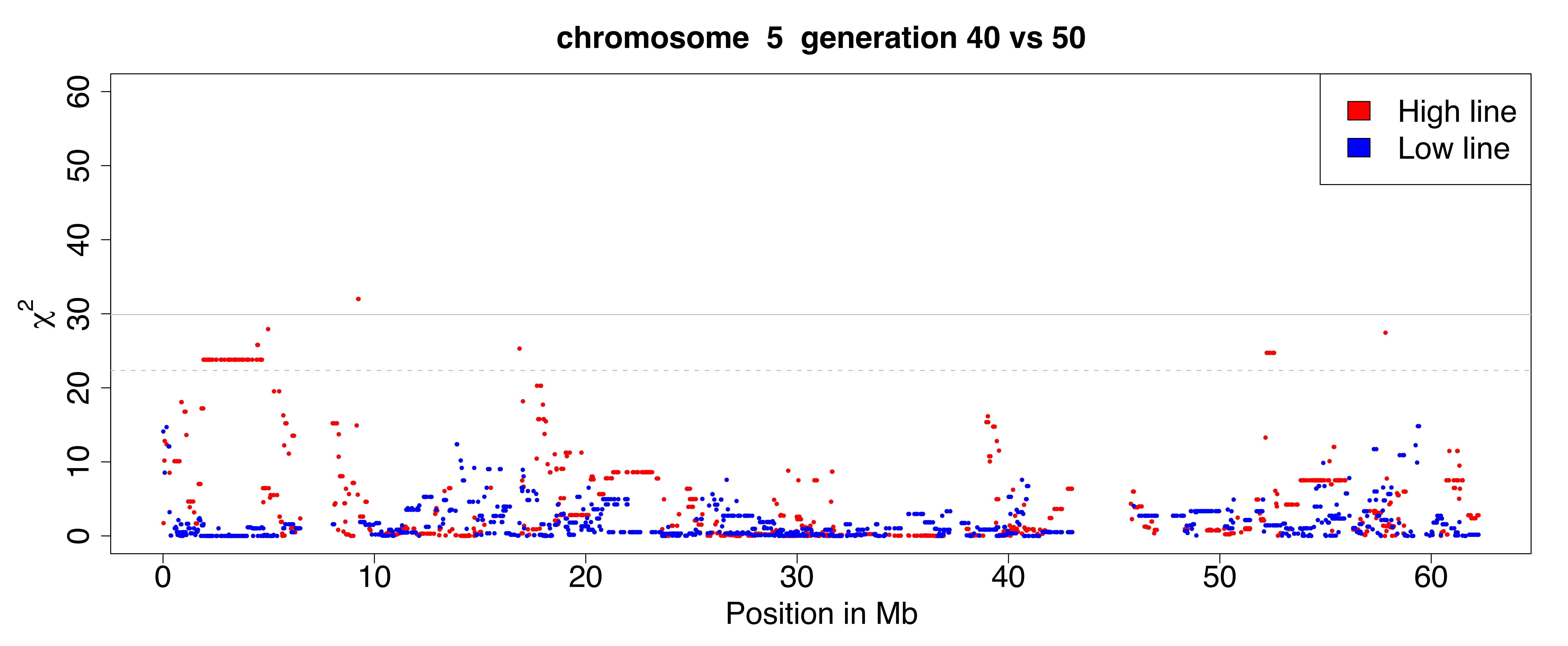
chromosome 5 generation 40 vs 50
High line
Low line
χ2
Position in Mb
0
10
20
30
40
50
60

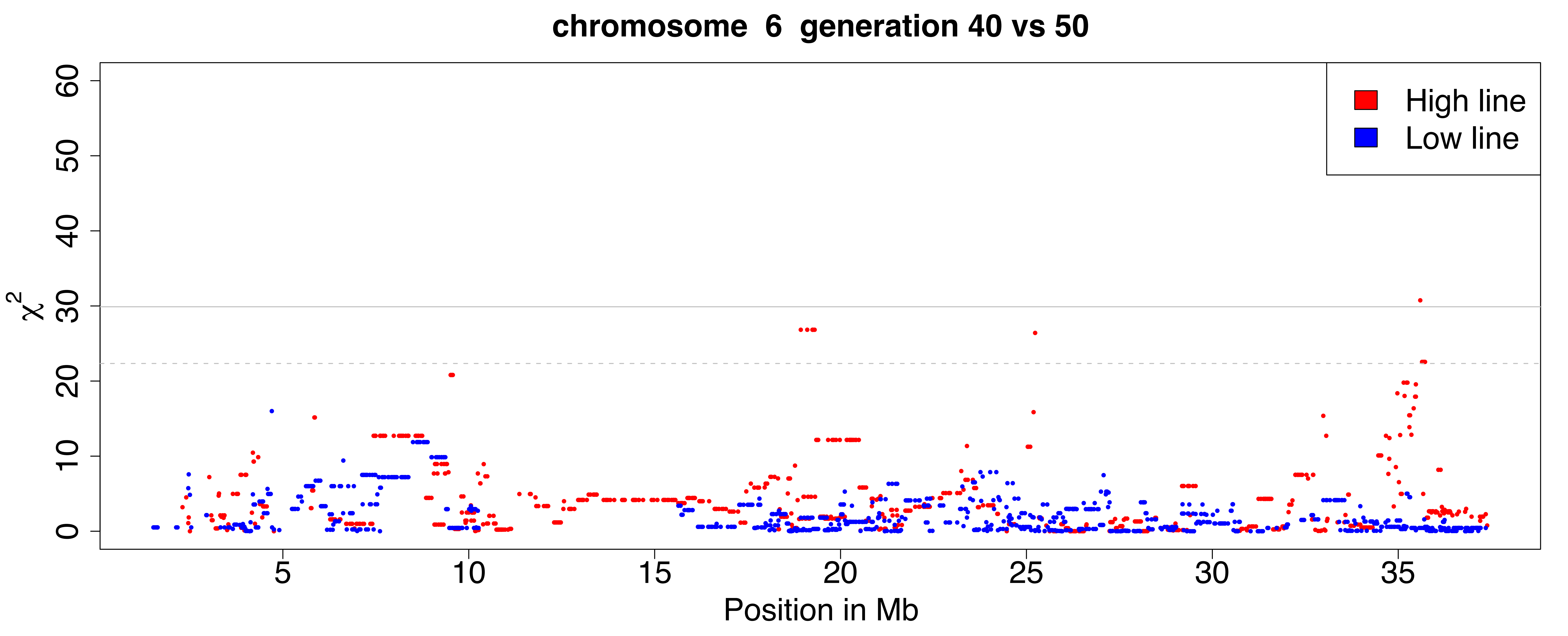
chromosome 6 generation 40 vs 50
High line
Low line
χ^2
0
10
20
30
40
50
60
5
10
15
20
25
30
35
Position in Mb

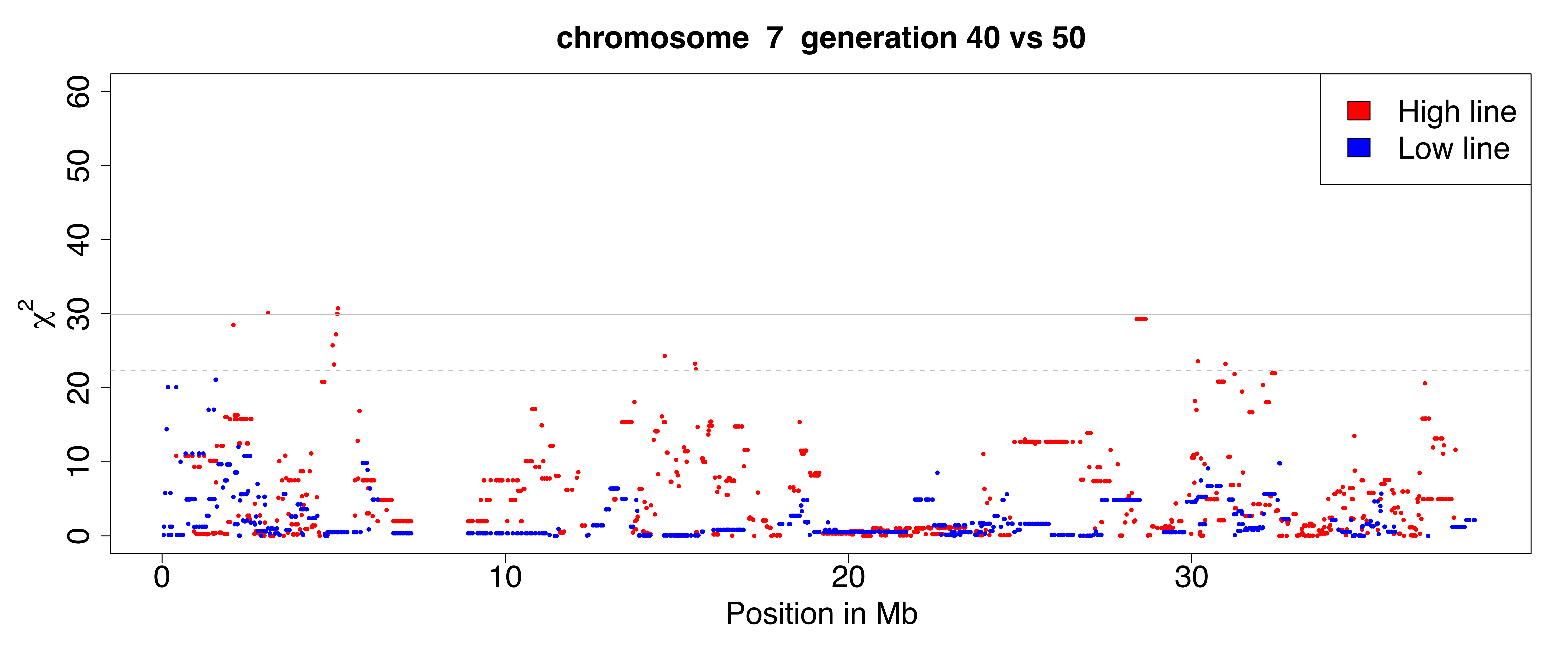
chromosome 7 generation 40 vs 50
High line
Low line
χ^2
0
10
20
30
40
50
60
0
10
20
30
Position in Mb

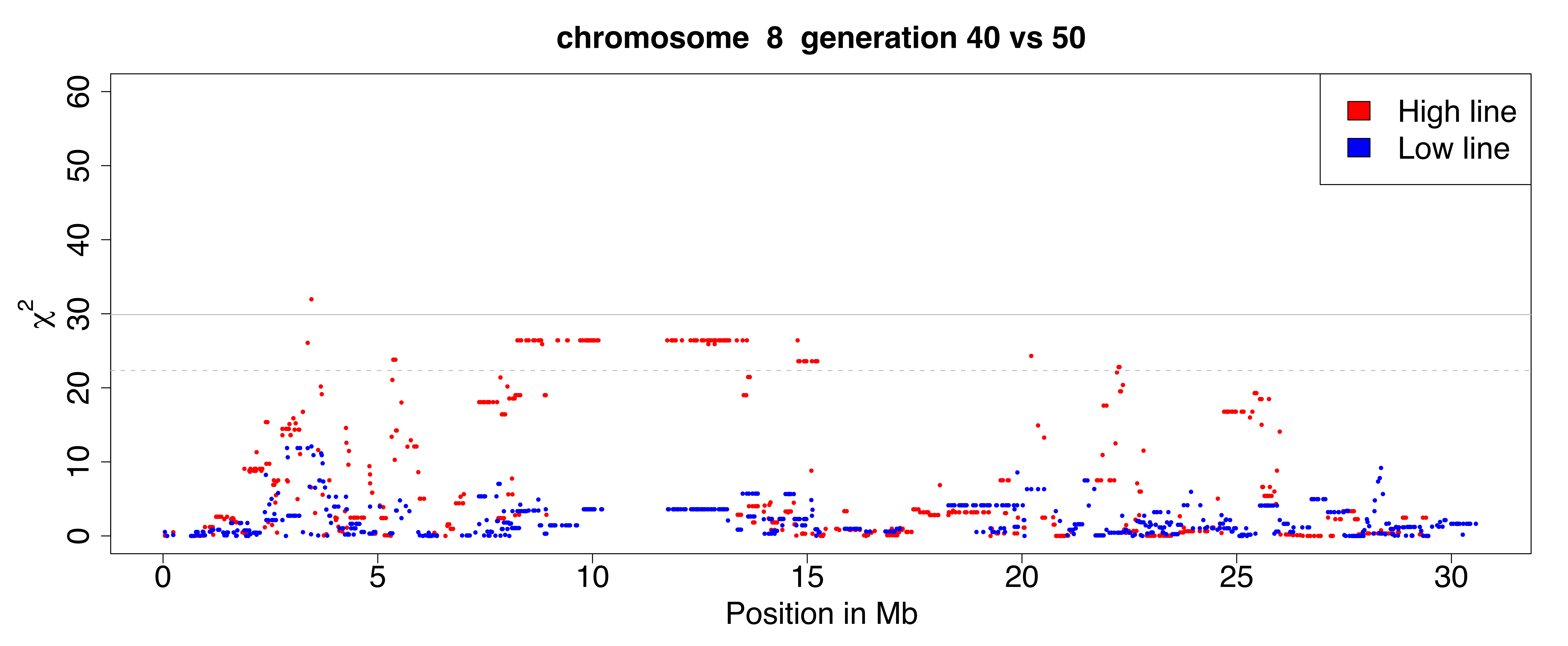
chromosome 8 generation 40 vs 50
High line
Low line
χ^2
0
10
20
30
40
50
60
0
5
10
15
20
25
30
Position in Mb

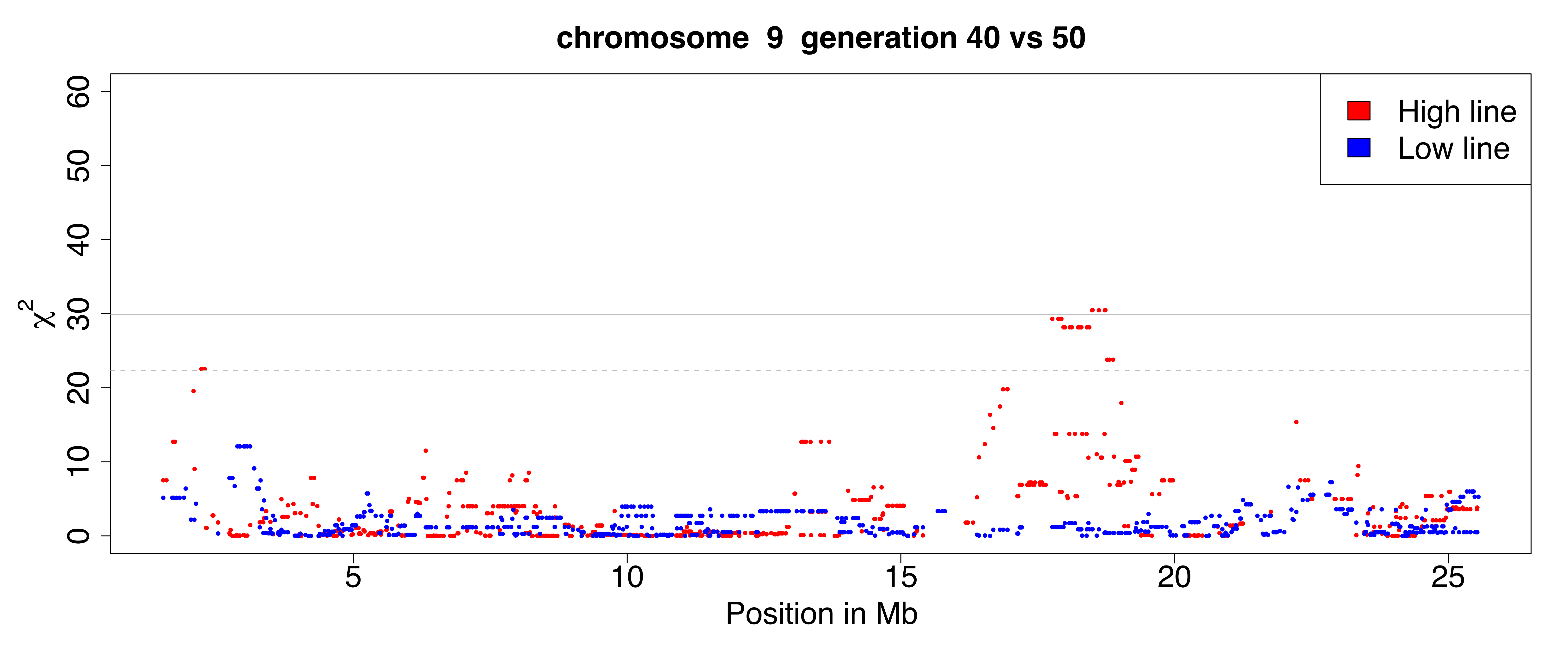

chromosome 9 generation 40 vs 50
High line
Low line
χ^2
0
10
20
30
40
50
60
5
10
15
20
25
Position in Mb

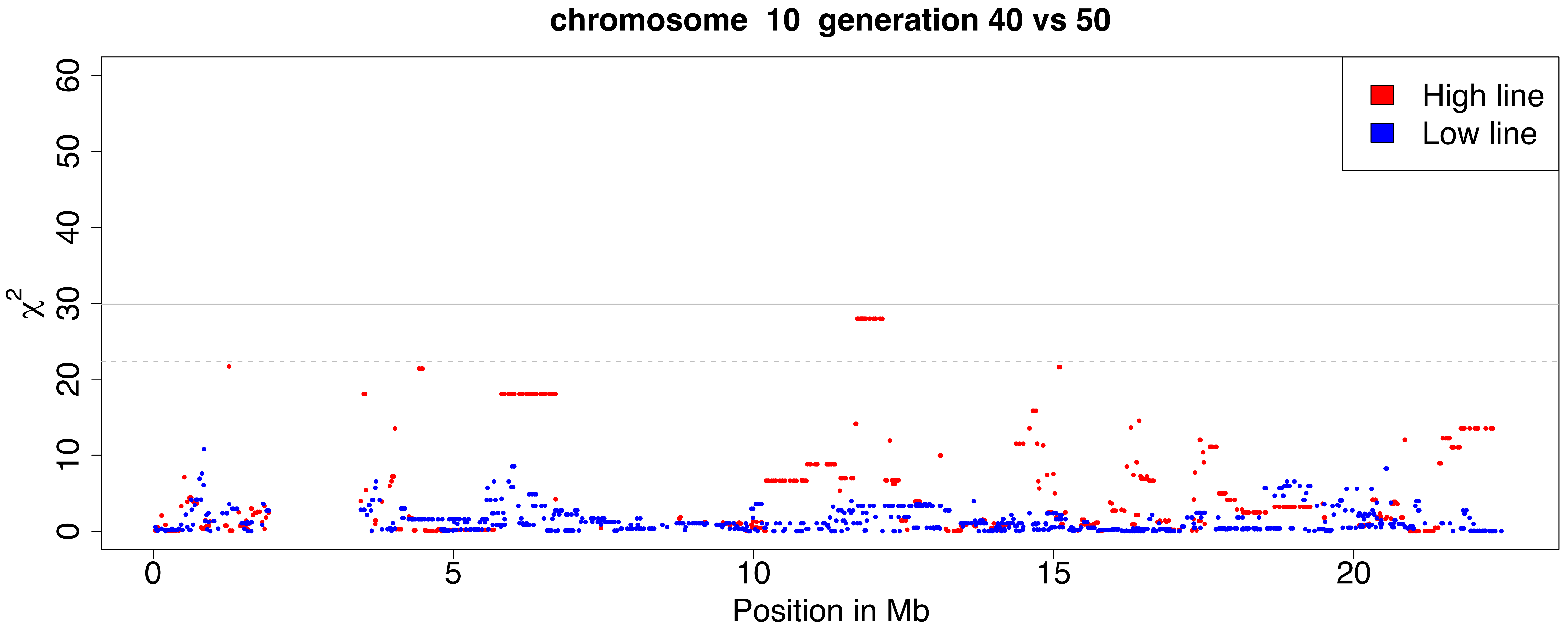

chromosome 10 generation 40 vs 50
High line
Low line
χ^2
0
10
20
30
40
50
60
0
5
10
15
20
Position in Mb

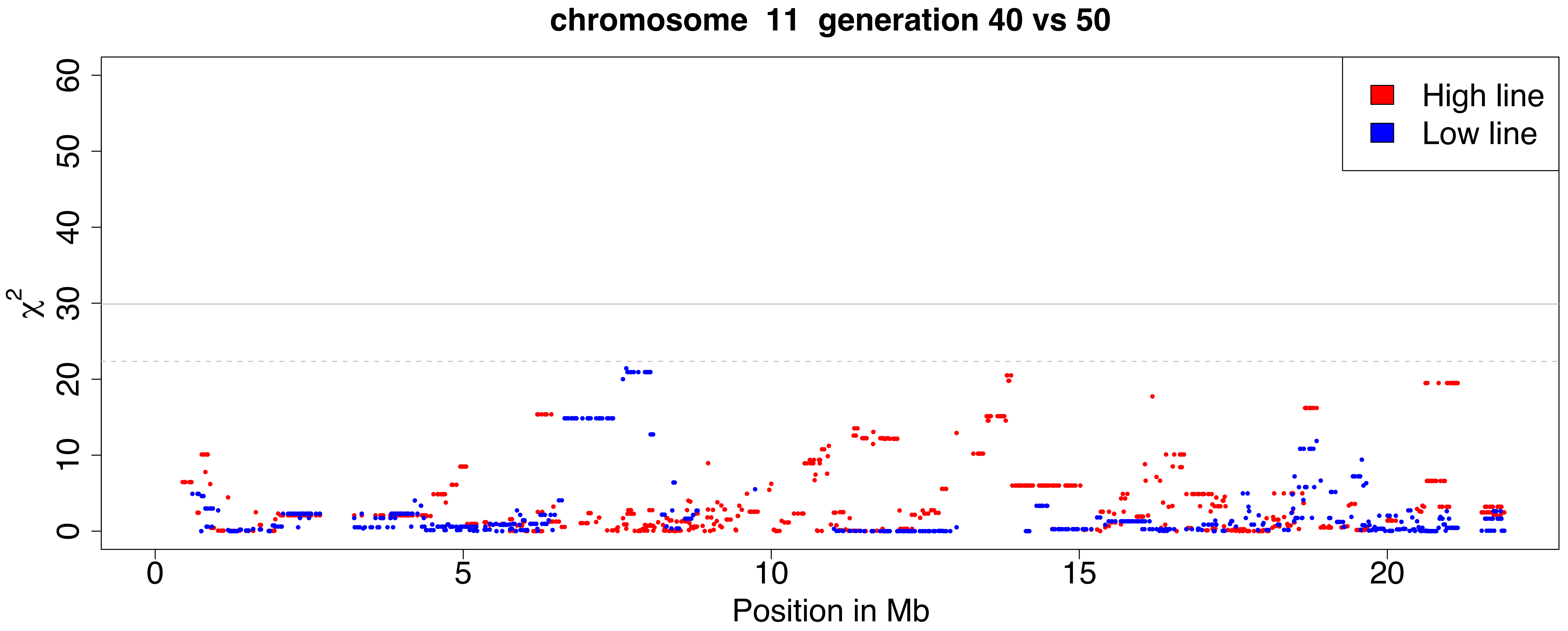
chromosome 11 generation 40 vs 50
High line
Low line
χ^2
0
10
20
30
40
50
60
0
5
10
15
20
Position in Mb

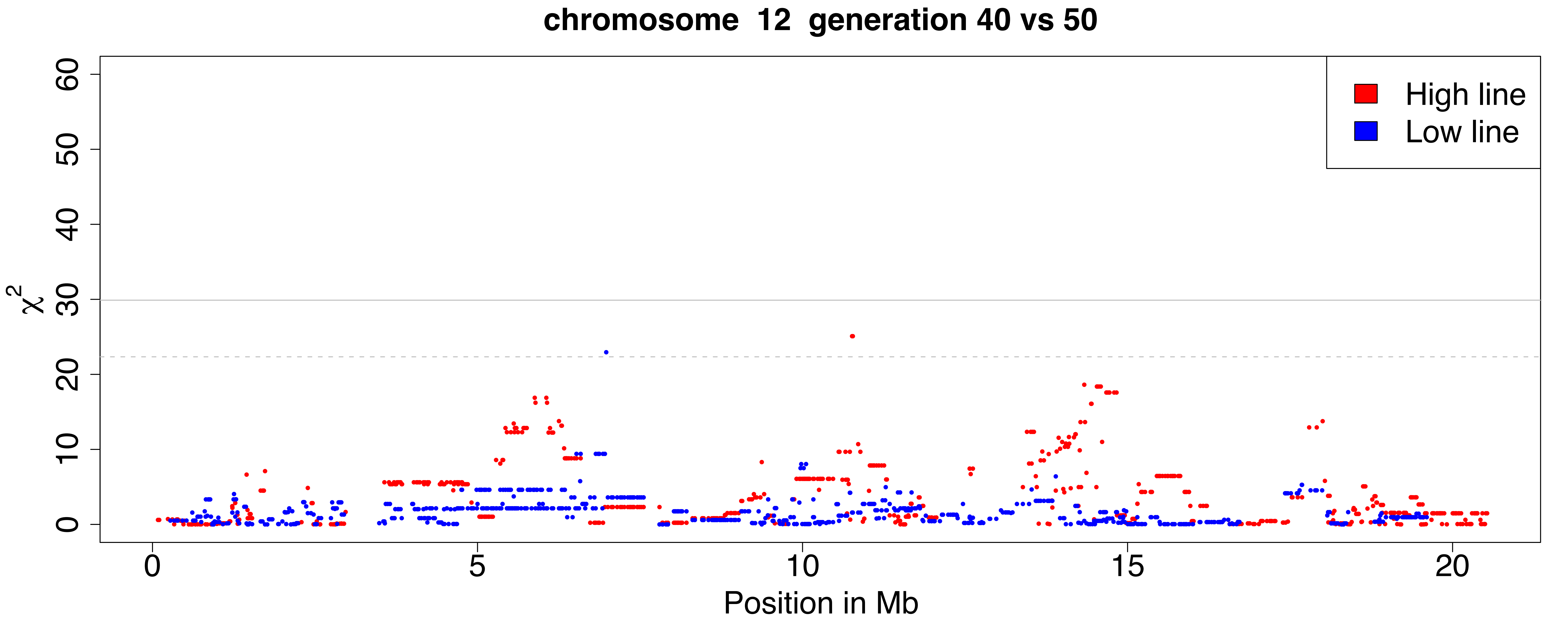

chromosome 12 generation 40 vs 50
High line
Low line
χ^2
0
10
20
30
40
50
60
0
5
10
15
20
Position in Mb

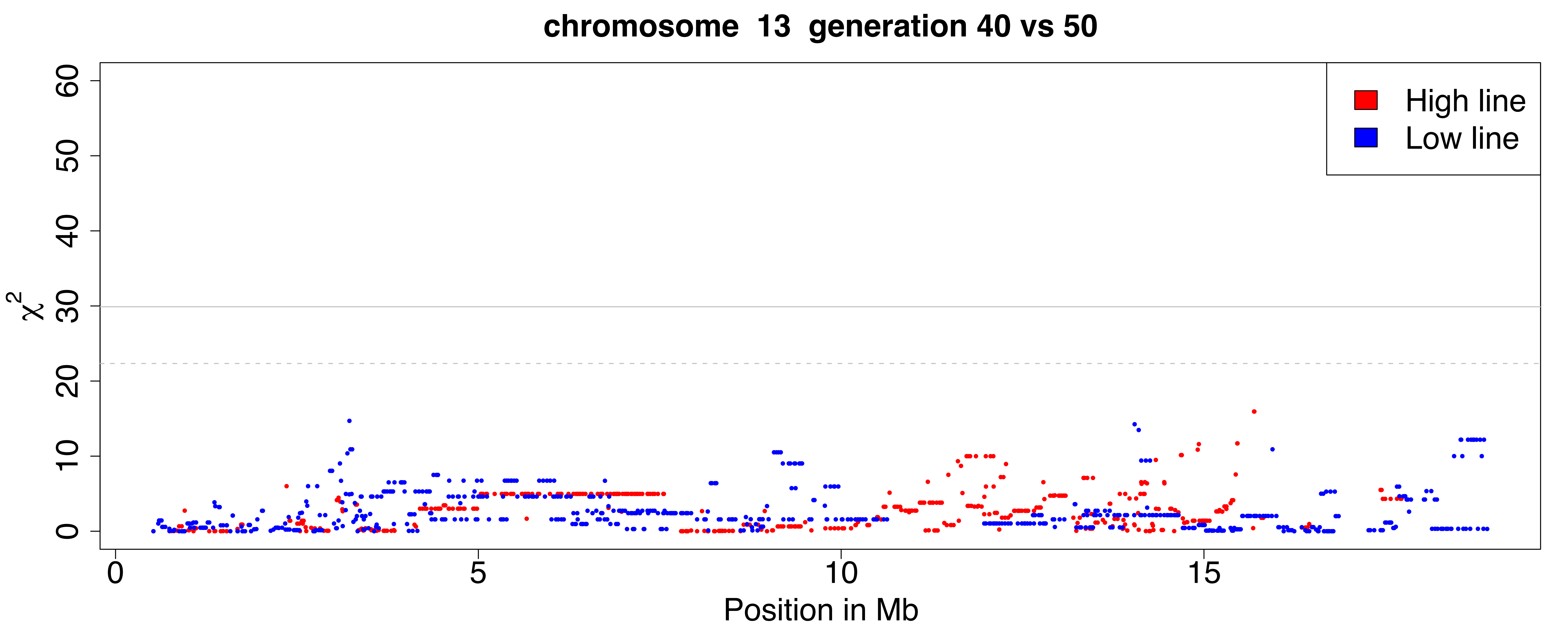

chromosome 13 generation 40 vs 50
High line
Low line
χ^2
0
10
20
30
40
50
60
0
5
10
15
Position in Mb

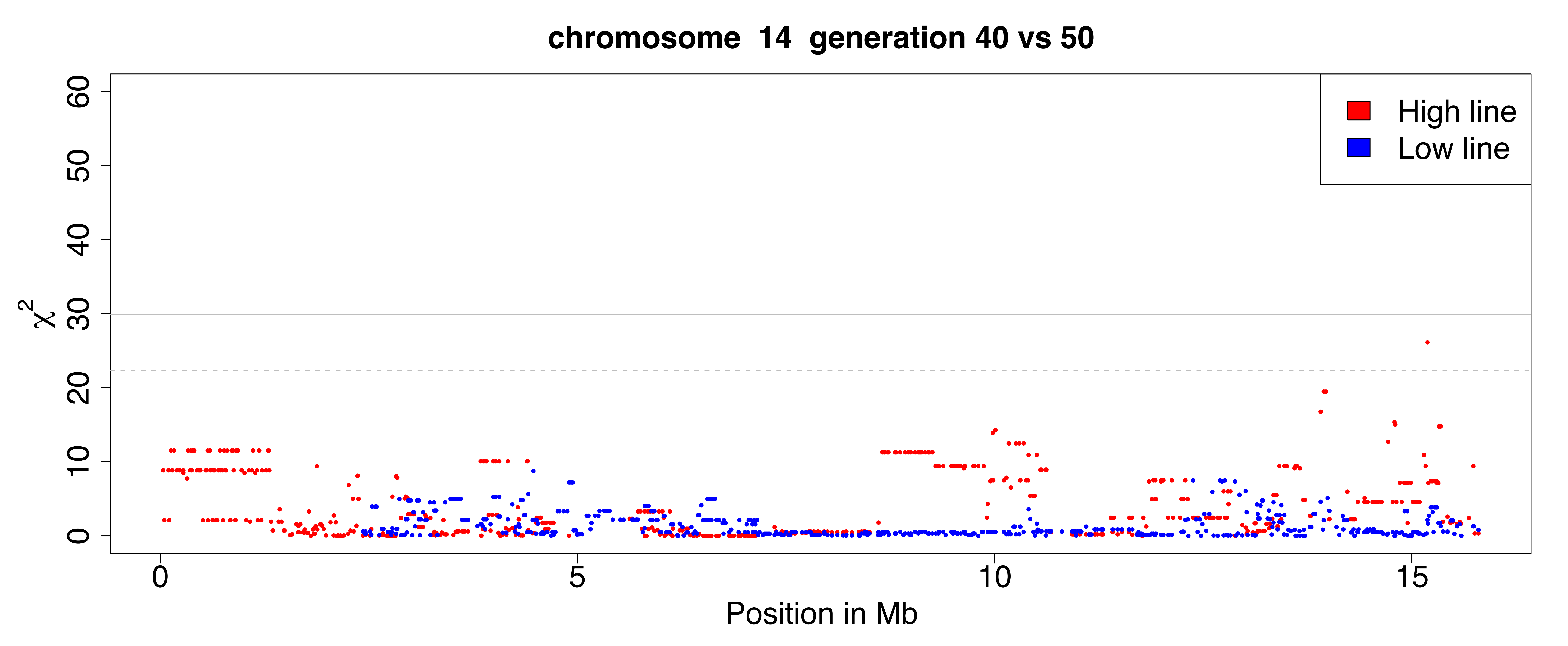

chromosome 14 generation 40 vs 50
High line
Low line
0
10
20
30
40
50
60
χ2
0
5
10
15
Position in Mb

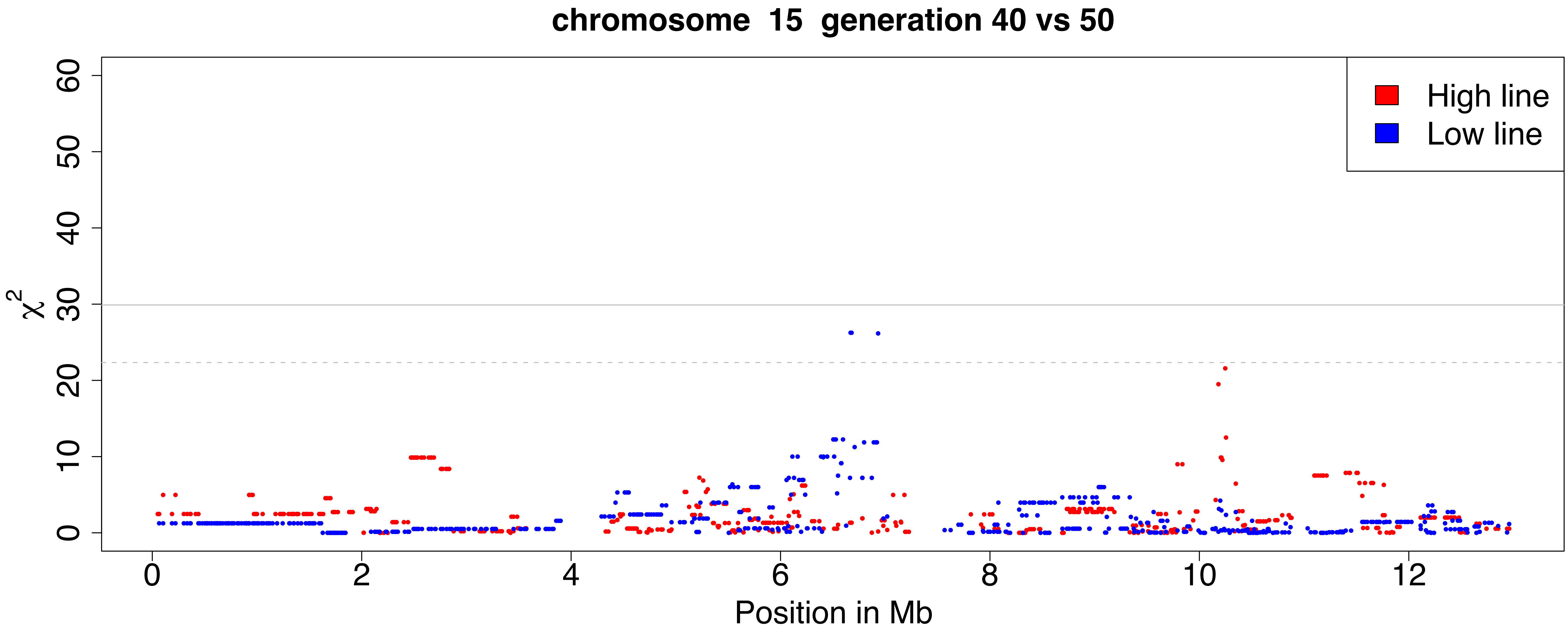

chromosome 15 generation 40 vs 50
High line
Low line
χ^2
0
10
20
30
40
50
60
0
2
4
6
8
10
12
Position in Mb

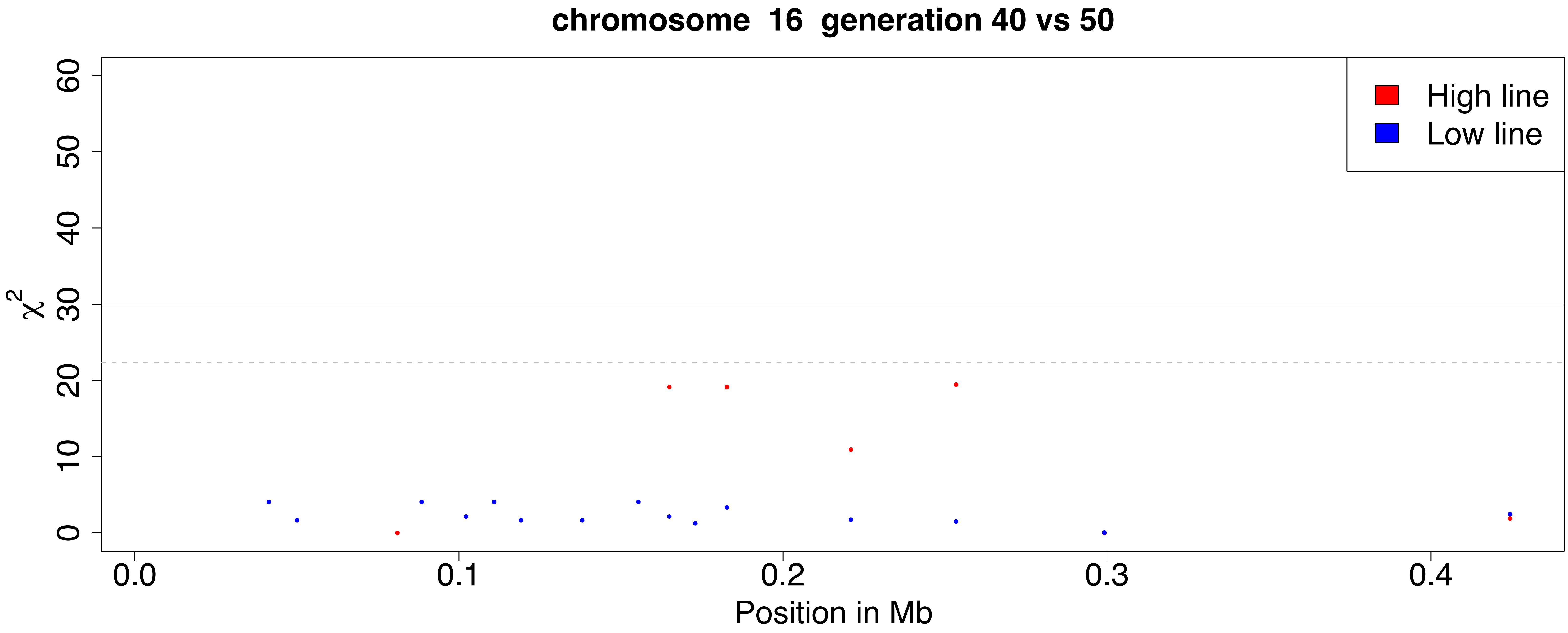
chromosome 16 generation 40 vs 50
High line
Low line
χ^2
0
10
20
30
40
50
60
0.0
0.1
0.2
0.3
0.4
Position in Mb

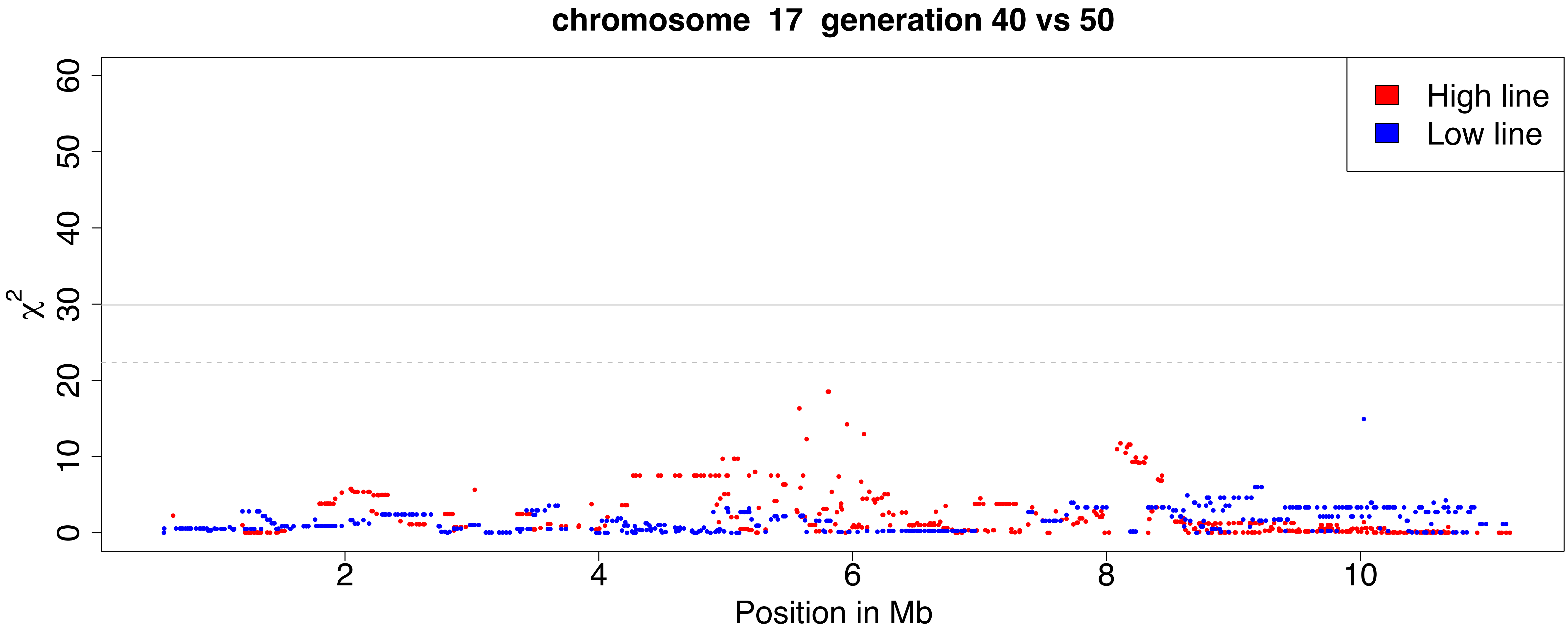
chromosome 17 generation 40 vs 50
High line
Low line
χ^2
0
10
20
30
40
50
60
2
4
6
8
10
Position in Mb

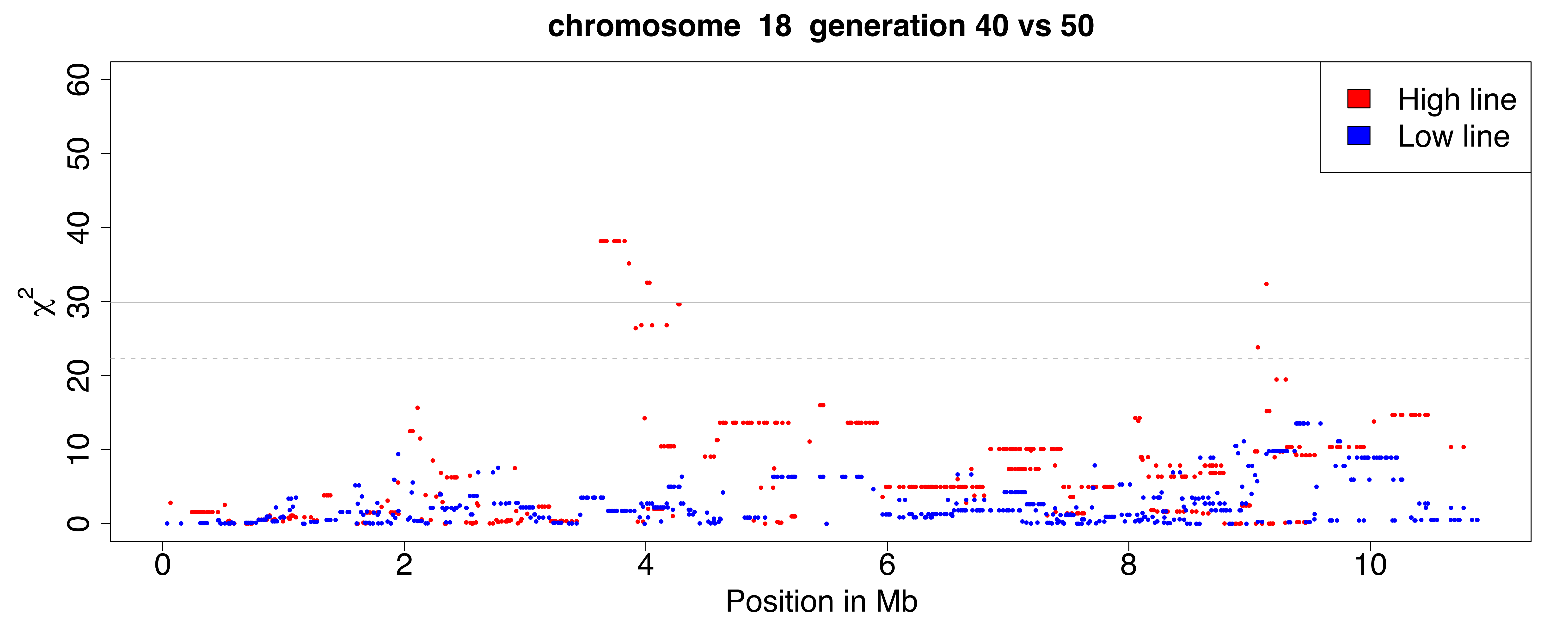

chromosome 18 generation 40 vs 50
High line
Low line
χ^2
0
10
20
30
40
50
60
0
2
4
6
8
10
Position in Mb

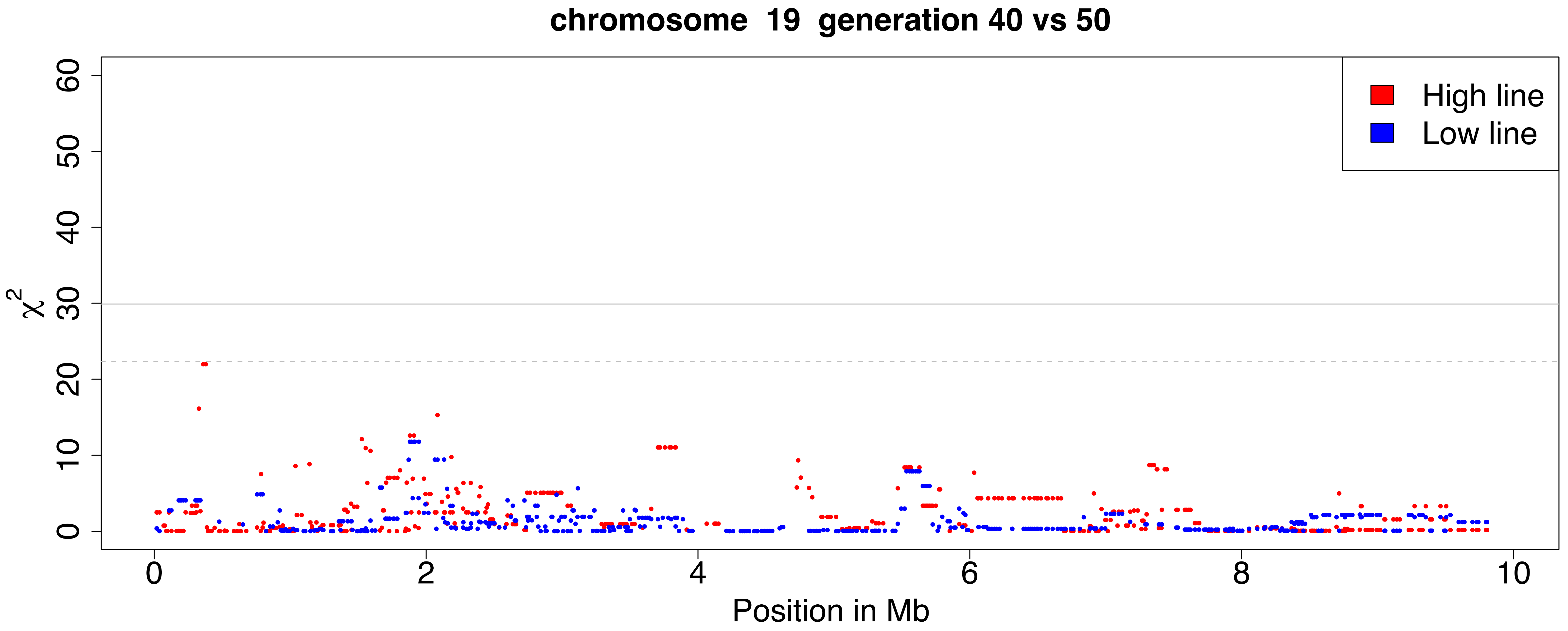
chromosome 19 generation 40 vs 50
High line
Low line
χ^2
0
10
20
30
40
50
60
0
2
4
6
8
10
Position in Mb

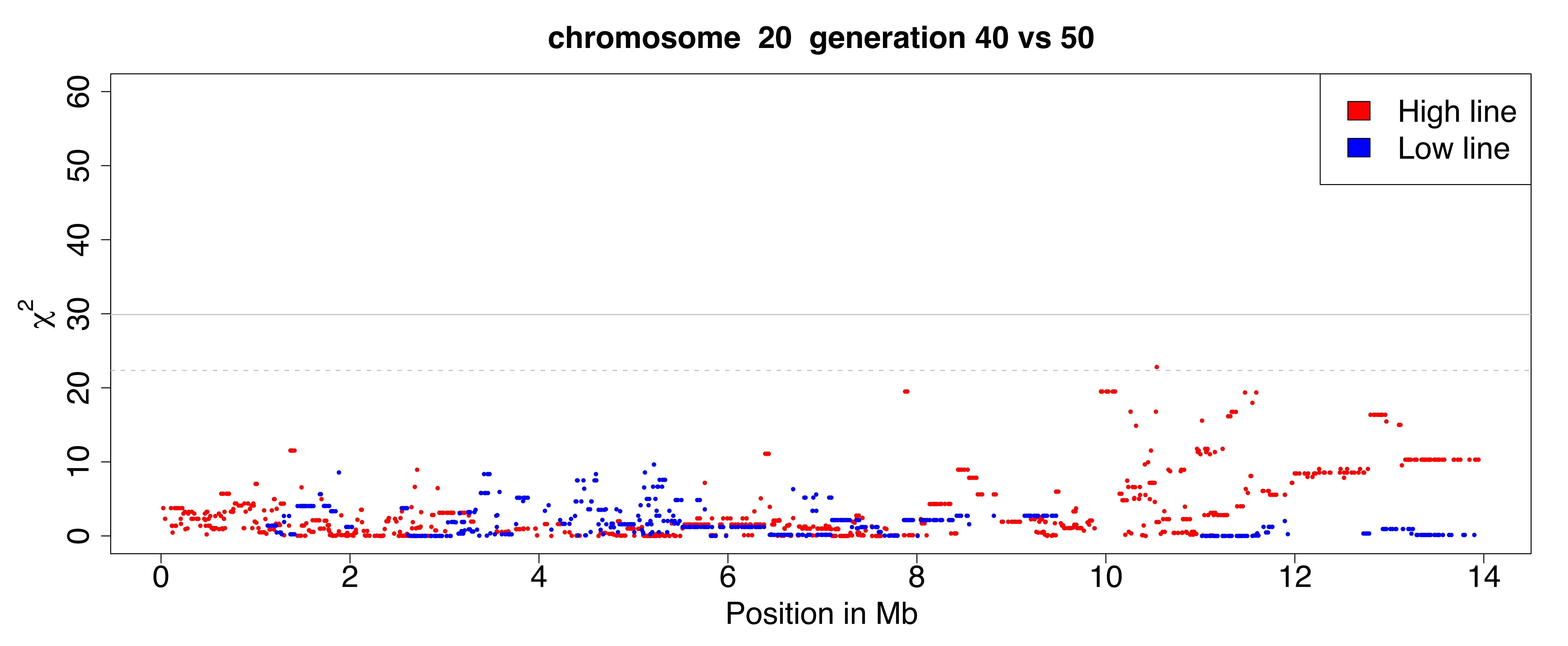

chromosome 20 generation 40 vs 50
High line
Low line
χ^2
0
10
20
30
40
50
60
0
2
4
6
8
10
12
14
Position in Mb

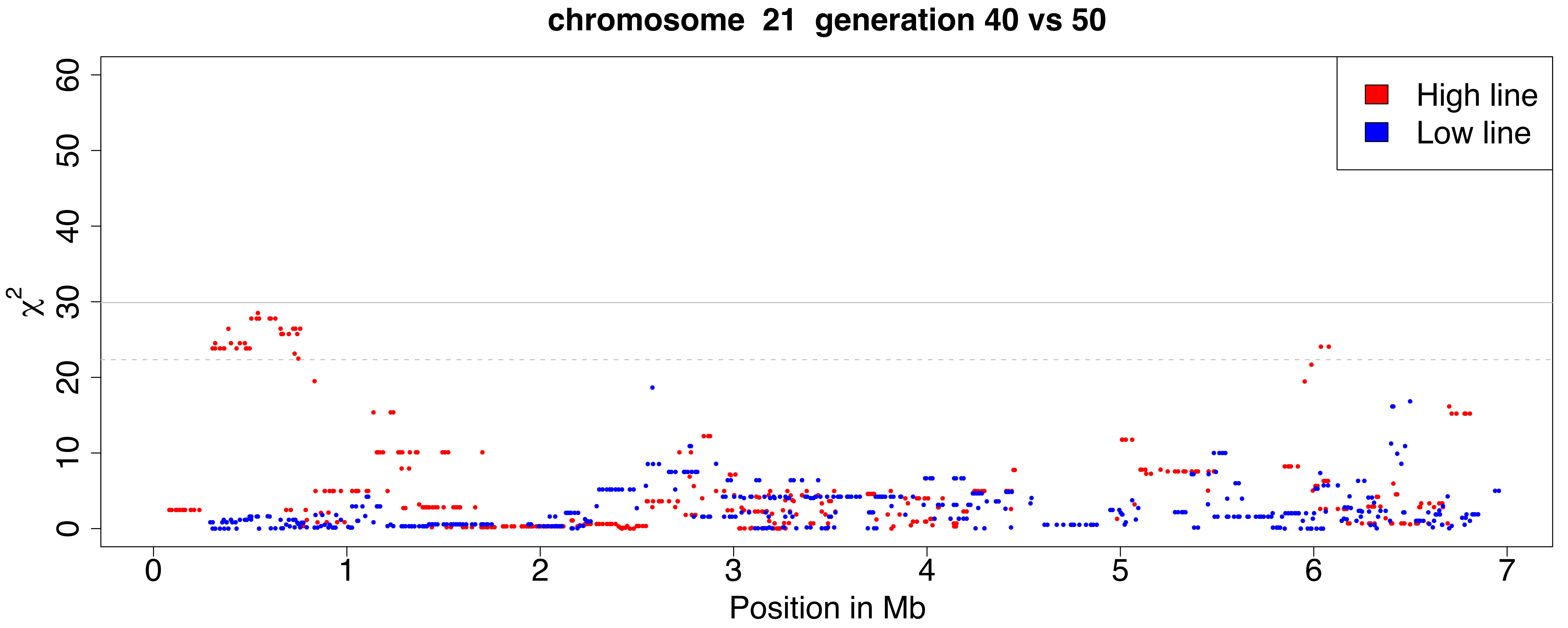
chromosome 21 generation 40 vs 50
High line
Low line
χ^2
0
10
20
30
40
50
60
0
1
2
3
4
5
6
7
Position in Mb

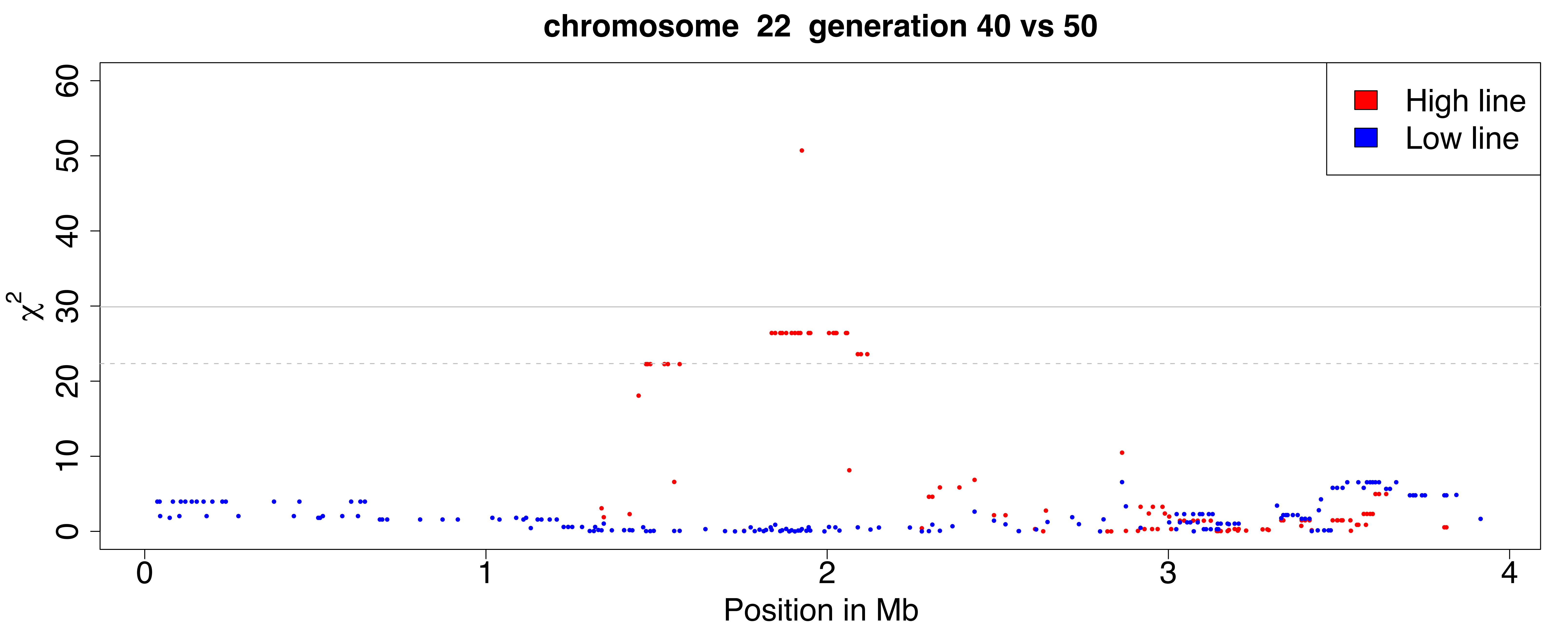

chromosome 22 generation 40 vs 50
High line
Low line
χ^2
0
10
20
30
40
50
60
Position in Mb
0
1
2
3
4

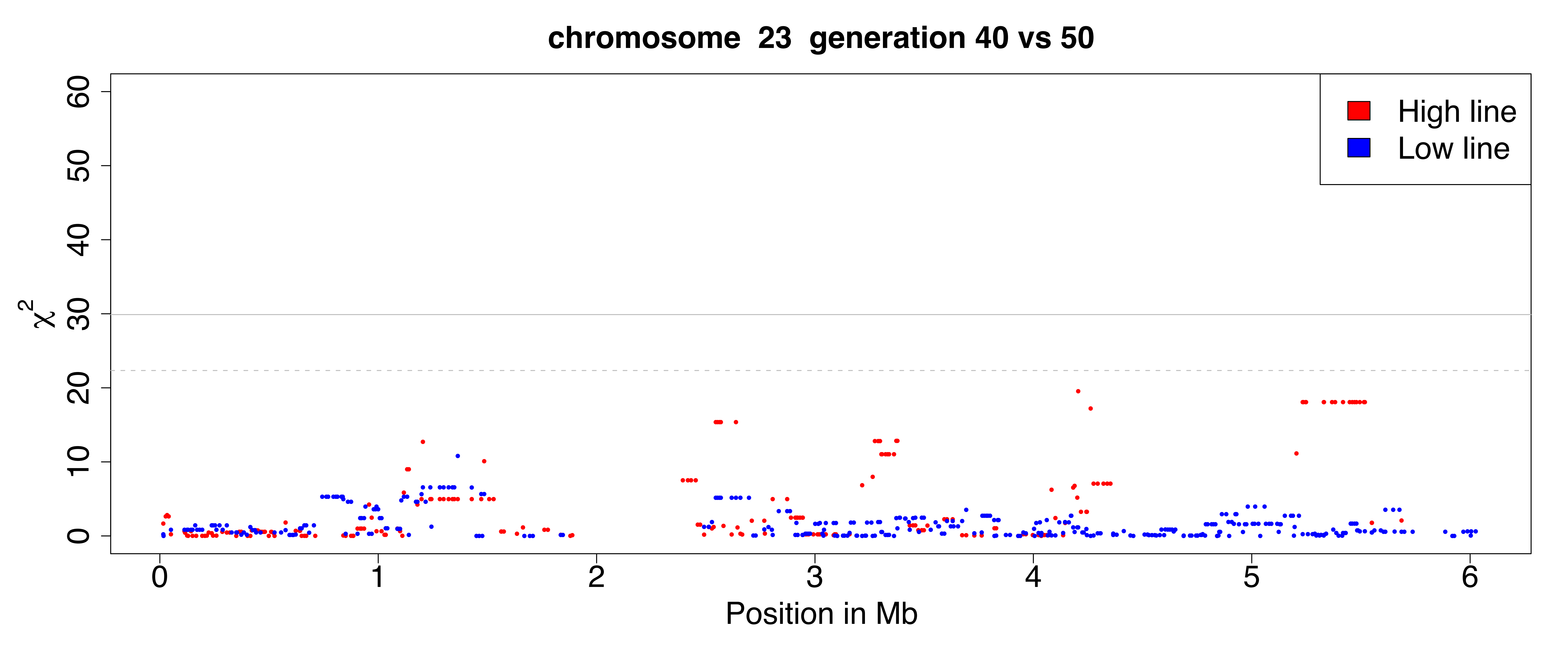
chromosome 23 generation 40 vs 50
High line
Low line
χ^2
0
10
20
30
40
50
60
0
1
2
3
4
5
6
Position in Mb

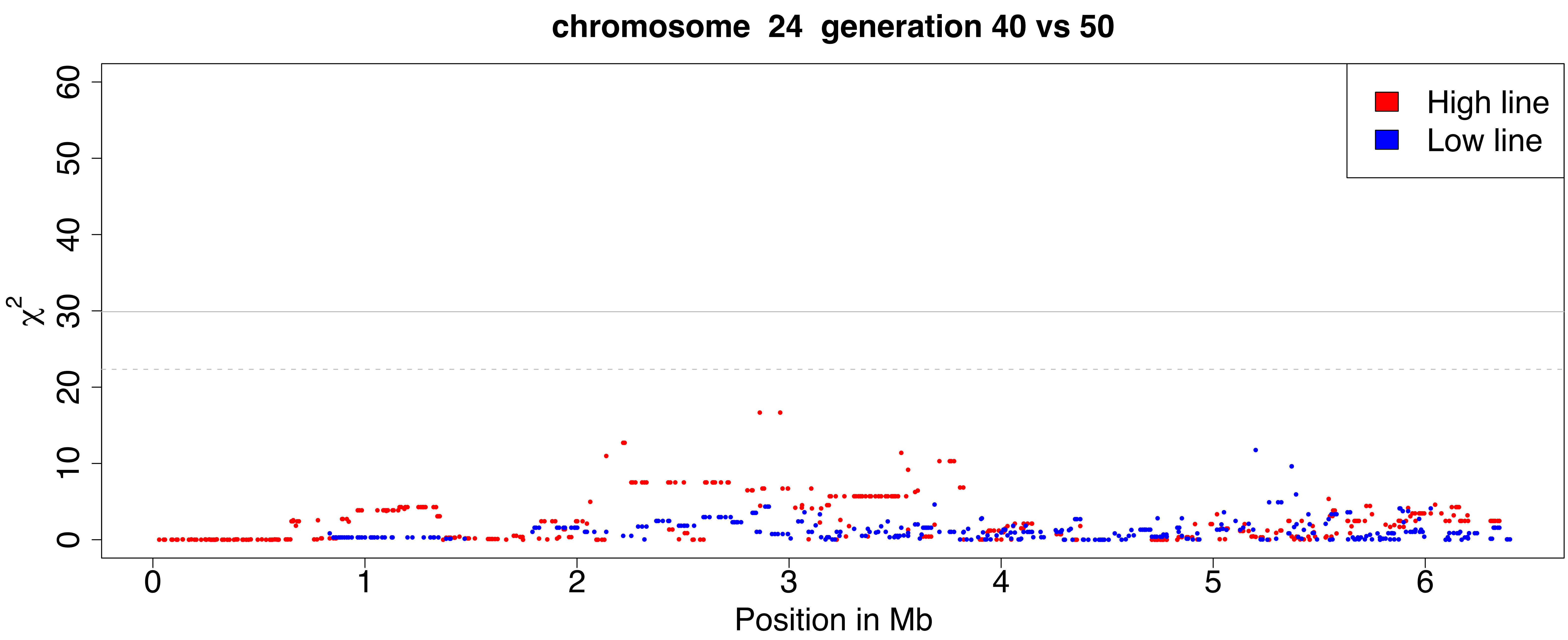
chromosome 24 generation 40 vs 50
High line
Low line
χ^2
0
10
20
30
40
50
60
0
1
2
3
4
5
6
Position in Mb

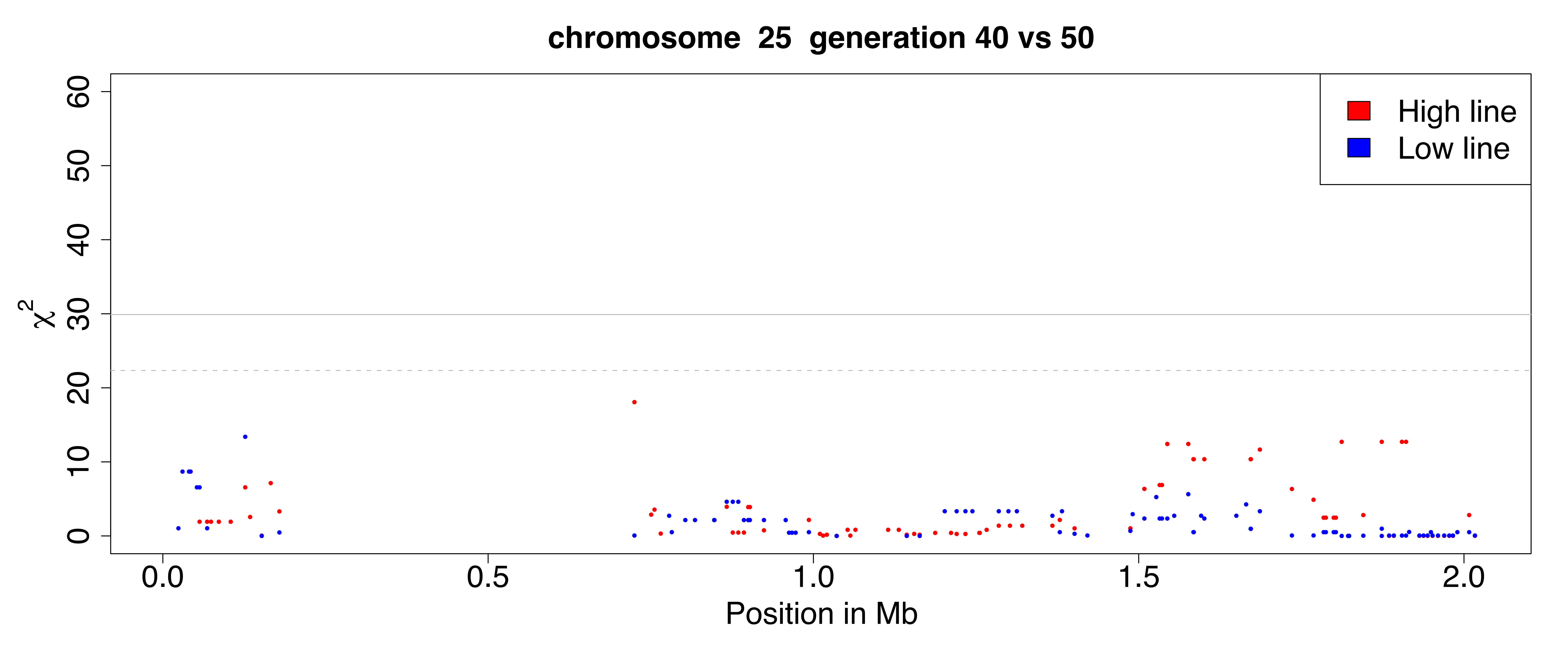

chromosome 25 generation 40 vs 50
High line
Low line
χ^2
0
10
20
30
40
50
60
0.0
0.5
1.0
1.5
2.0
Position in Mb

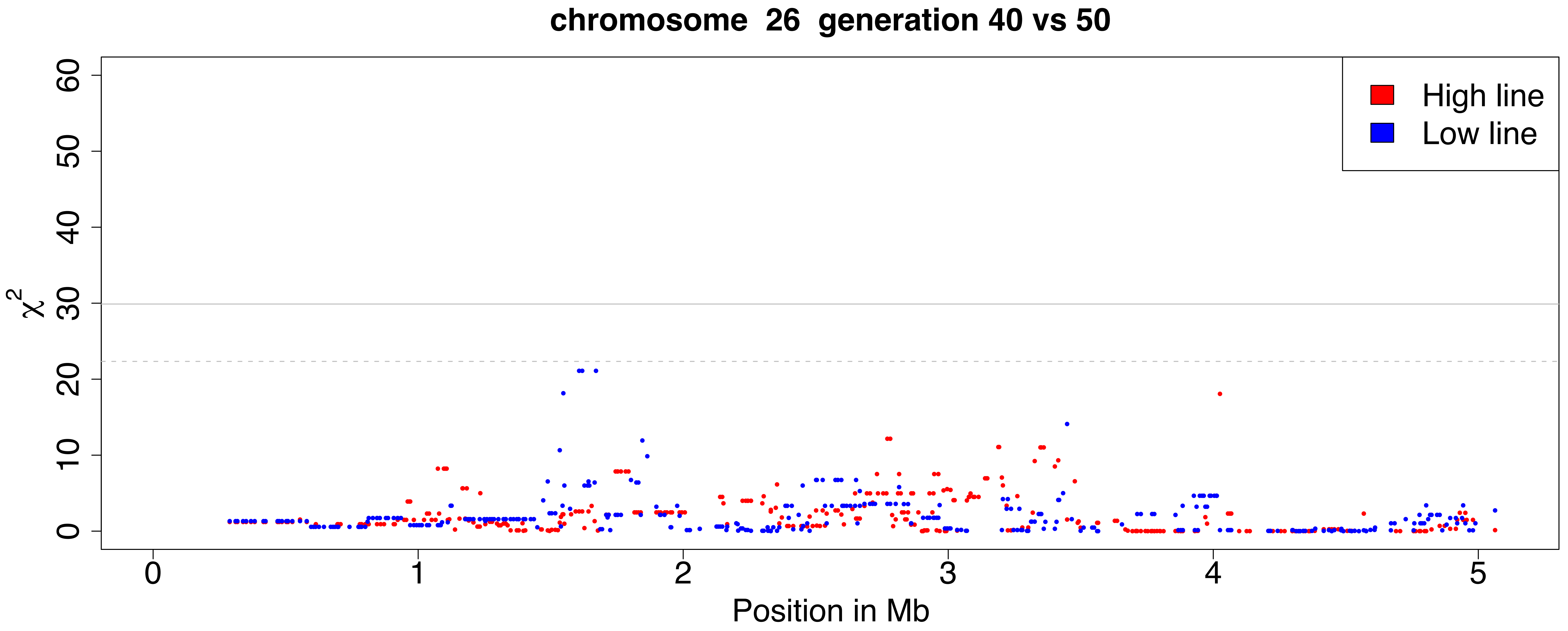

chromosome 26 generation 40 vs 50
High line
Low line
χ^2
0
10
20
30
40
50
60
0
1
2
3
4
5
Position in Mb

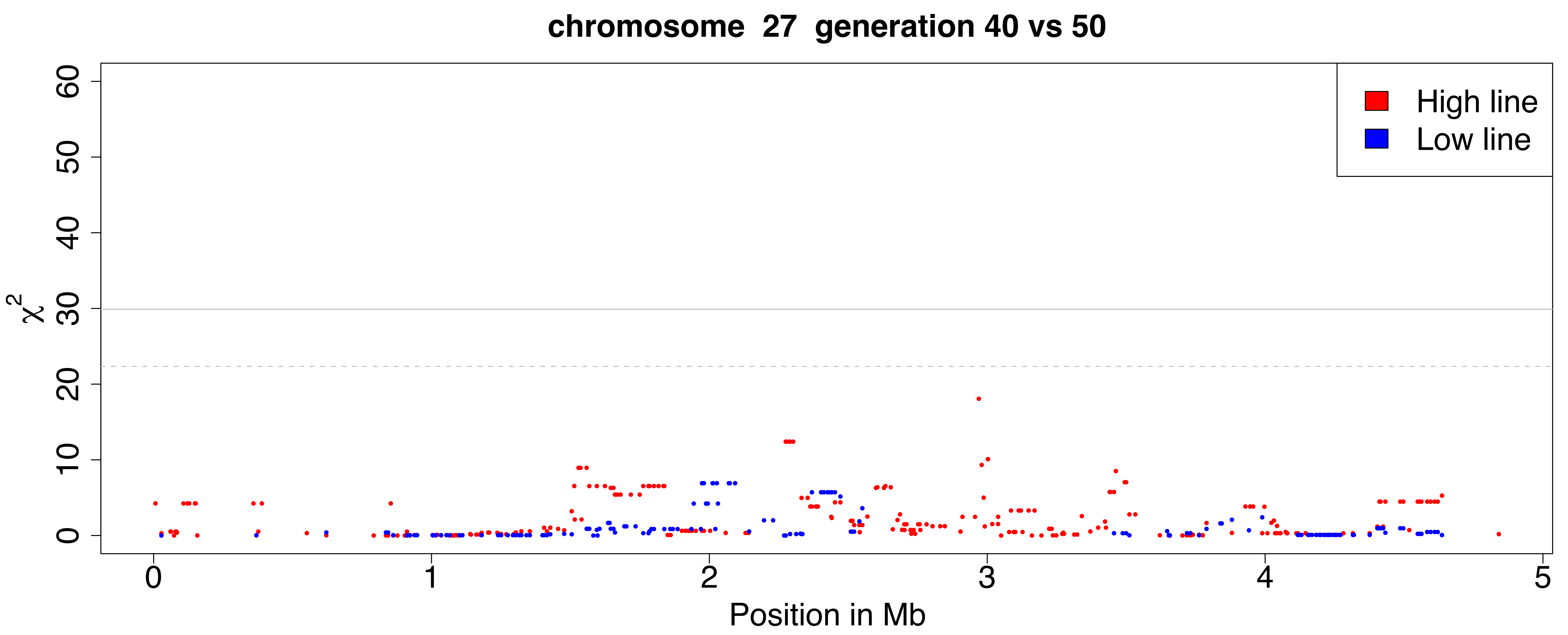

chromosome 27 generation 40 vs 50
High line
Low line
χ^2
0
10
20
30
40
50
60
0
1
2
3
4
5
Position in Mb

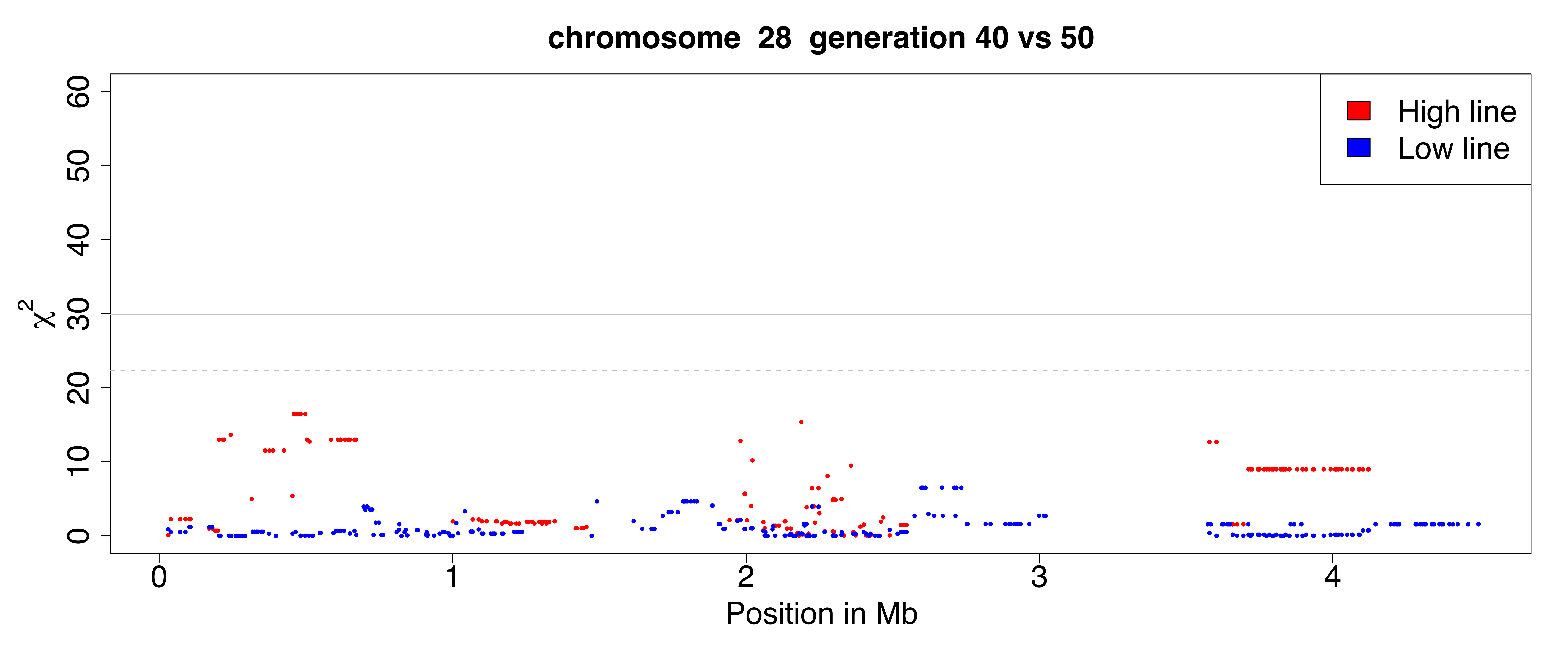
chromosome 28 generation 40 vs 50
High line
Low line
χ^2
0
10
20
30
40
50
60
0
1
2
3
4
Position in Mb

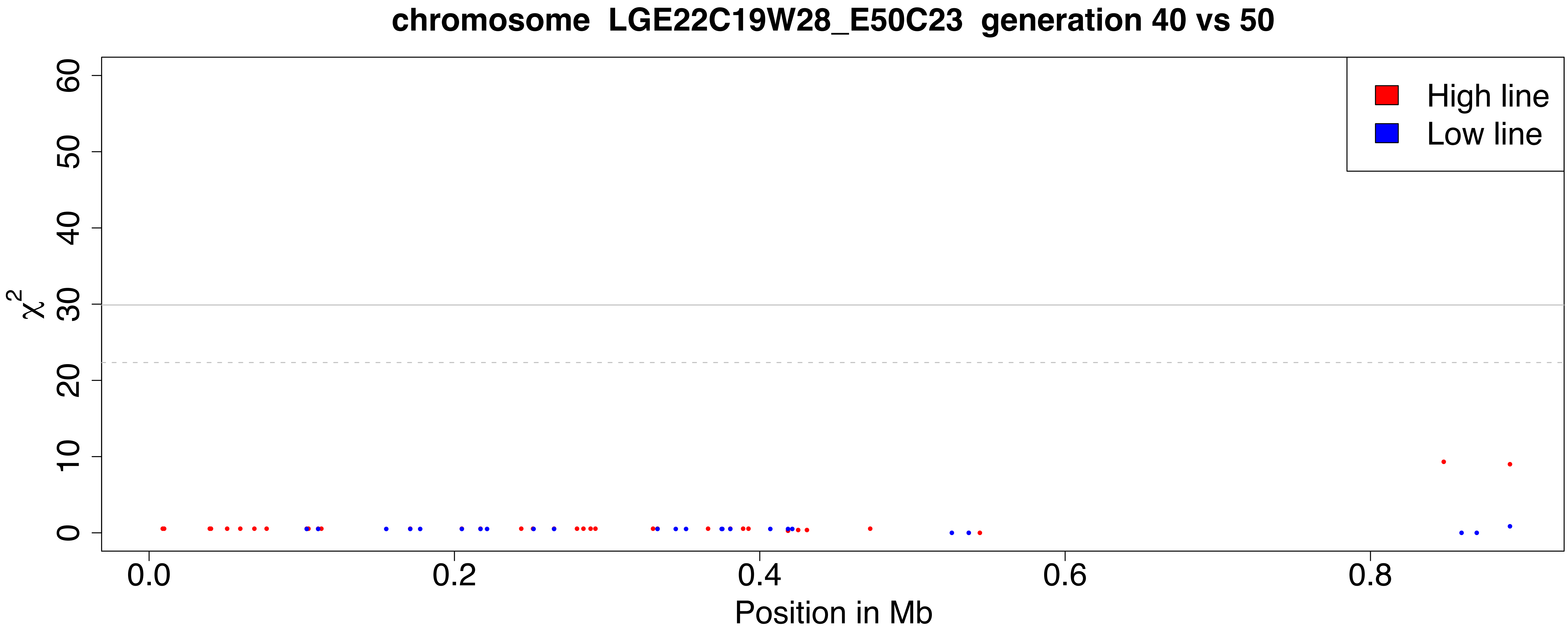

chromosome LGE22C19W28_E50C23 generation 40 vs 50
High line
Low line
χ^2
0
10
20
30
40
50
60
0.0
0.2
0.4
0.6
0.8
Position in Mb

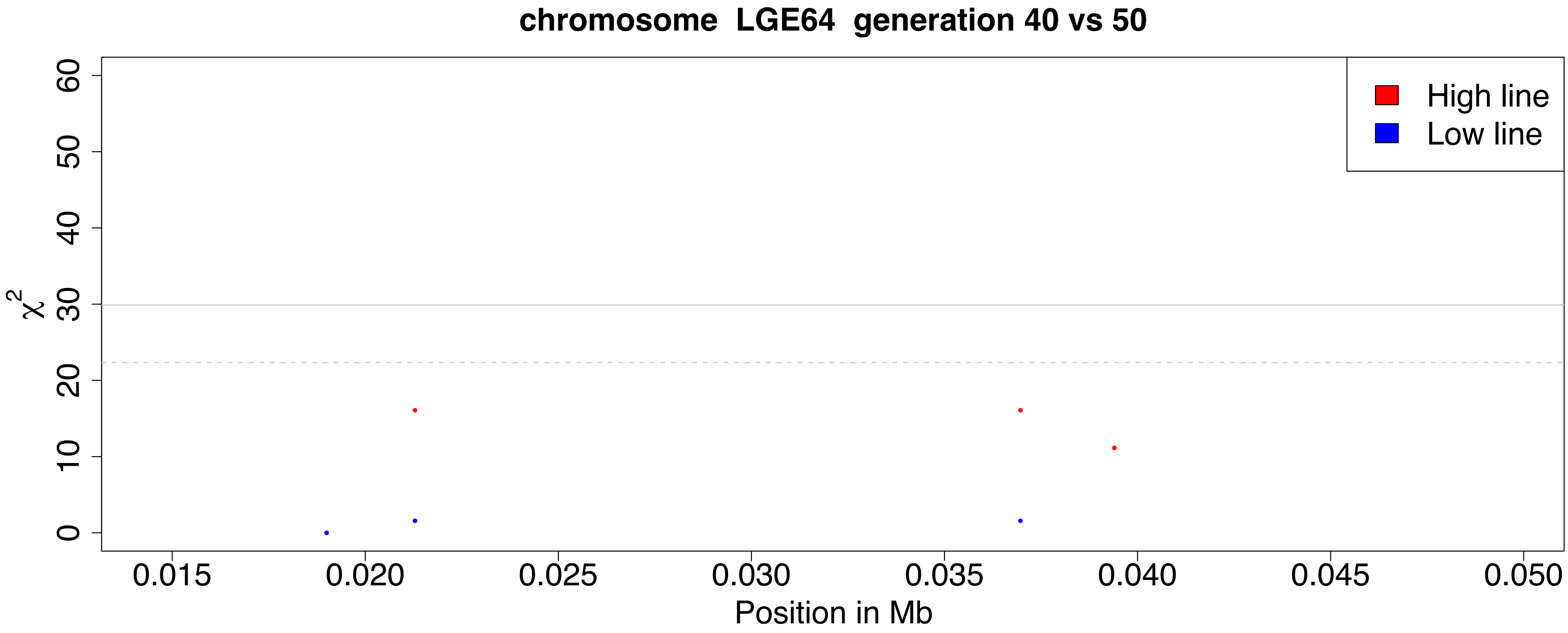

chromosome LGE64 generation 40 vs 50
High line
Low line
χ^2
0
10
20
30
40
50
60
0.015
0.020
0.025
0.030
0.035
0.040
0.045
0.050
Position in Mb

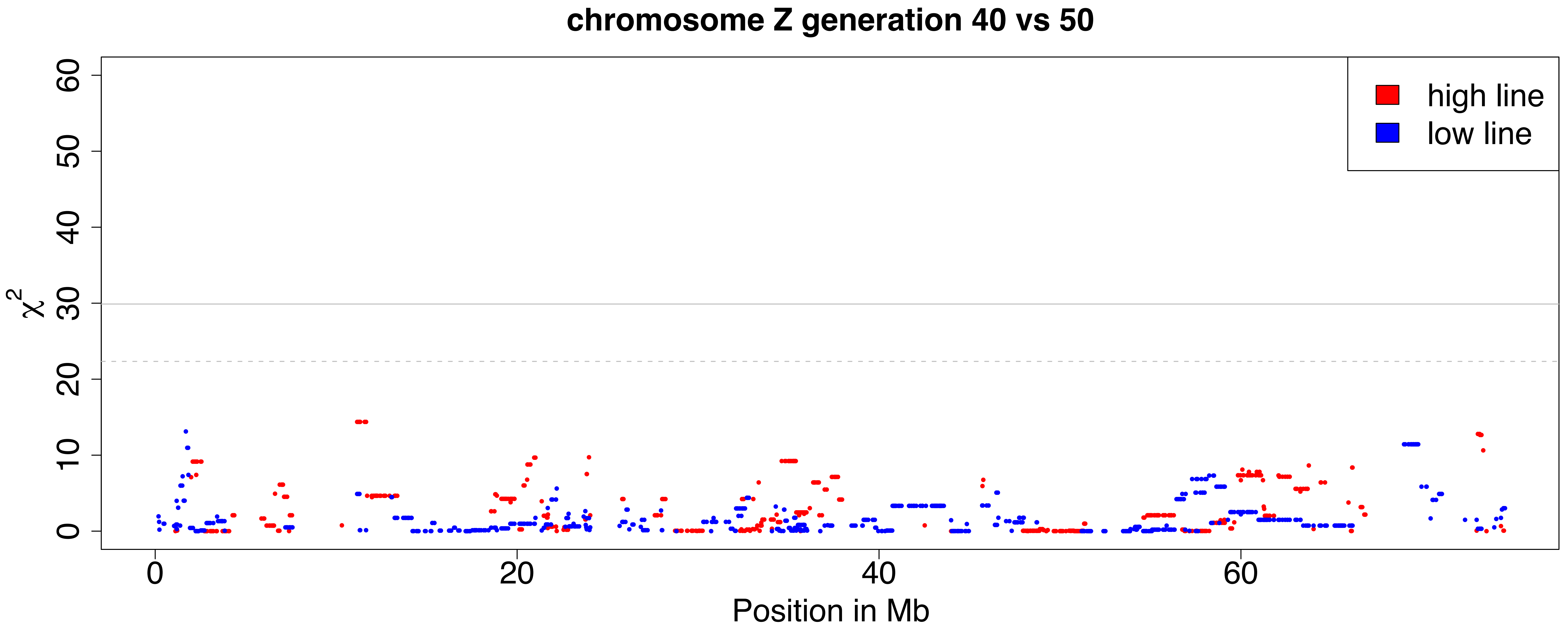

chromosome Z generation 40 vs 50
high line
low line
χ2
Position in Mb
0
10
20
30
40
50
60
0
20
40
60
