# Supplementary material for: Genome-Wide Effects of Long-Term Divergent Selection
Source: PLoS Genet. 2010 Nov 4;6(11):e1001188. doi: 10.1371/journal.pgen.1001188 (PMC2973821; doi:10.1371/journal.pgen.1001188)
Supplement: Figure S2 — Results for all chromosomes for an association test on allele frequency differences between the high- and low line. The result for individual SNPs are shown as circles and a sliding window mean of 20 markers are shown as a red line for generation 40 and as a purple line for generation 50. The dashed lines indicate the maximum χ2 values, which is obtained when a SNP is fixed for different alleles in the high and low line (80 and 118, respectively). The grey line indicates the Bonferroni corrected significance level at p<0.001. (0.97 MB PDF) [file pgen.1001188.s002.pdf]

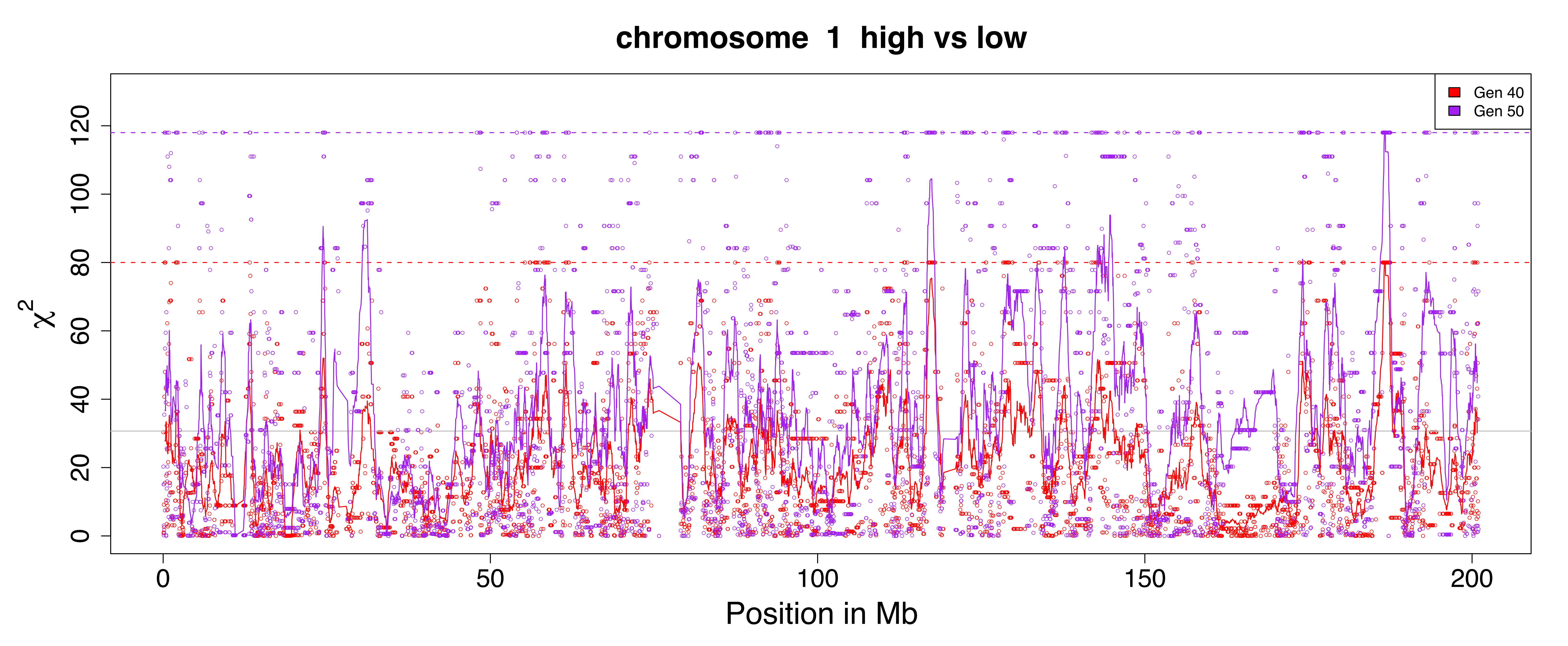

chromosome 1 high vs low
Gen 40
Gen 50
χ2
0
20
40
60
80
100
120
0
50
100
150
200
Position in Mb

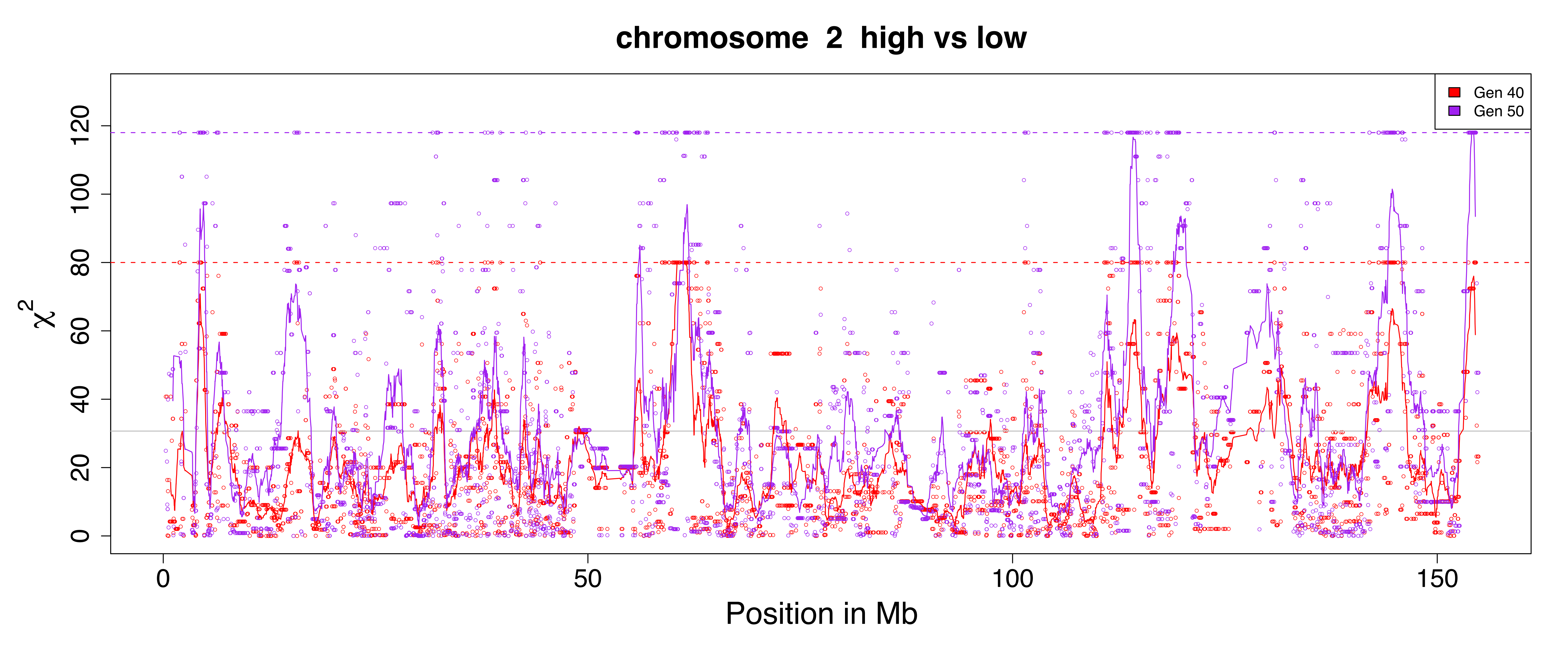
chromosome 2 high vs low
Gen 40
Gen 50
χ^2
0
20
40
60
80
100
120
0
50
100
150
Position in Mb

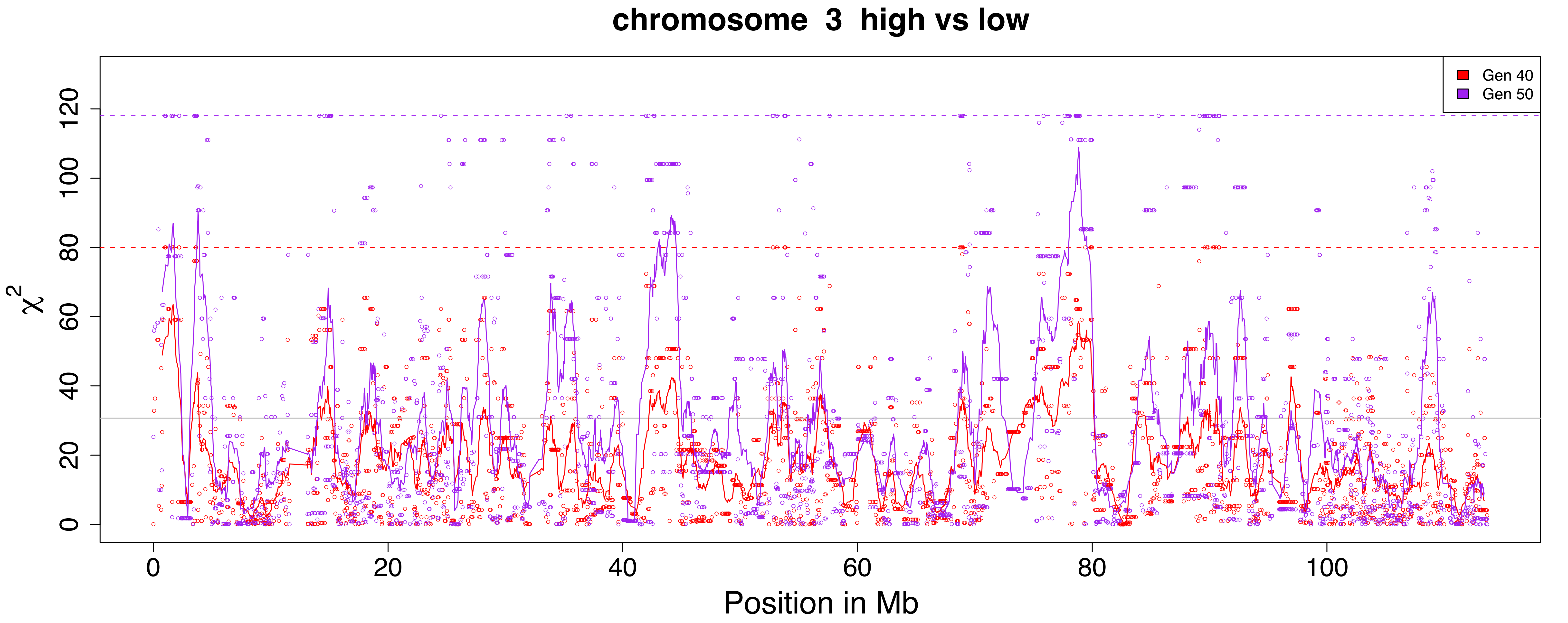
chromosome 3 high vs low
Gen 40
Gen 50
χ^2
0
20
40
60
80
100
120
Position in Mb

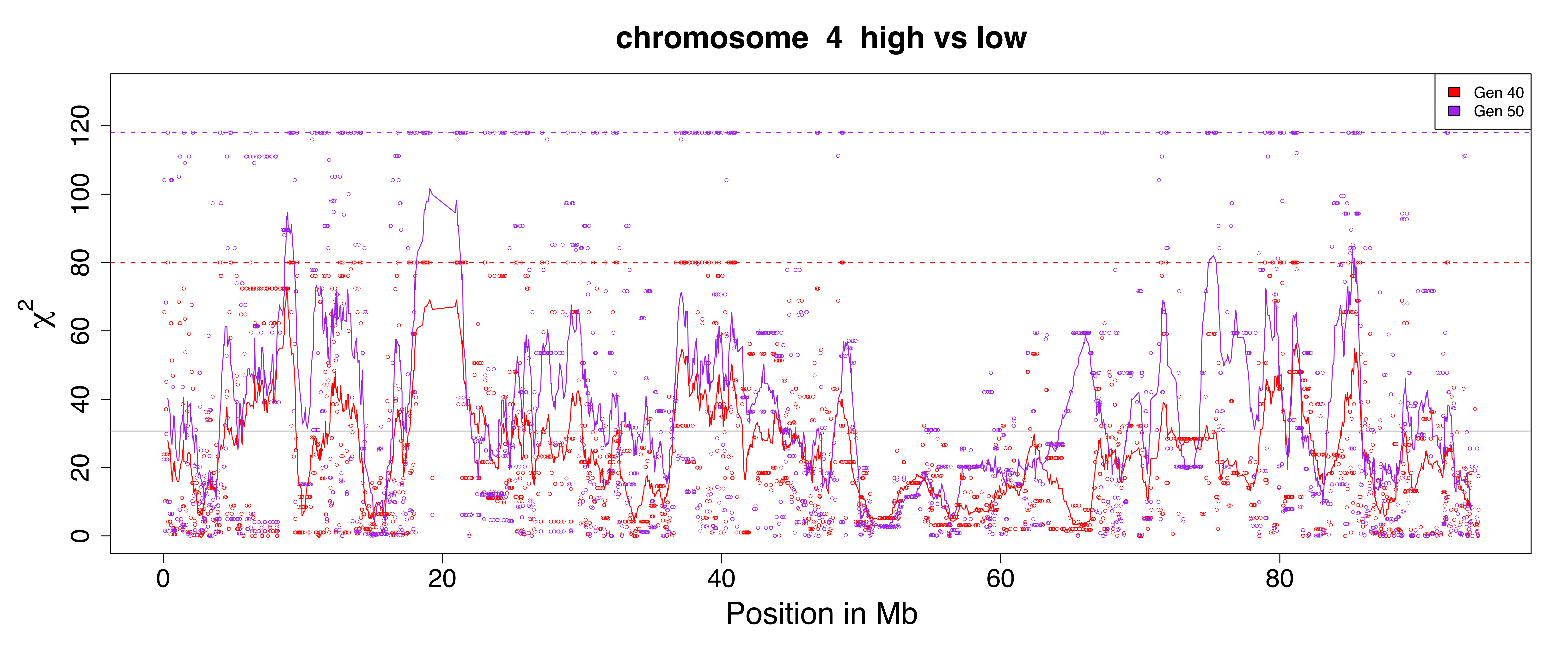
chromosome 4 high vs low
Gen 40
Gen 50
χ^2
0
20
40
60
80
100
120
0
20
40
60
80
Position in Mb

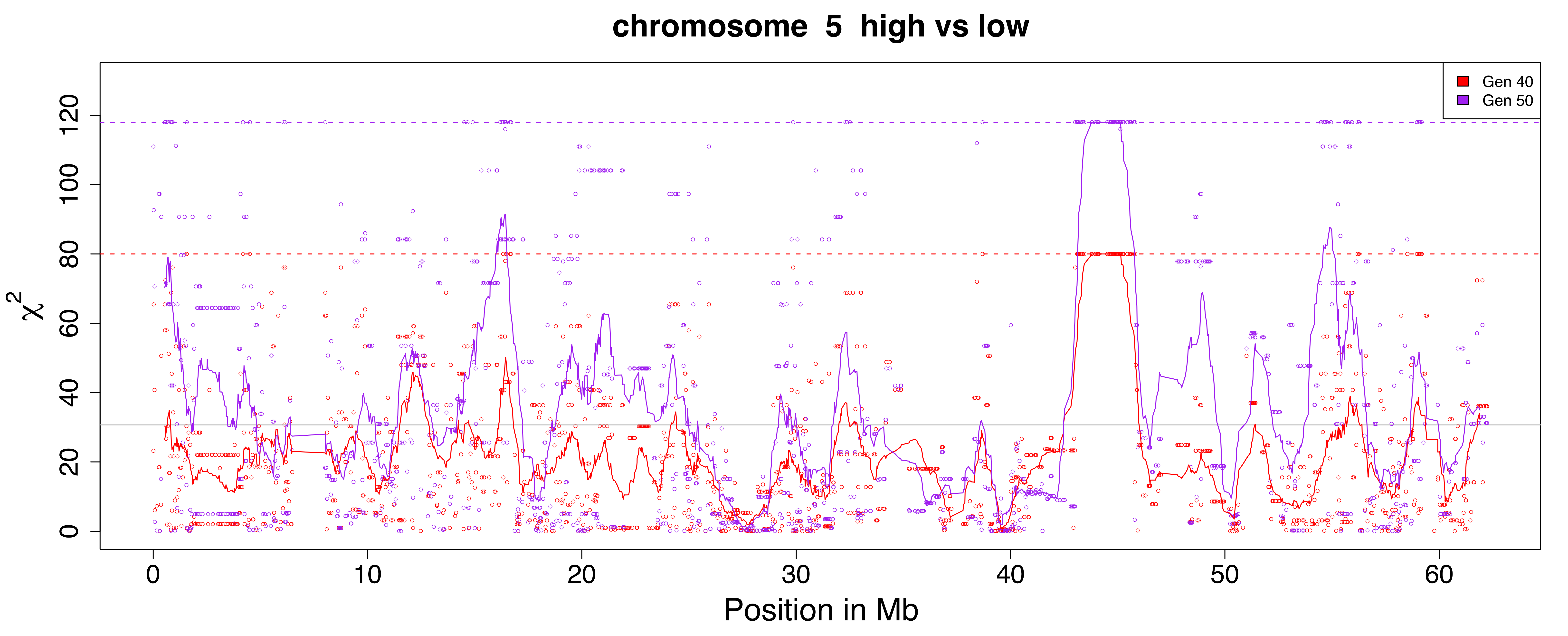

chromosome 5 high vs low
Gen 40
Gen 50
χ^2
0
20
40
60
80
100
120
0
10
20
30
40
50
60
Position in Mb

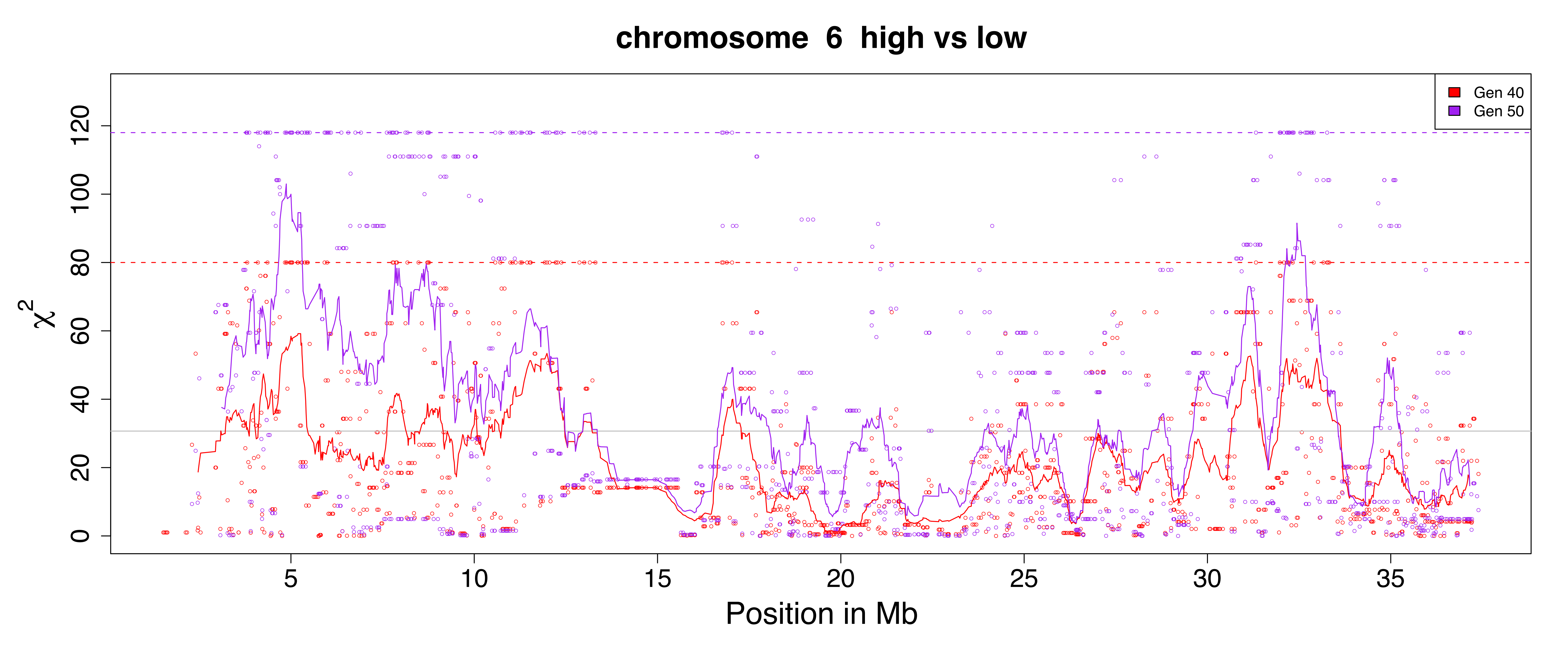
chromosome 6 high vs low
Gen 40
Gen 50
χ^2
0
20
40
60
80
100
120
5
10
15
20
25
30
35
Position in Mb

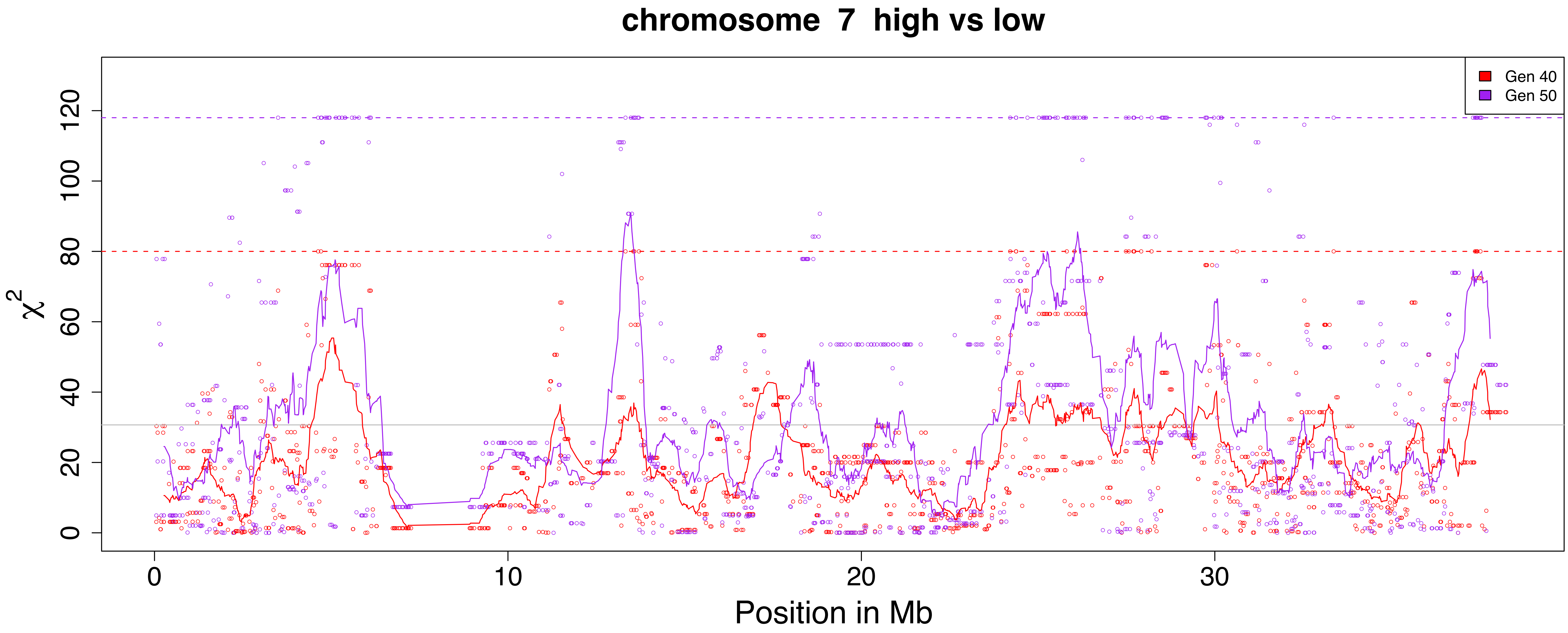

chromosome 7 high vs low
Gen 40
Gen 50
χ^2
0
20
40
60
80
100
120
0
10
20
30
Position in Mb

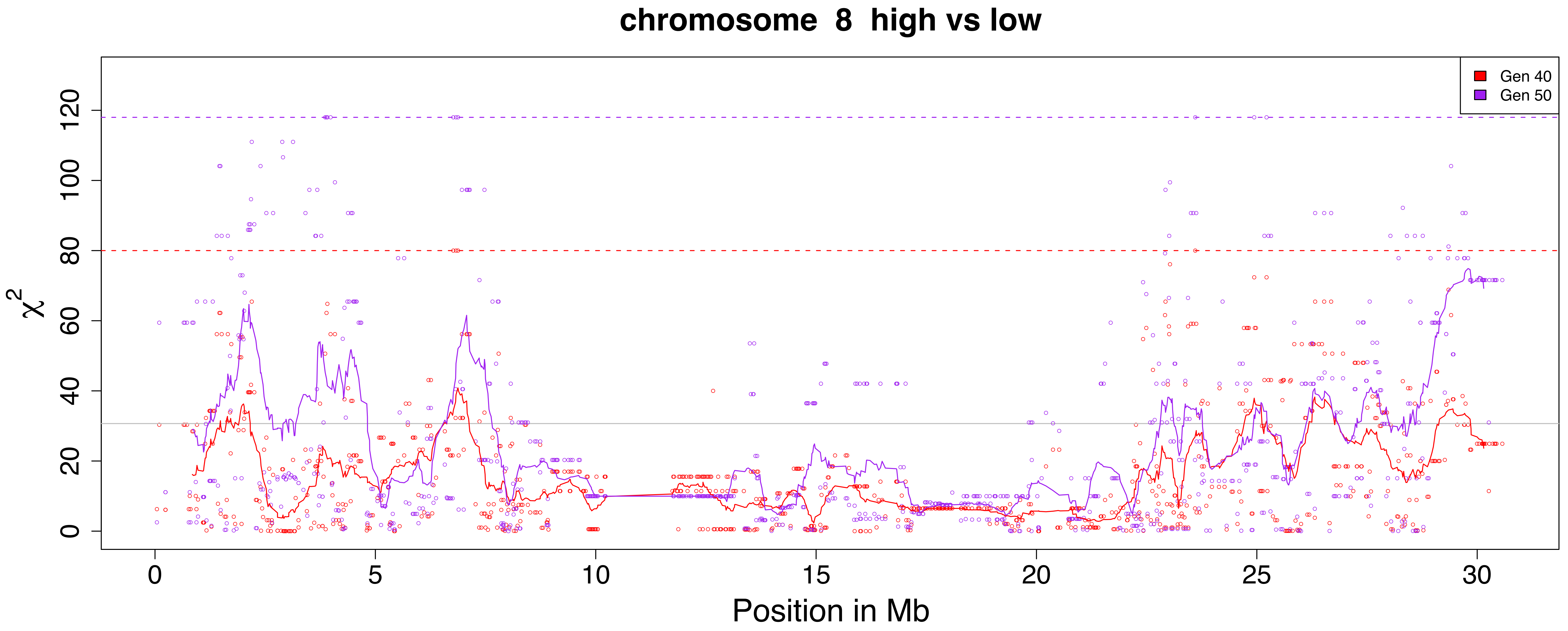

chromosome 8 high vs low
Gen 40
Gen 50
χ^2
0
20
40
60
80
100
120
0
5
10
15
20
25
30
Position in Mb

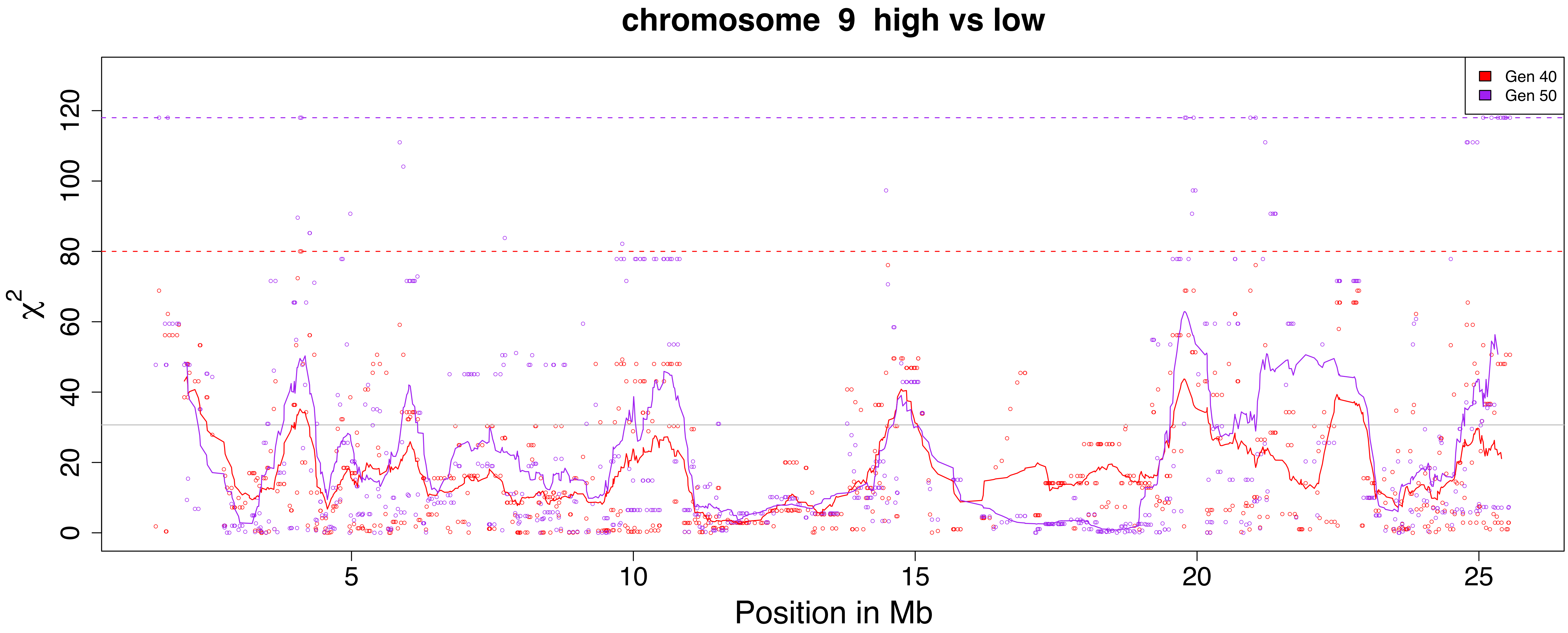
chromosome 9 high vs low
Gen 40
Gen 50
χ^2
0
20
40
60
80
100
120
5
10
15
20
25
Position in Mb

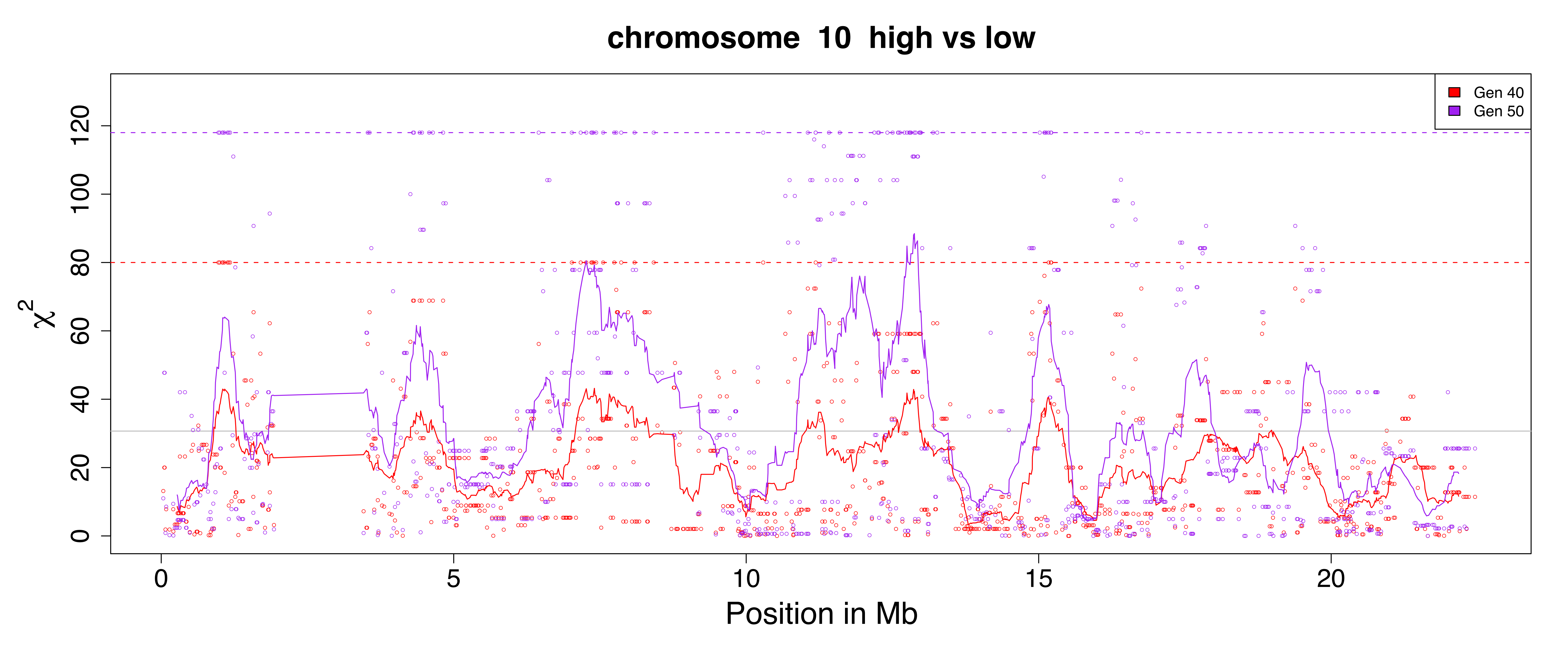

chromosome 10 high vs low
Gen 40
Gen 50
χ2
0
20
40
60
80
100
120
0
5
10
15
20
Position in Mb

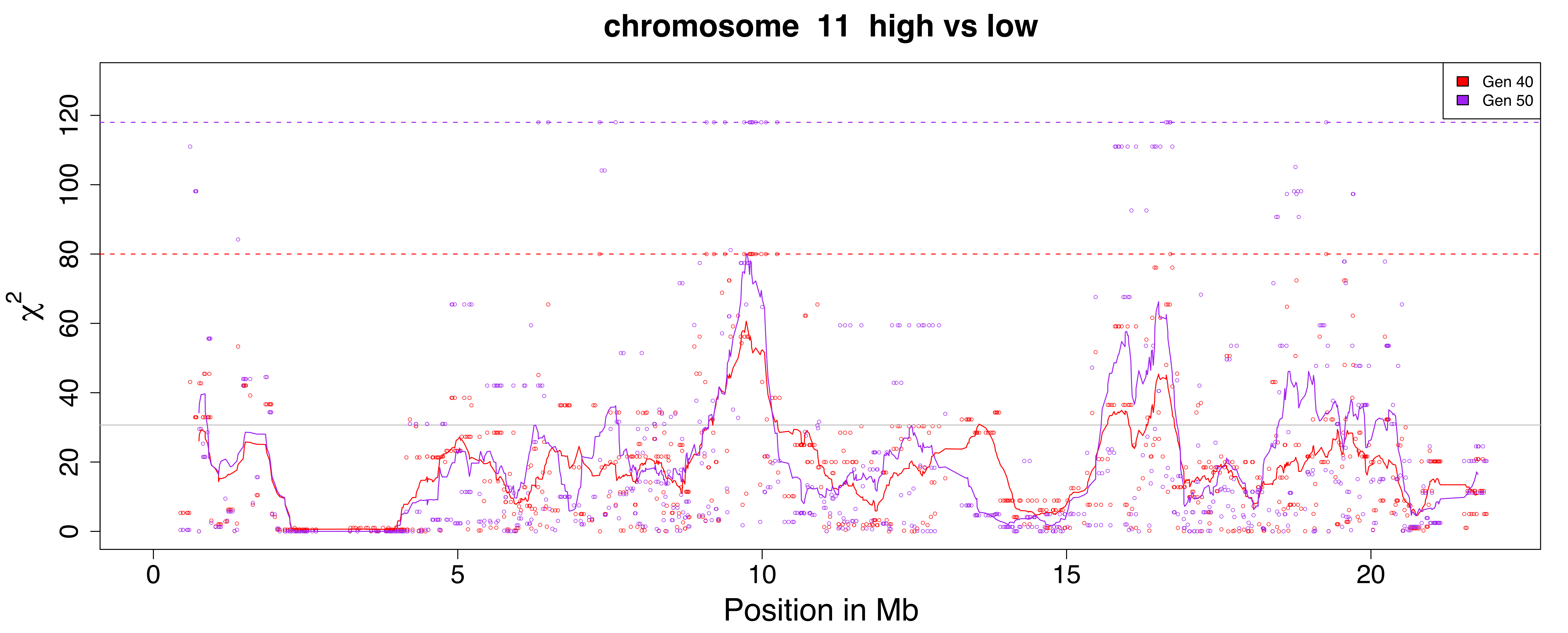

chromosome 11 high vs low
Gen 40
Gen 50
χ^2
0
20
40
60
80
100
120
0
5
10
15
20
Position in Mb

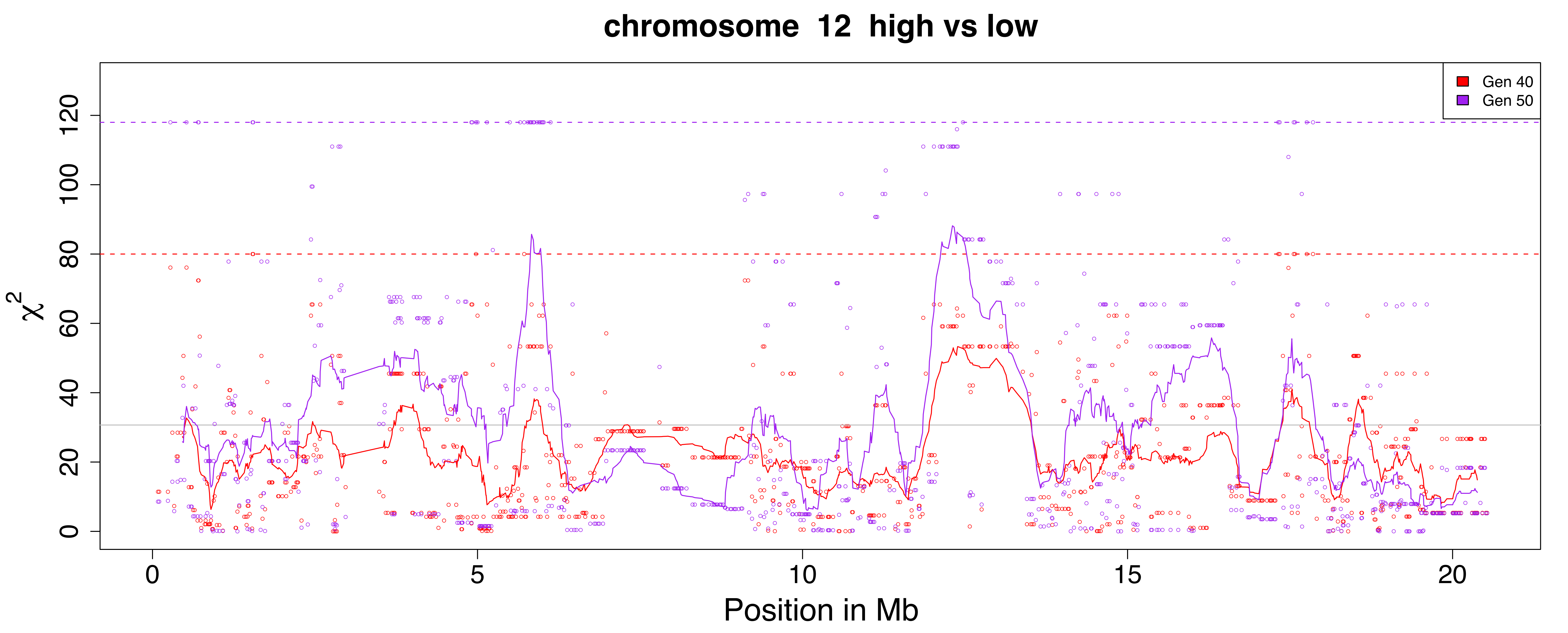

chromosome 12 high vs low
Gen 40
Gen 50
χ^2
0
20
40
60
80
100
120
0
5
10
15
20
Position in Mb

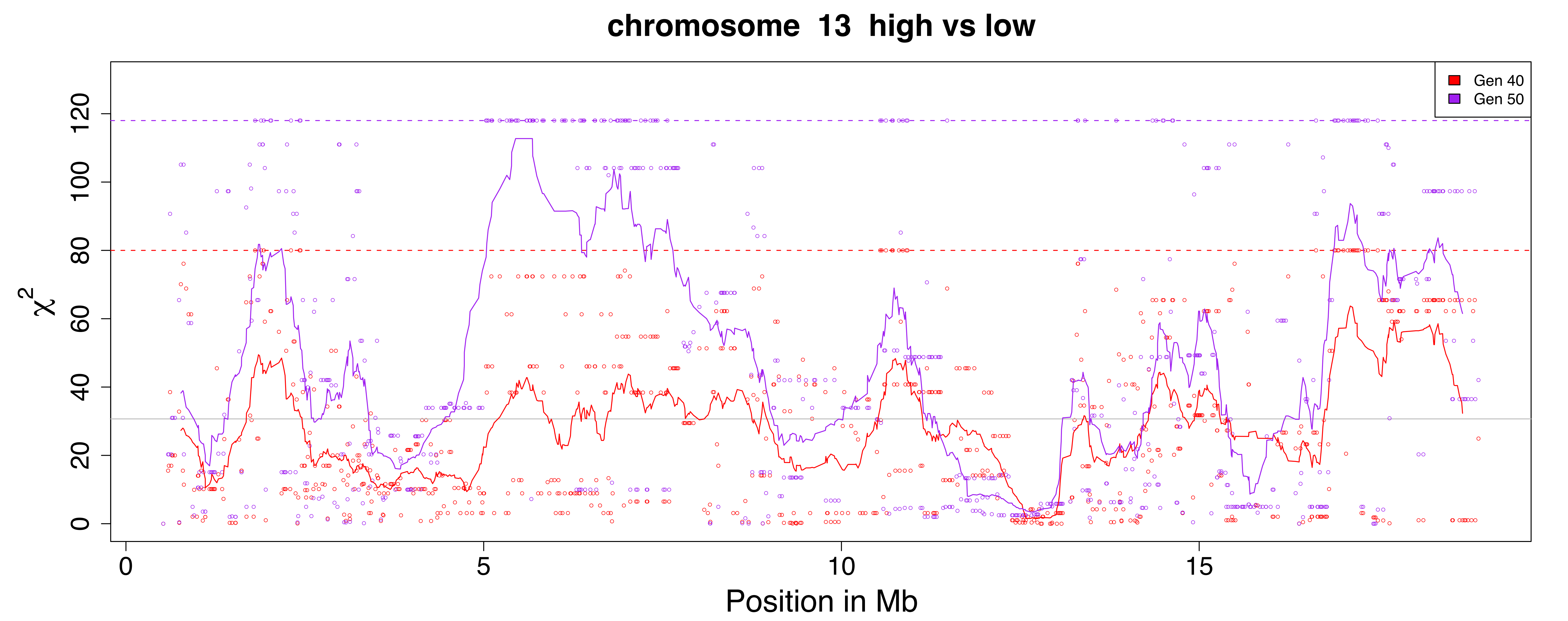

chromosome 13 high vs low
Gen 40
Gen 50
χ^2
0
20
40
60
80
100
120
0
5
10
15
Position in Mb

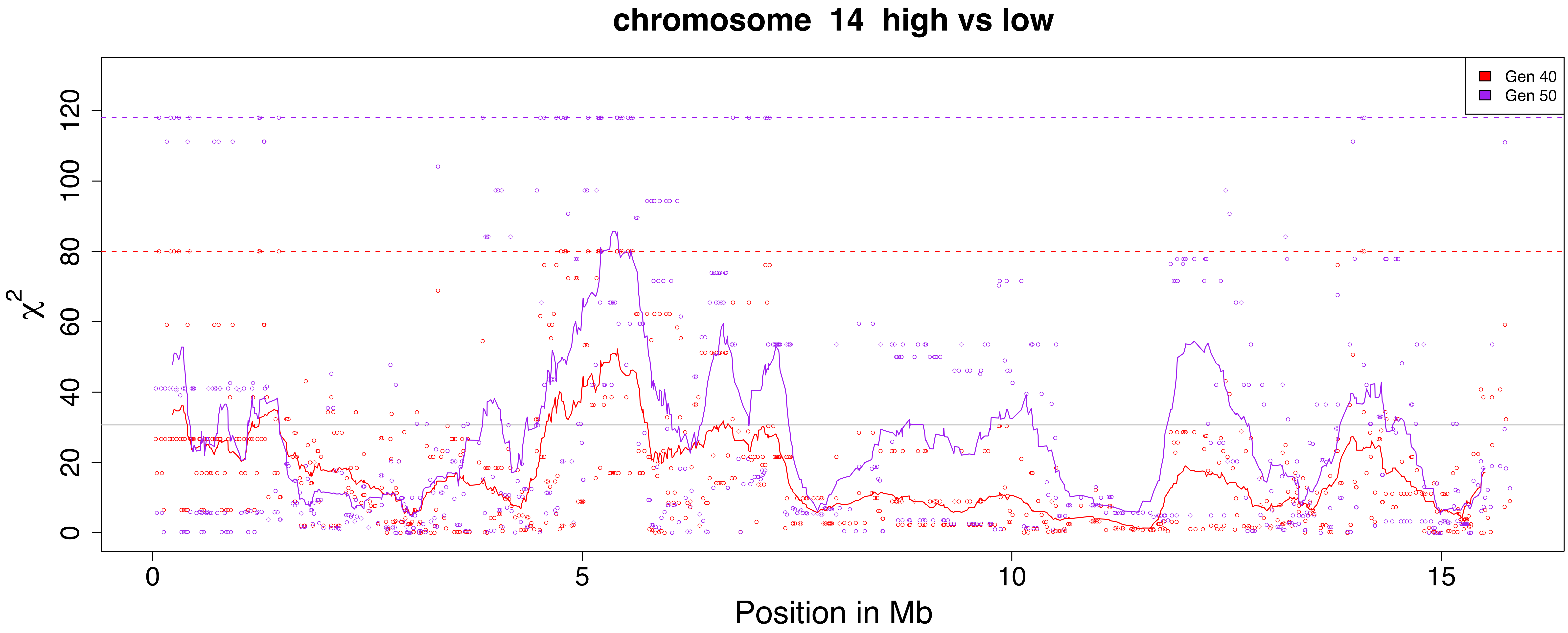
chromosome 14 high vs low
Gen 40
Gen 50
χ^2
0
20
40
60
80
100
120
0
5
10
15
Position in Mb

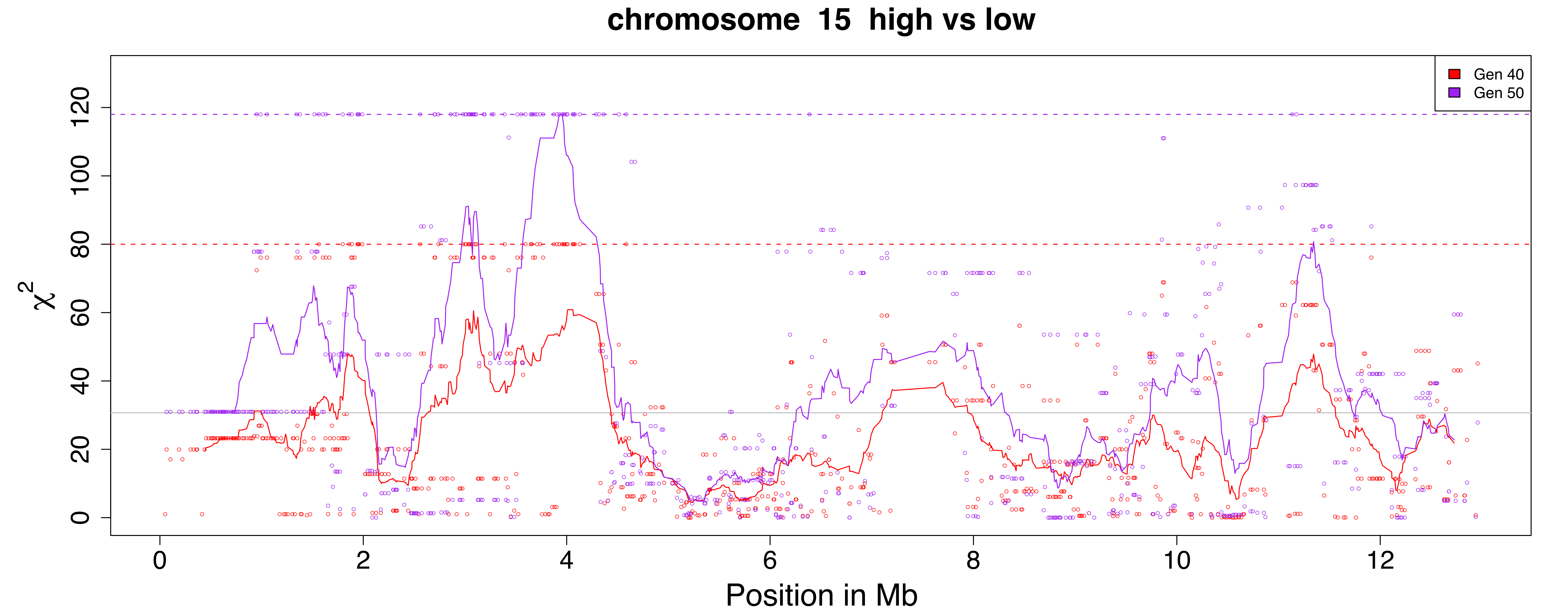

chromosome 15 high vs low
Gen 40
Gen 50
χ^2
0
20
40
60
80
100
120
0
2
4
6
8
10
12
Position in Mb

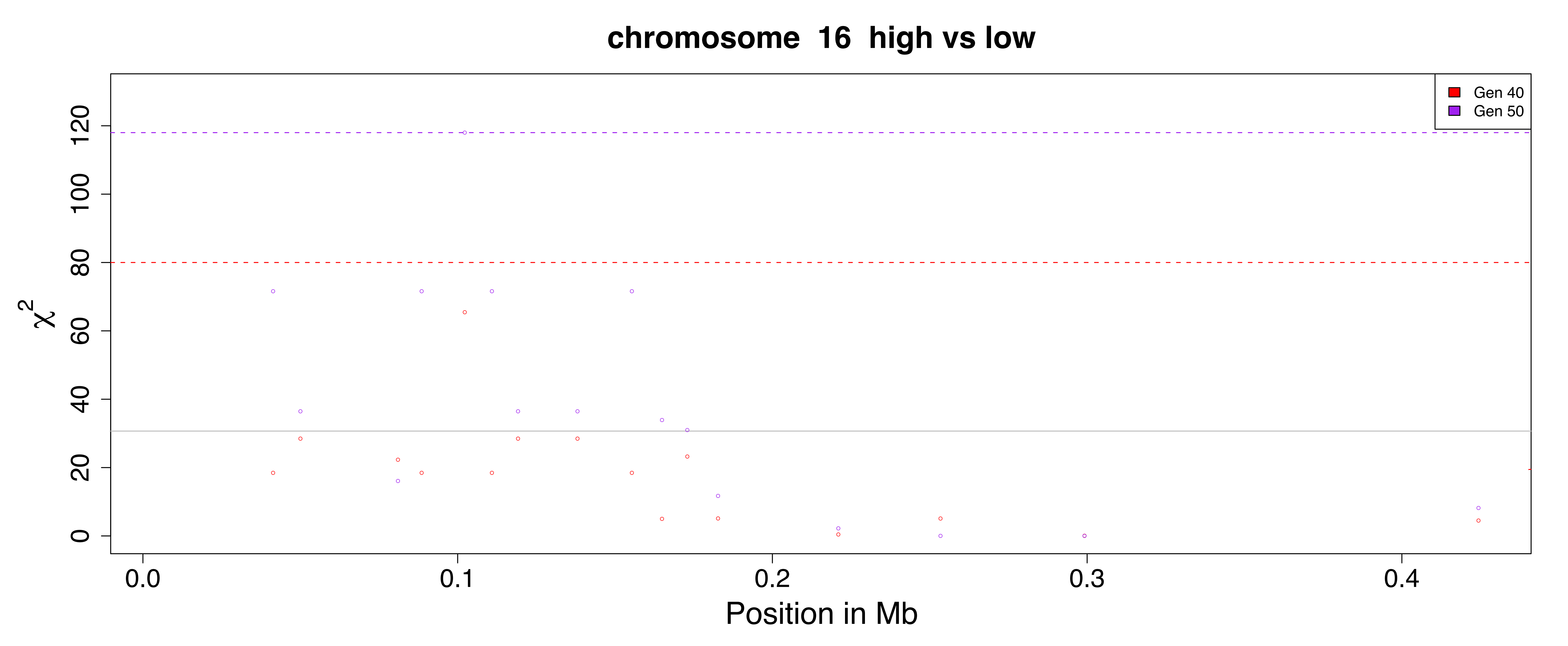

chromosome 16 high vs low
Gen 40
Gen 50
χ2
Position in Mb
0
20
40
60
80
100
120
0.0
0.1
0.2
0.3
0.4

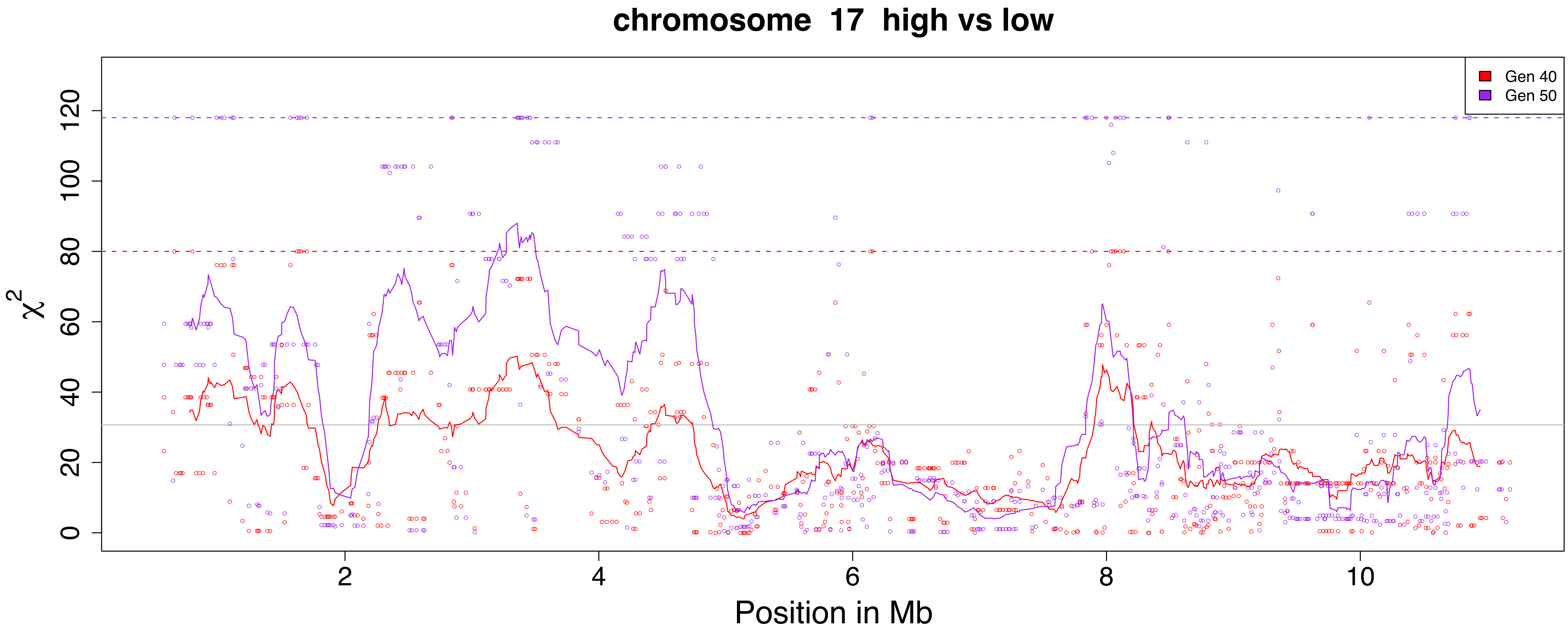
chromosome 17 high vs low
Gen 40
Gen 50
χ^2
0
20
40
60
80
100
120
2
4
6
8
10
Position in Mb

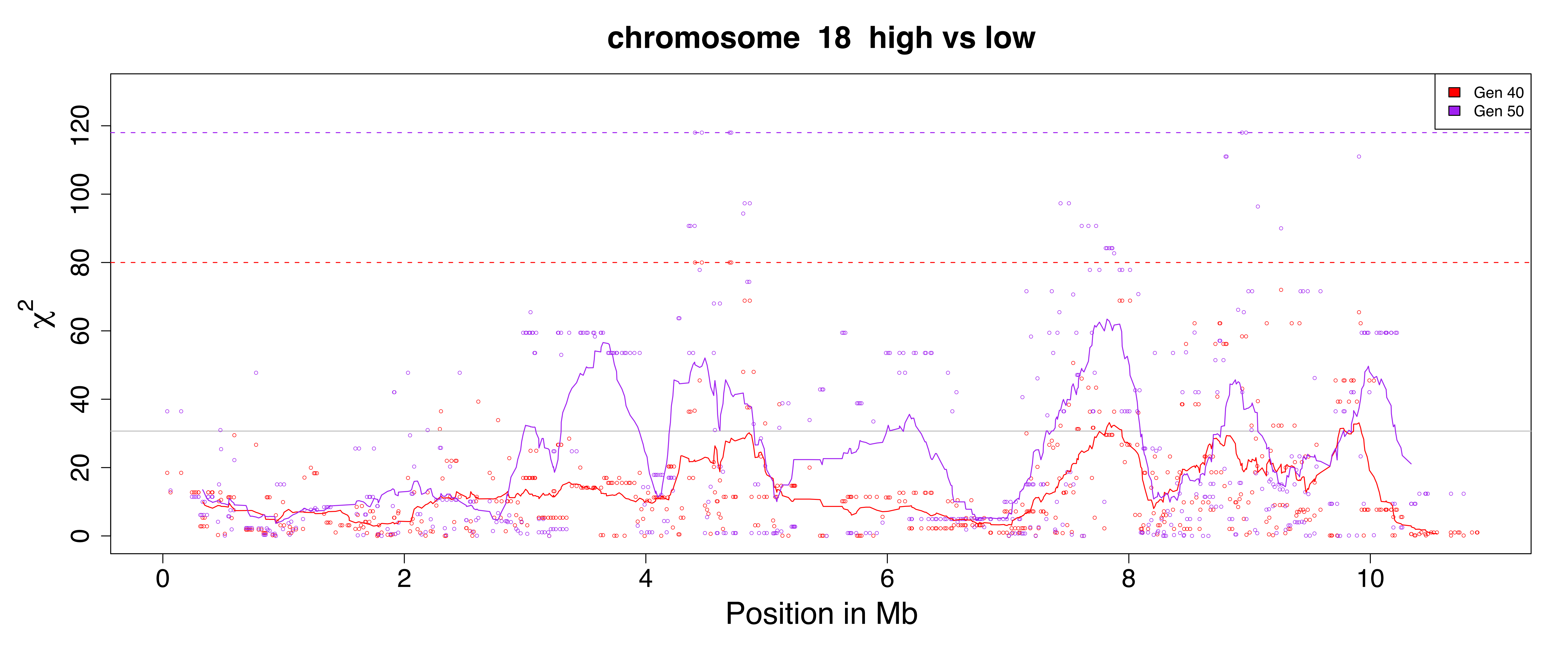

chromosome 18 high vs low
Gen 40
Gen 50
χ^2
0
20
40
60
80
100
120
0
2
4
6
8
10
Position in Mb

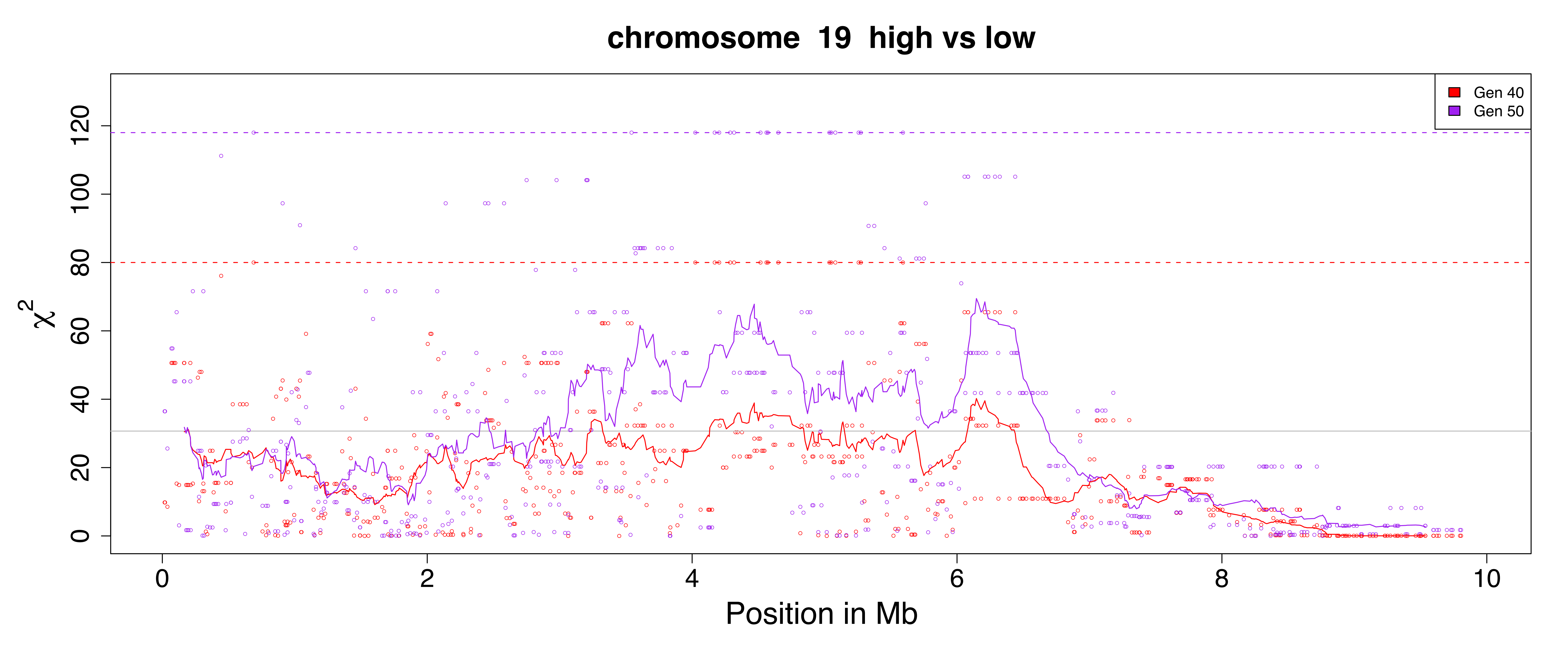

chromosome 19 high vs low
Gen 40
Gen 50
χ^2
0
20
40
60
80
100
120
0
2
4
6
8
10
Position in Mb

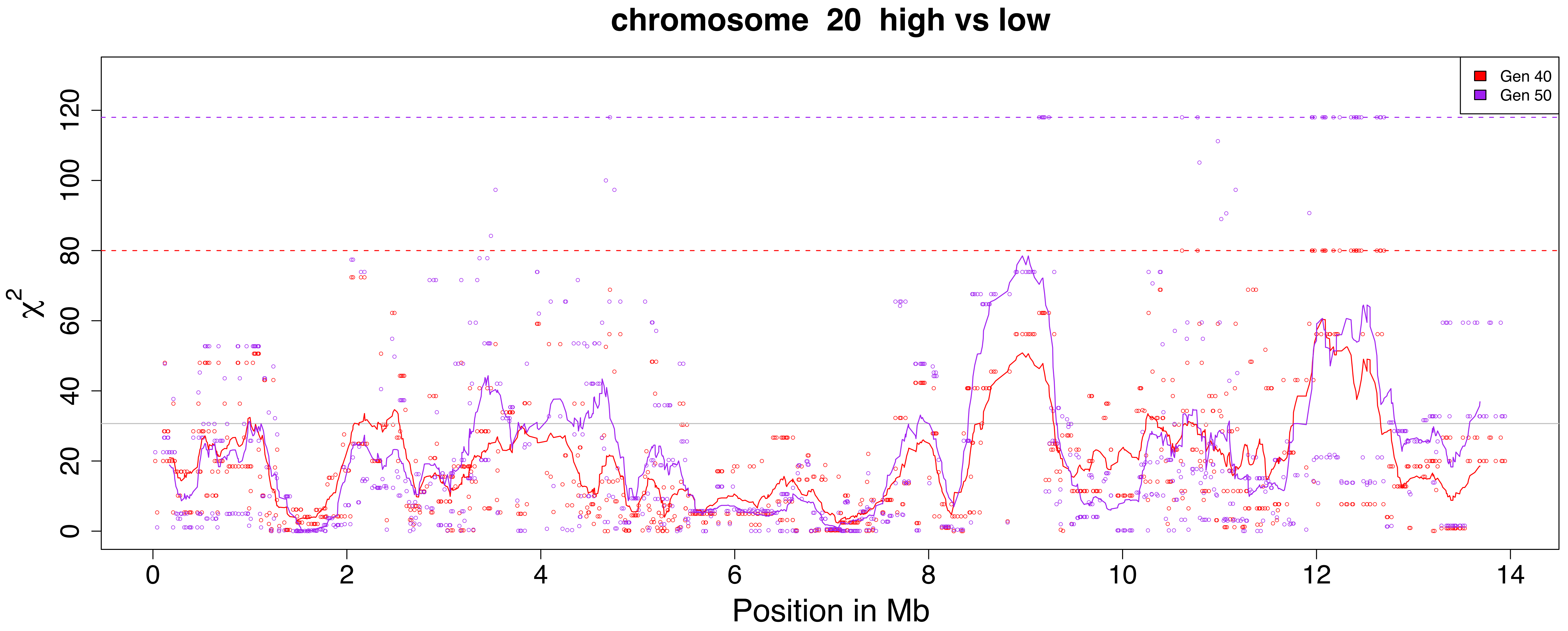

chromosome 20 high vs low
Gen 40
Gen 50
χ2
0
20
40
60
80
100
120
0
2
4
6
8
10
12
14
Position in Mb

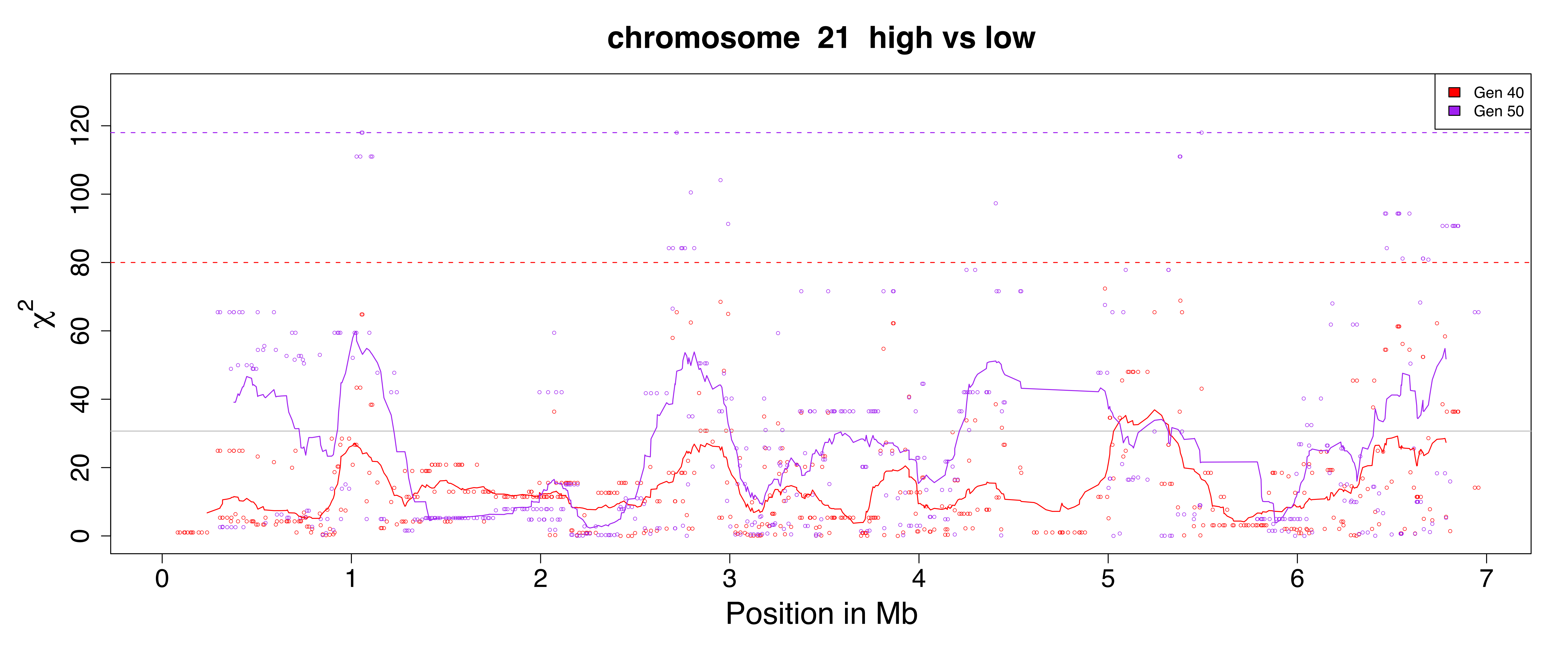

chromosome 21 high vs low
Gen 40
Gen 50
χ^2
0
20
40
60
80
100
120
0
1
2
3
4
5
6
7
Position in Mb

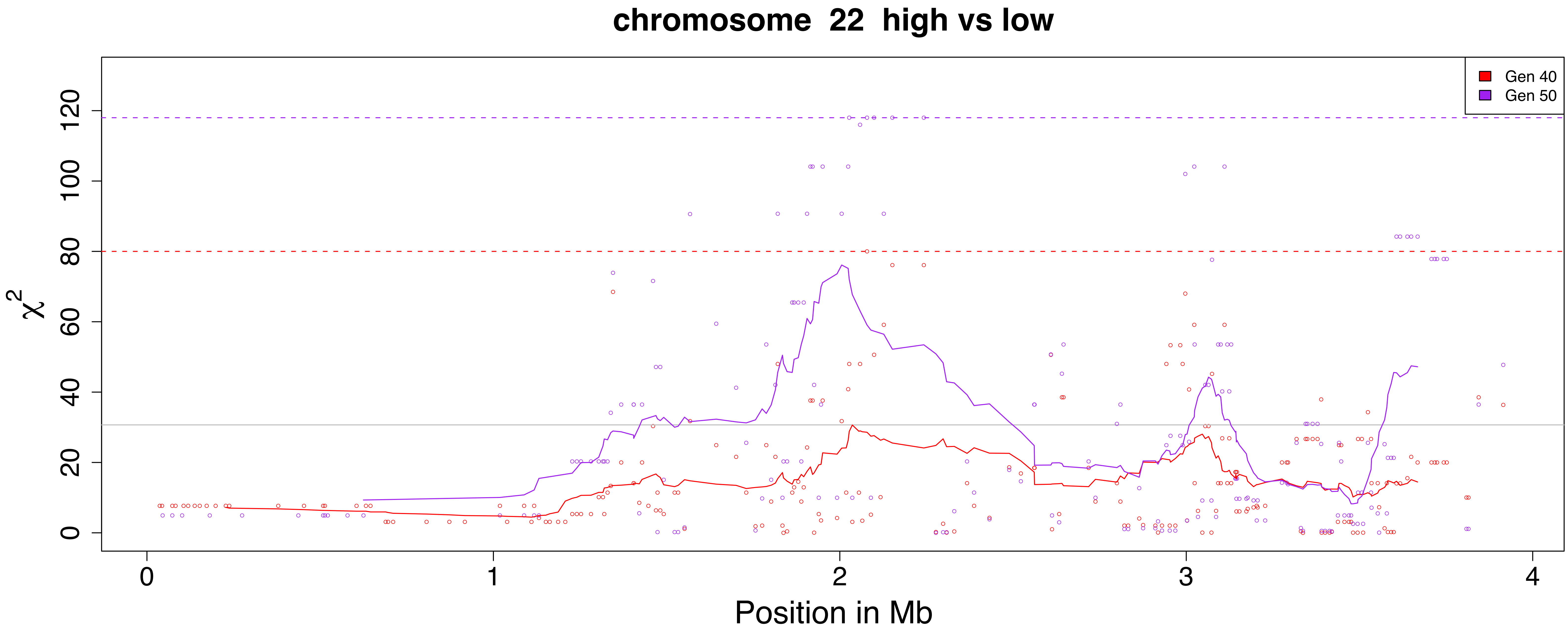
chromosome 22 high vs low
Gen 40
Gen 50
χ^2
0
20
40
60
80
100
120
0
1
2
3
4
Position in Mb

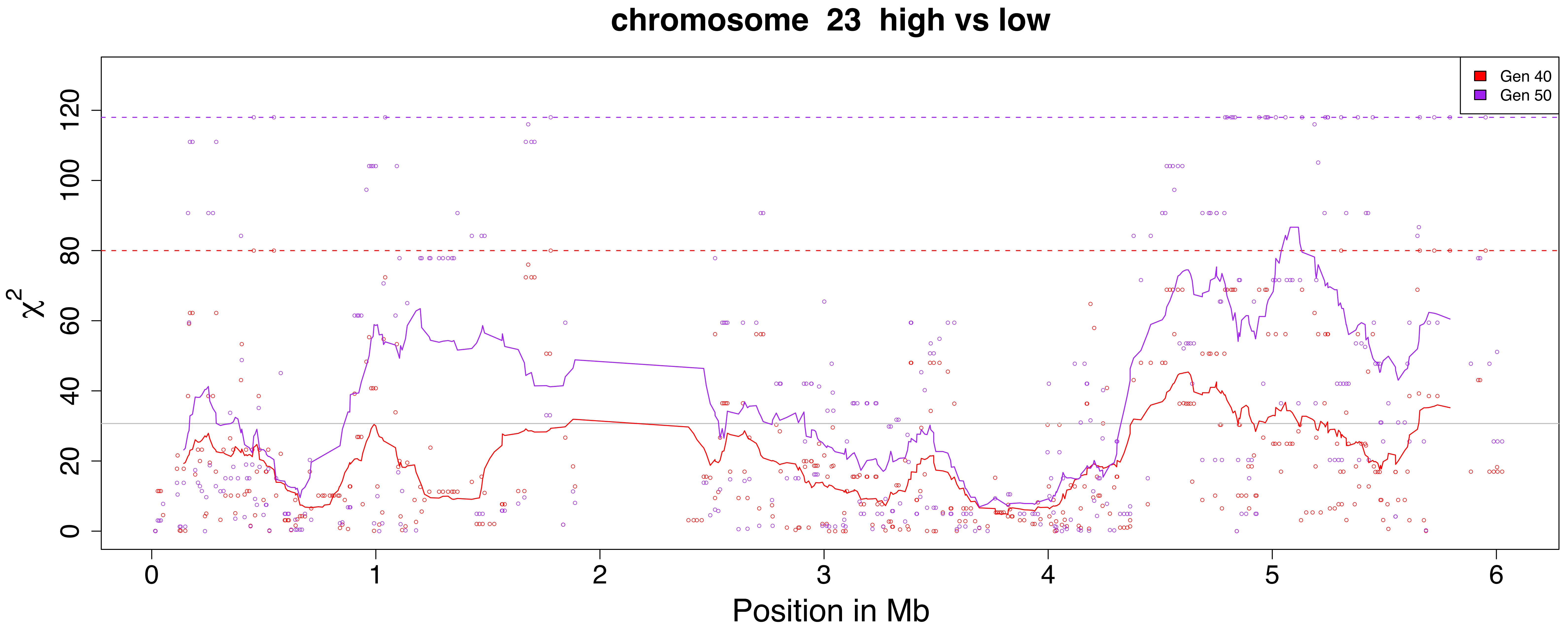

chromosome 23 high vs low
Gen 40
Gen 50
χ^2
0
20
40
60
80
100
120
0
1
2
3
4
5
6
Position in Mb

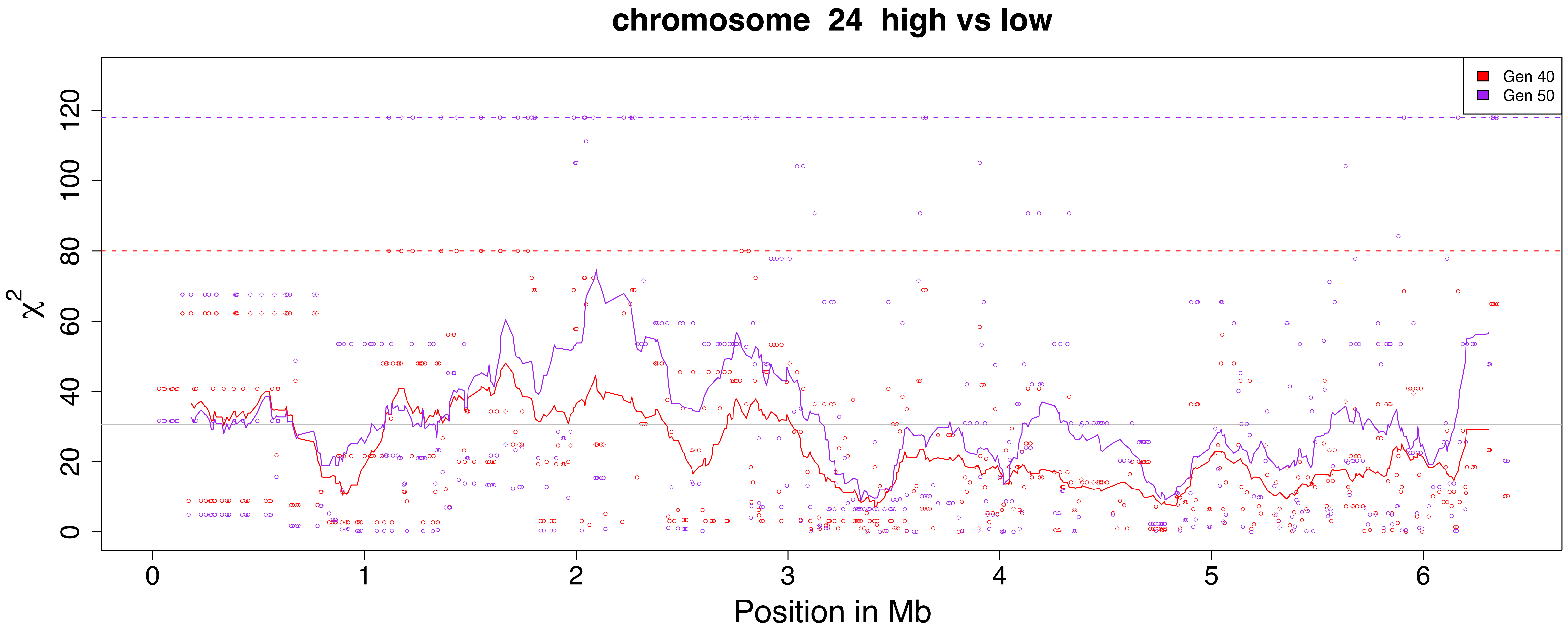
chromosome 24 high vs low
Gen 40
Gen 50
χ^2
0
20
40
60
80
100
120
0
1
2
3
4
5
6
Position in Mb

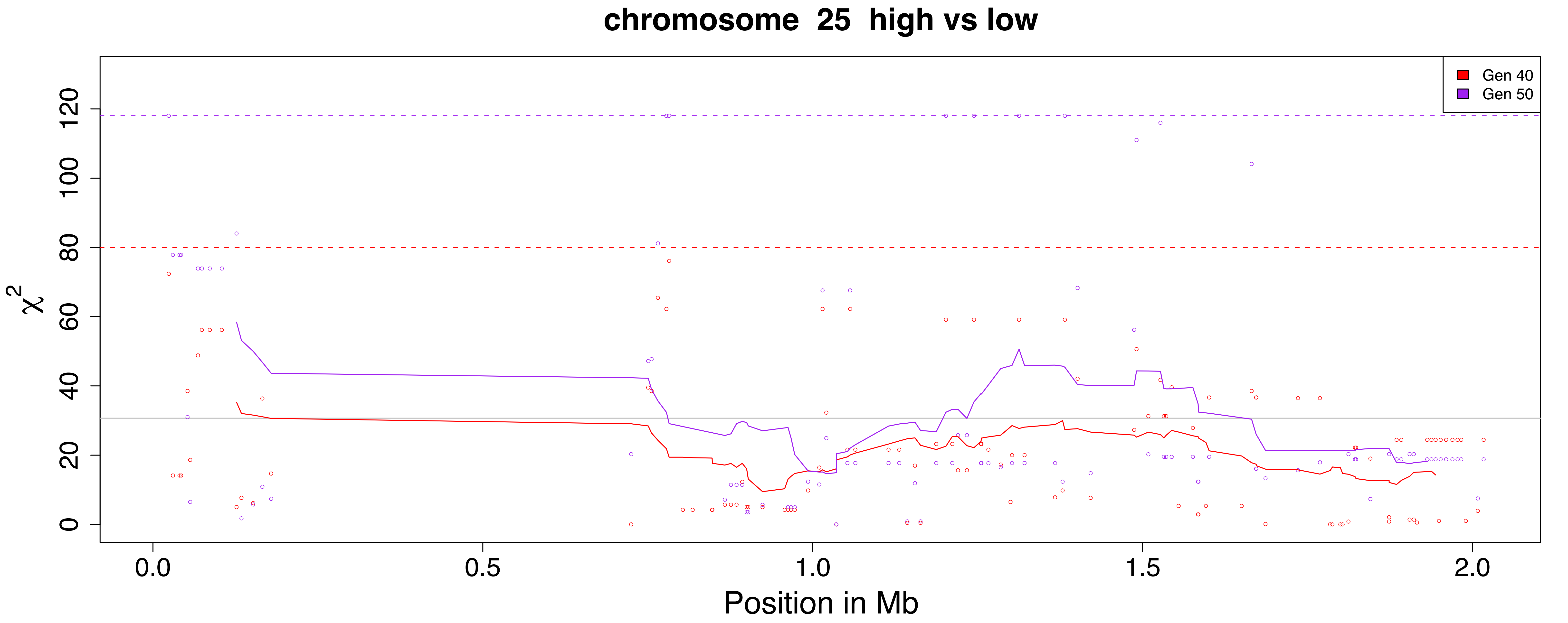
chromosome 25 high vs low
Gen 40
Gen 50
χ^2
0
20
40
60
80
100
120
0.0
0.5
1.0
1.5
2.0
Position in Mb

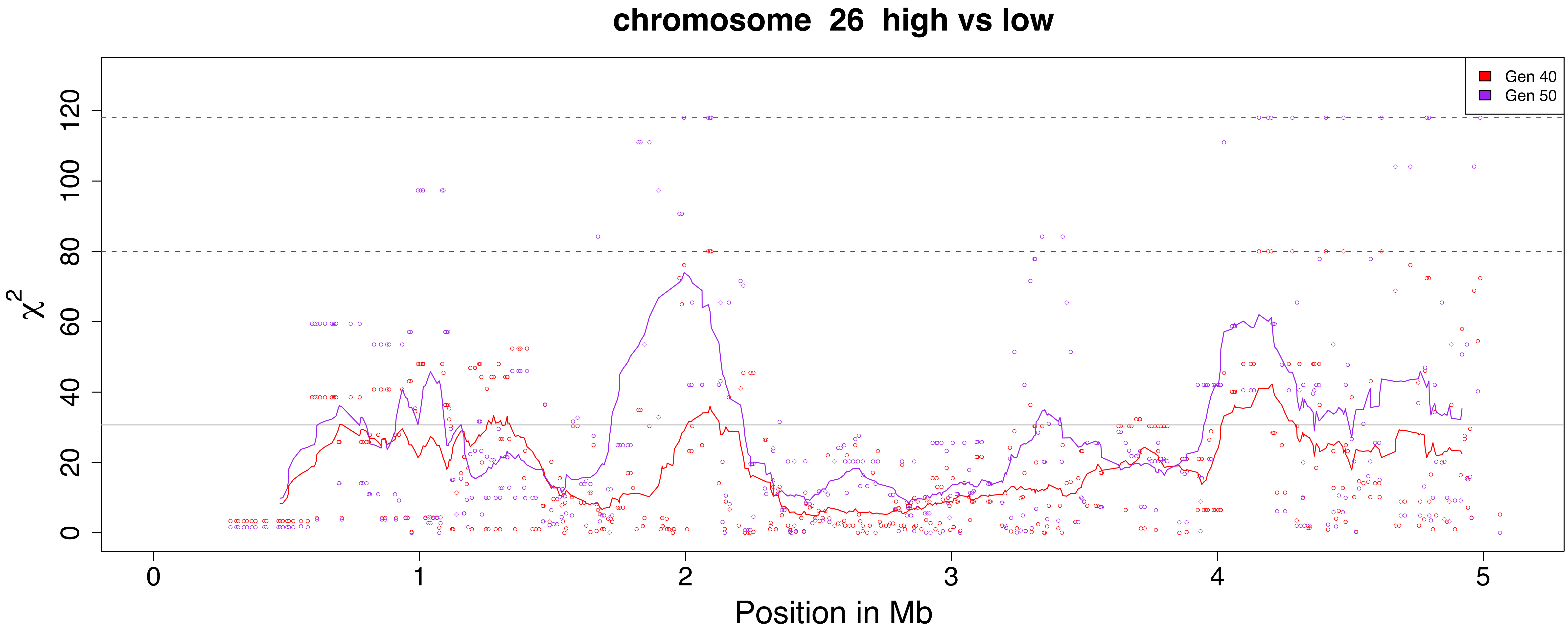

chromosome 26 high vs low
Gen 40
Gen 50
χ^2
0
20
40
60
80
100
120
0
1
2
3
4
5
Position in Mb

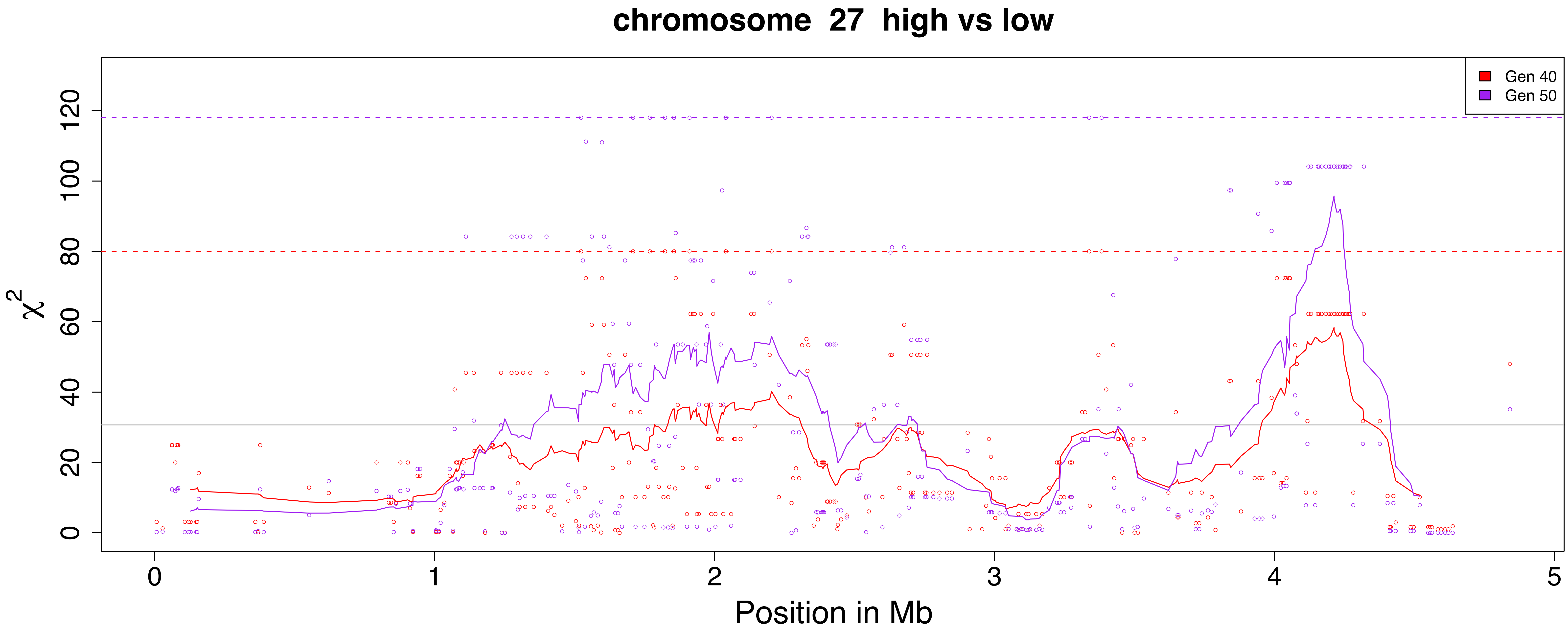

chromosome 27 high vs low
Gen 40
Gen 50
χ^2
0
20
40
60
80
100
120
0
1
2
3
4
5
Position in Mb

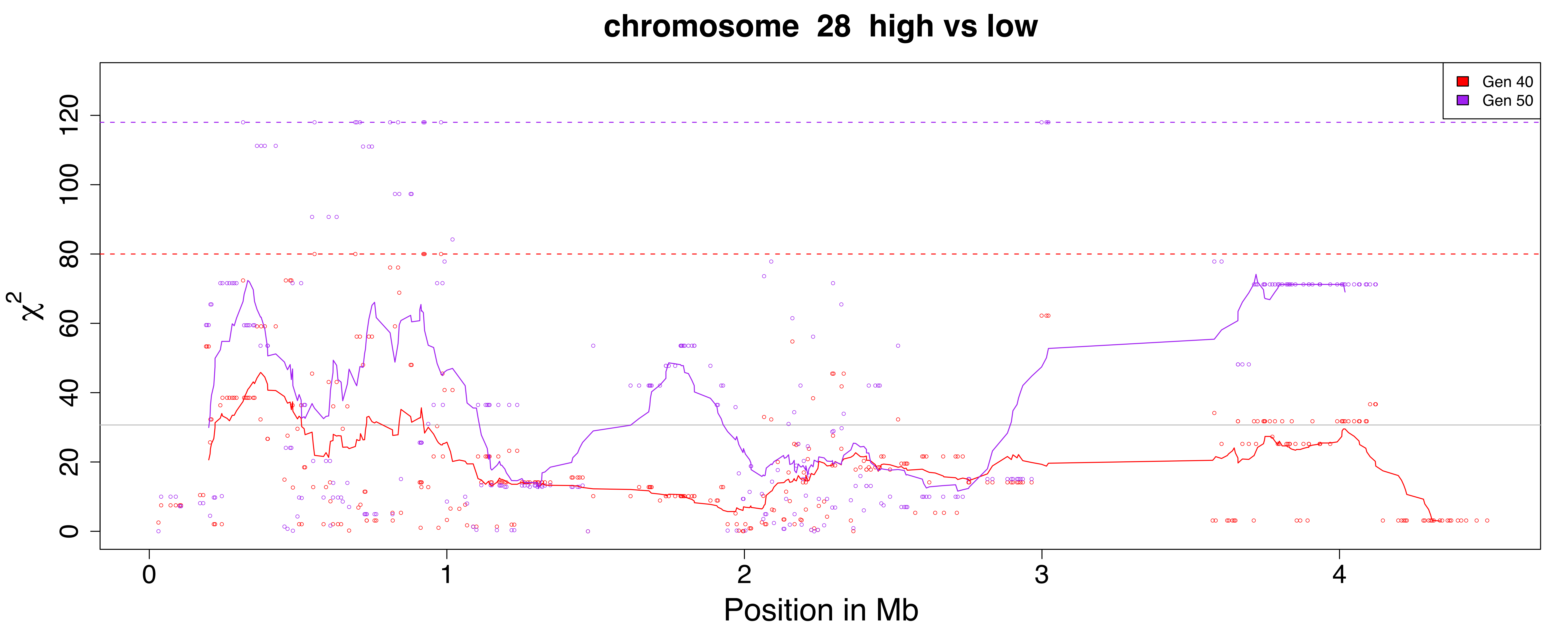

chromosome 28 high vs low
Gen 40
Gen 50
χ^2
0
20
40
60
80
100
120
0
1
2
3
4
Position in Mb

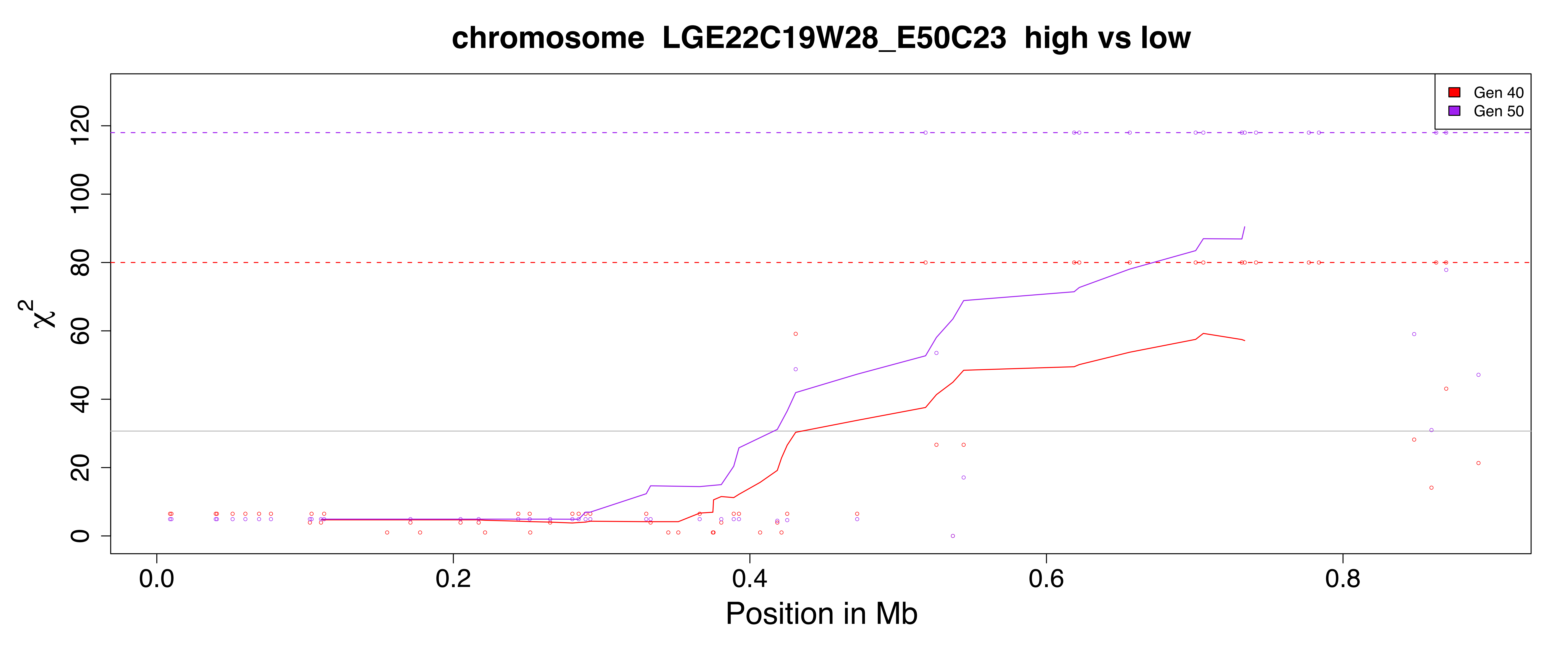

chromosome LGE22C19W28_E50C23 high vs low
Gen 40
Gen 50
χ2
0
20
40
60
80
100
120
0.0
0.2
0.4
0.6
0.8
Position in Mb

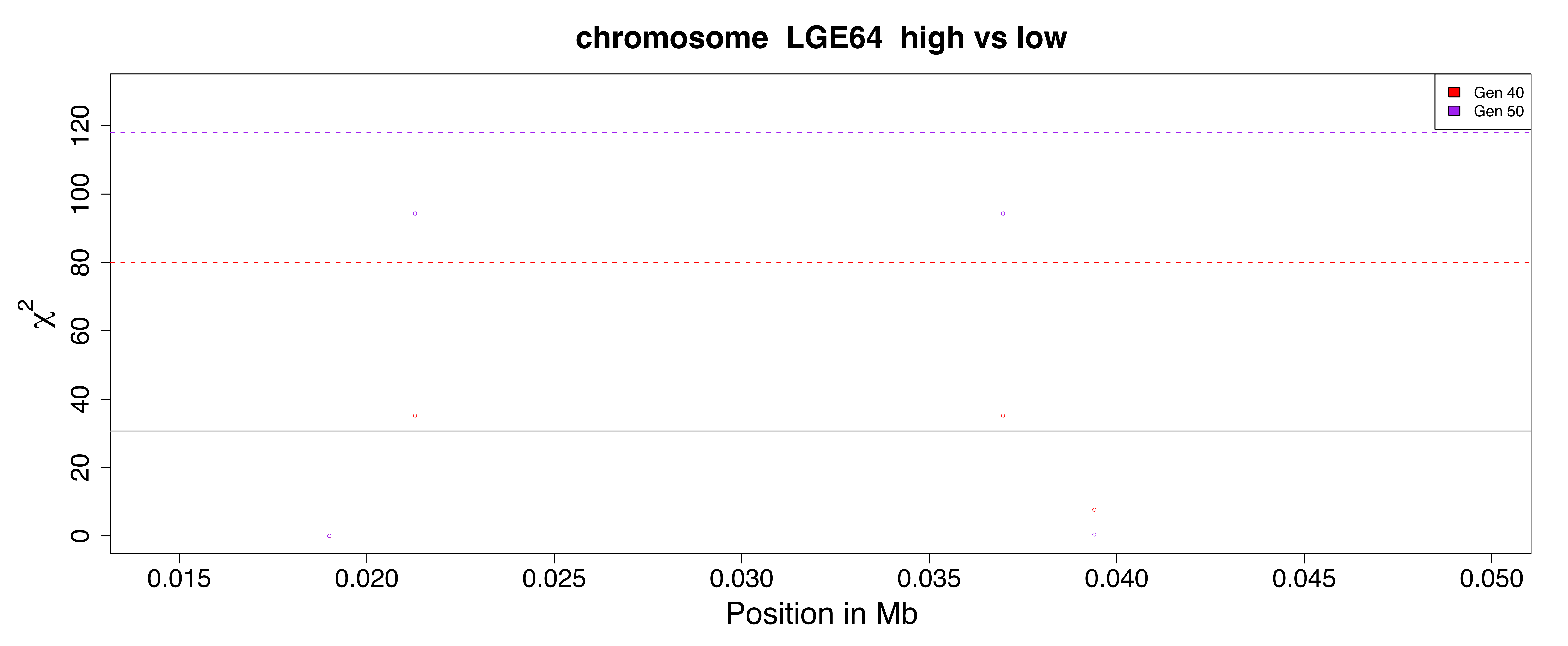
chromosome LGE64 high vs low
Gen 40
Gen 50
120
100
80
60
40
20
0
χ^2
0.015
0.020
0.025
0.030
0.035
0.040
0.045
0.050
Position in Mb

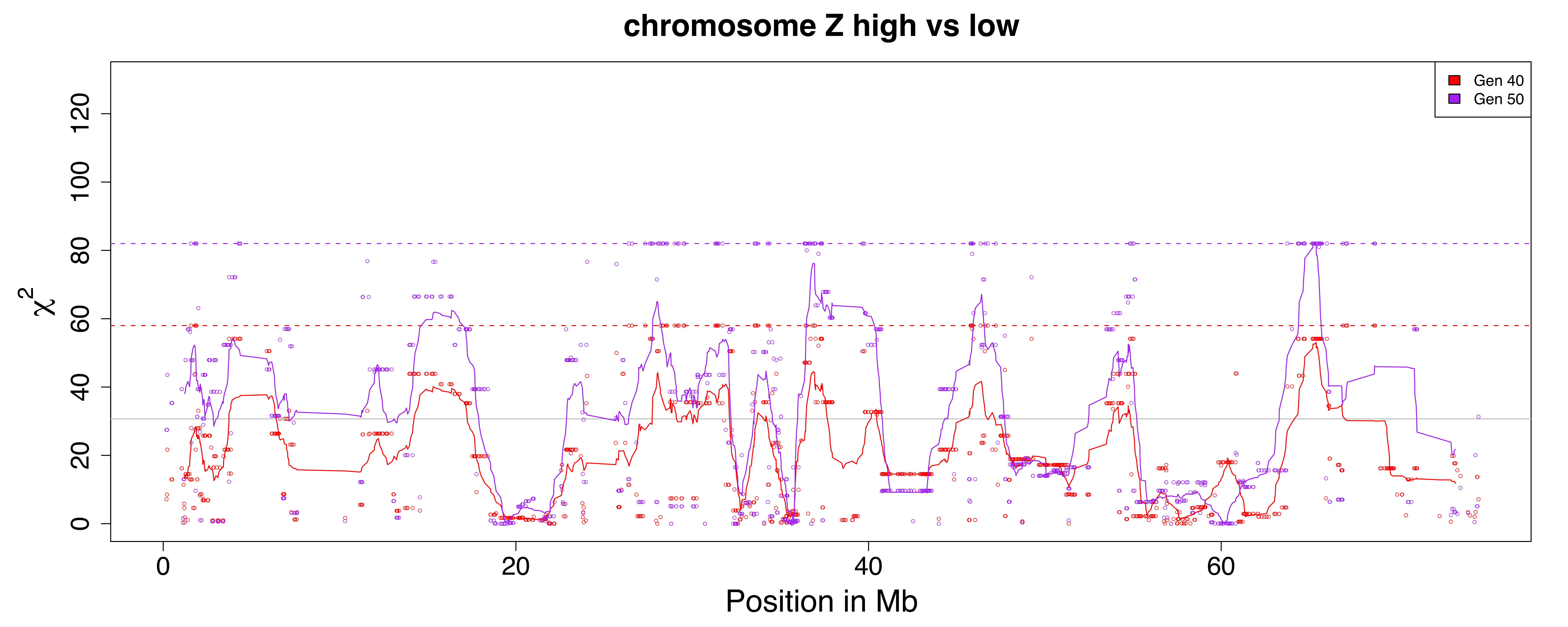
chromosome Z high vs low
Gen 40
Gen 50
χ^2
0
20
40
60
80
100
120
0
20
40
60
Position in Mb
