# Supplementary material for: Genome-Wide Effects of Long-Term Divergent Selection
Source: PLoS Genet. 2010 Nov 4;6(11):e1001188. doi: 10.1371/journal.pgen.1001188 (PMC2973821; doi:10.1371/journal.pgen.1001188)
Supplement: Figure S3 — Allele frequency changes between generation 40 and 50 across all chromosomes. Each symbol corresponds to the average frequency change over a block of 5 markers. Colored symbols indicate blocks that belong to the 95:th percentile, compared to all blocks in the entire genome. The blue and red lines indicate the number of outliers present in a window of 20 blocks. The largest changes are in several regions on chromosomes 1, 2 and 3 as well as in regions on chromosomes 7, 9, 11, 12, 18, 20 and 22. Changes on chromosomes 9 and 12 are most prominent in the high line, whereas changes on chromosome 2, 3 and 11 are mostly in the low line. Chromosome 20 is affected in both lines, but in the high line the changes are located towards the end of the chromosome and in the low line the first third is changing rapidly. Similarly, chromosome 22 has distinct regions affected in the different lines. On chromosome 18 a region changes rapidly in both lines, although the favoured allele differs for many, but not all, affected markers. (0.37 MB PDF) [file pgen.1001188.s003.pdf]

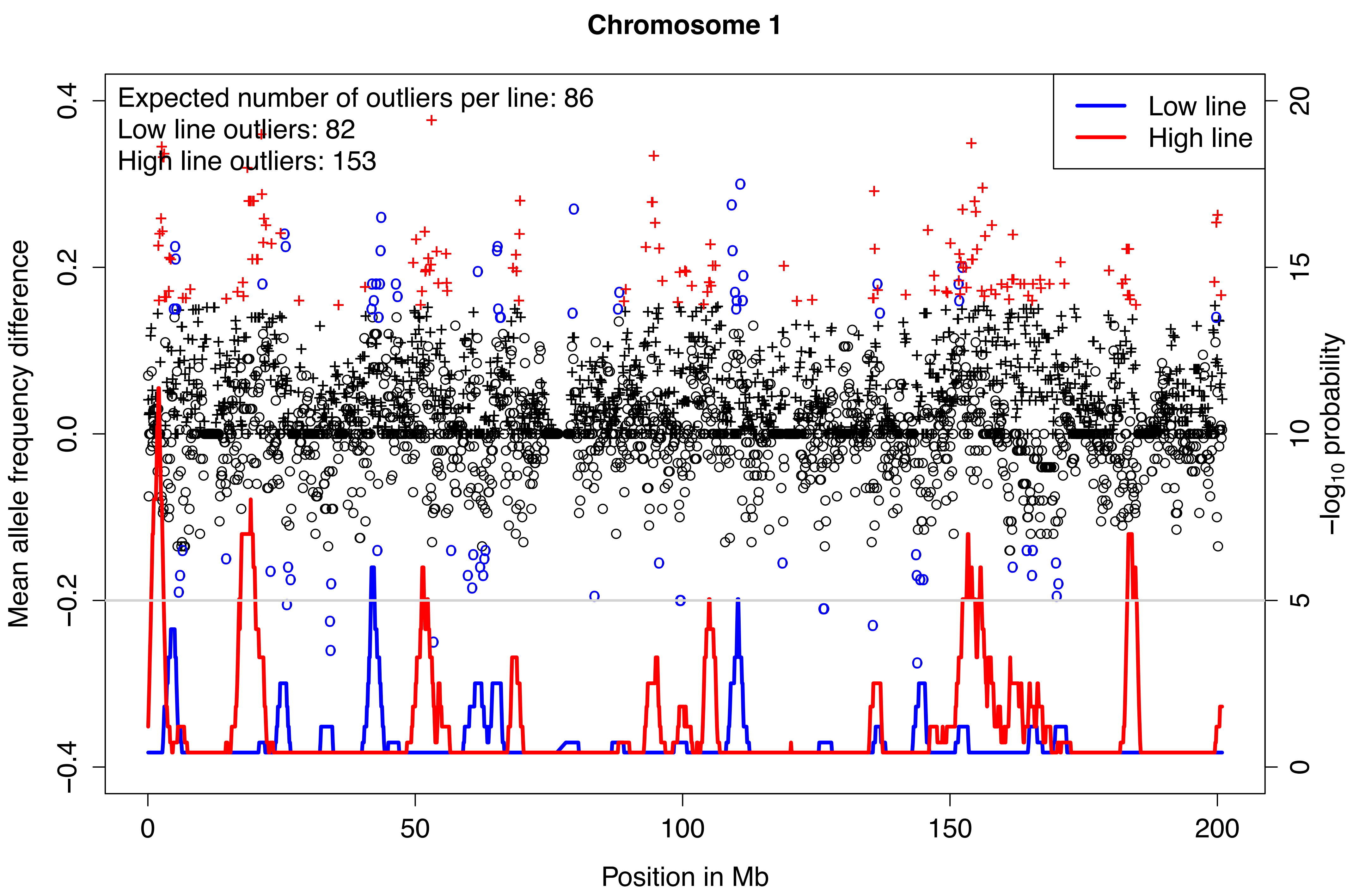

Chromosome 1
Expected number of outliers per line: 86
Low line outliers: 82
High line outliers: 153
Low line
High line
Mean allele frequency difference
−log10 probability
Position in Mb
0.4
0.2
0.0
−0.2
−0.4
20
15
10
5
0
50
100
150
200

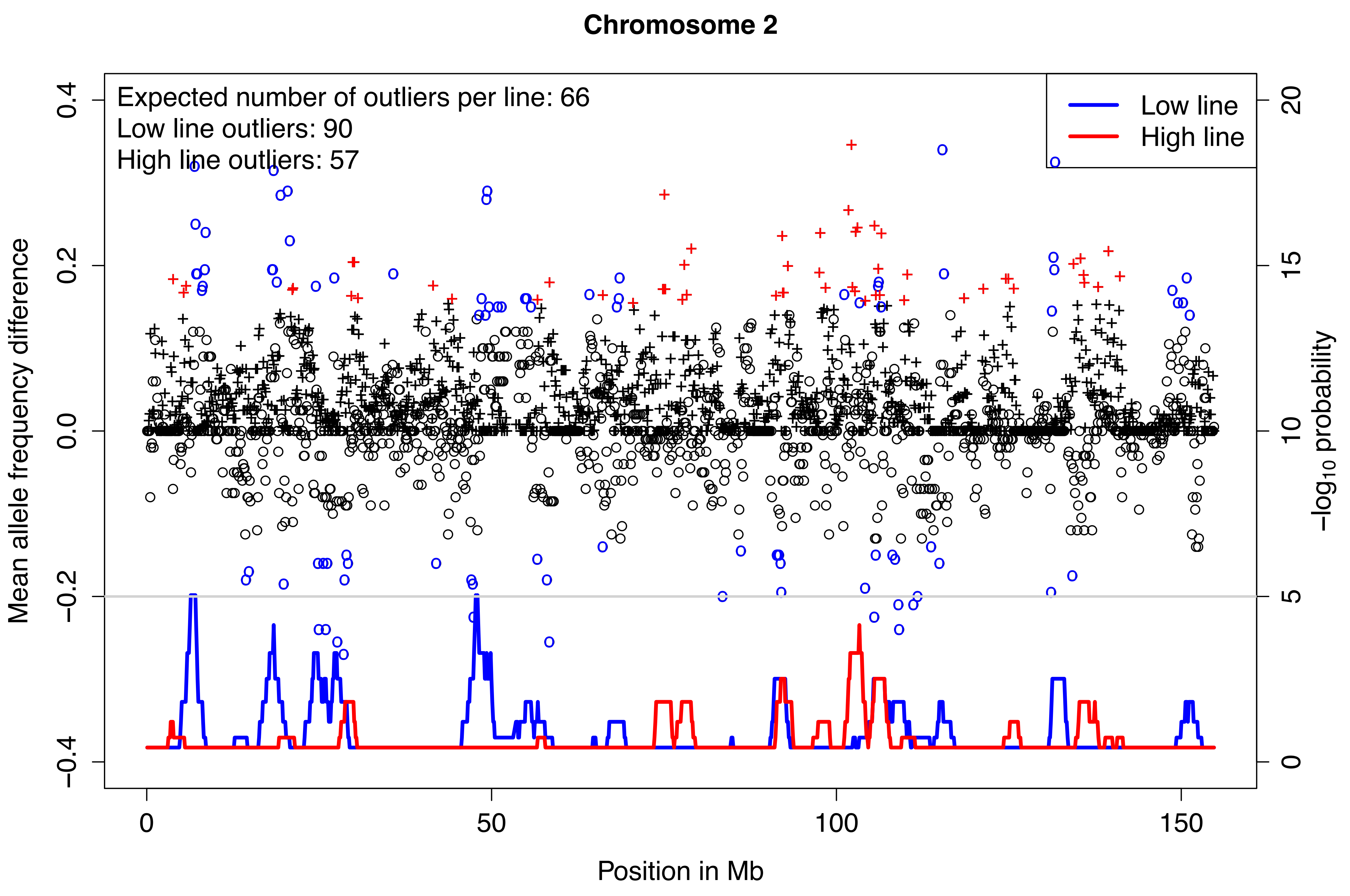

Chromosome 2
Expected number of outliers per line: 66
Low line outliers: 90
High line outliers: 57
Low line
High line
Mean allele frequency difference
−log10 probability
Position in Mb
0.4
0.2
0.0
−0.2
−0.4
20
15
10
5
0
0
50
100
150

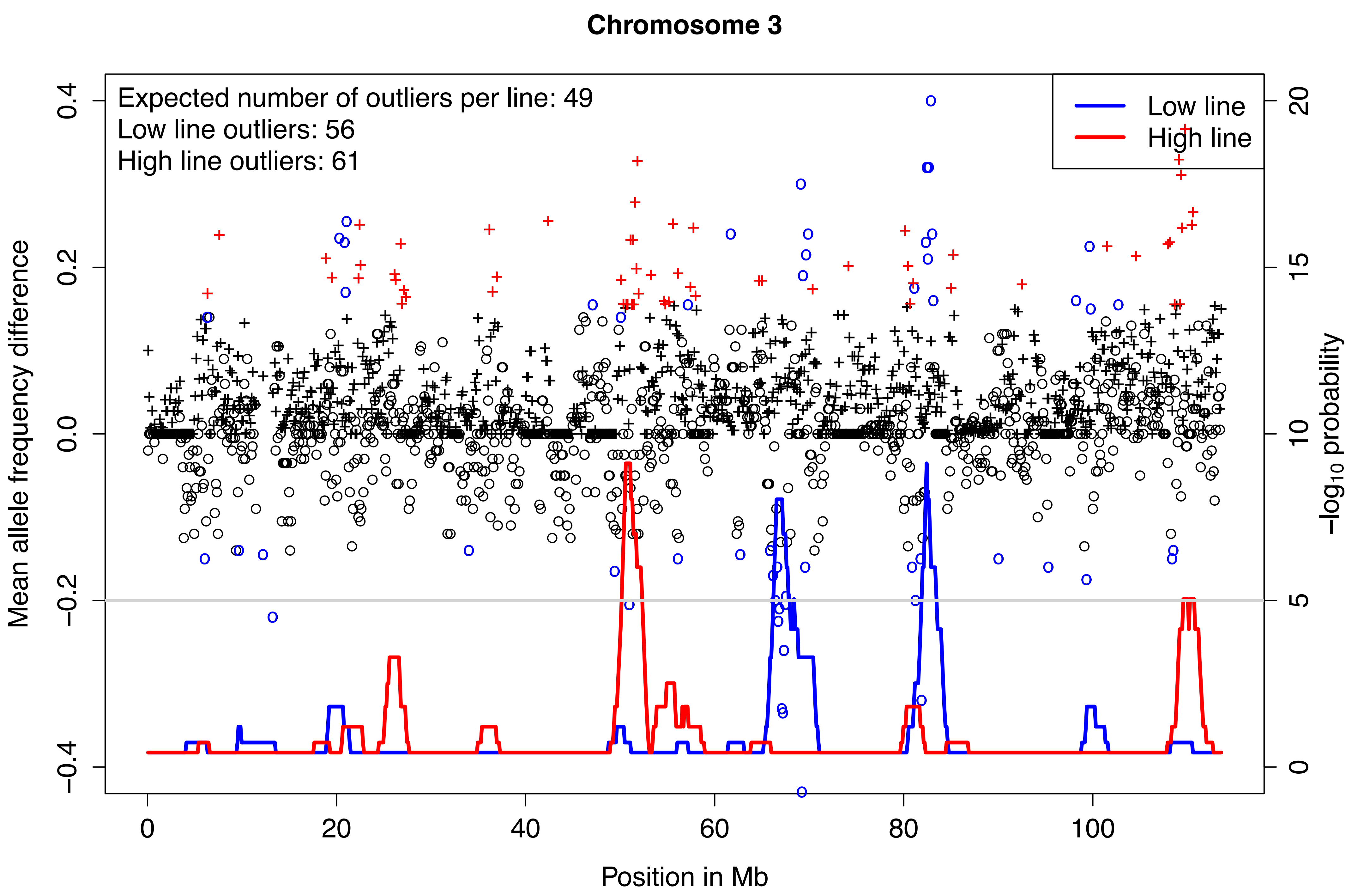
Chromosome 3
Expected number of outliers per line: 49
Low line outliers: 56
High line outliers: 61
Low line
High line
Mean allele frequency difference
−log10 probability
Position in Mb
0.4
0.2
0.0
−0.2
−0.4
20
15
10
5
0
0
20
40
60
80
100

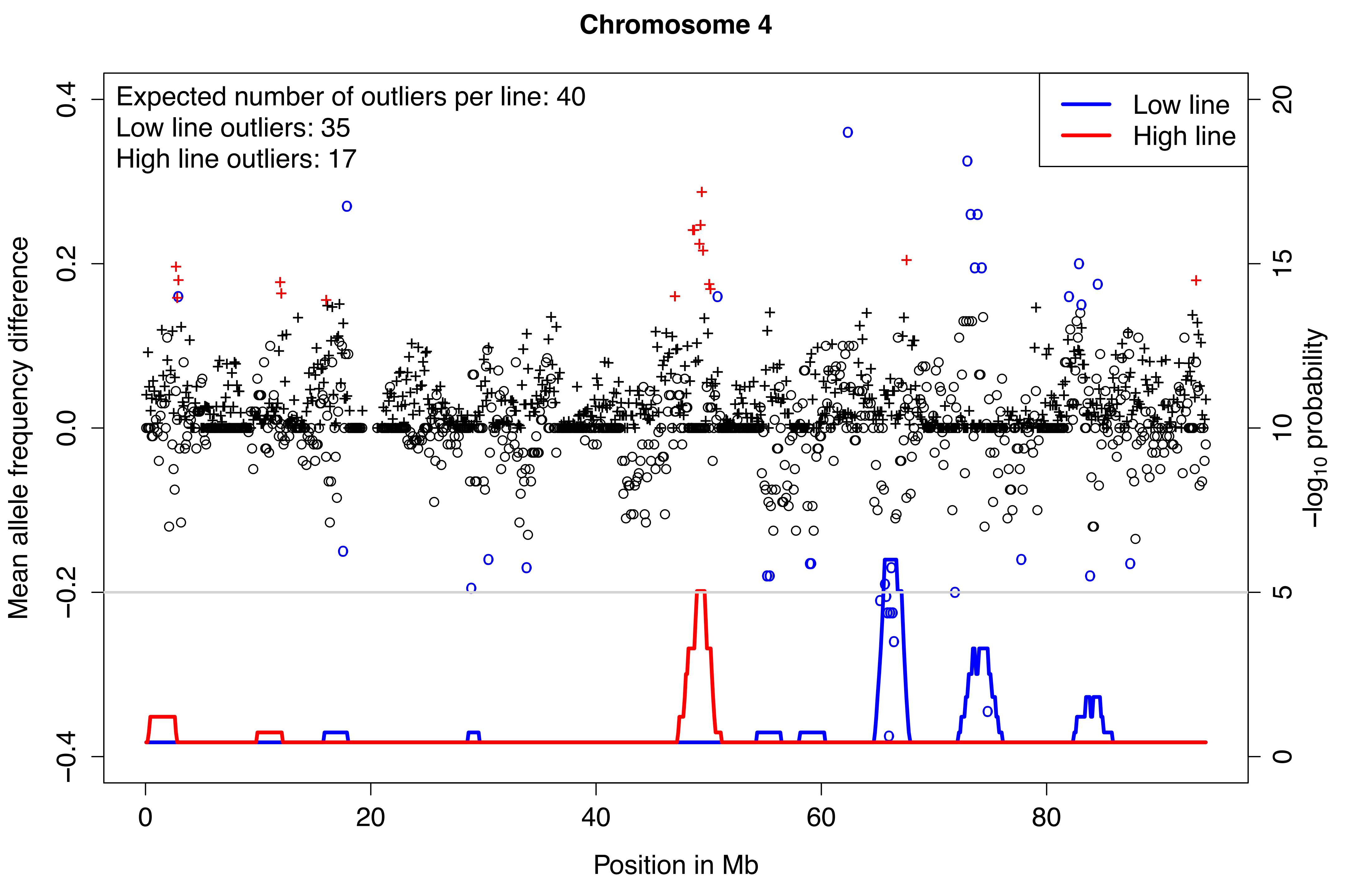
Chromosome 4
Expected number of outliers per line: 40
Low line outliers: 35
High line outliers: 17
Low line
High line
Mean allele frequency difference
−log10 probability
Position in Mb
0.4
0.2
0.0
−0.2
−0.4
20
15
10
5
0
0
20
40
60
80

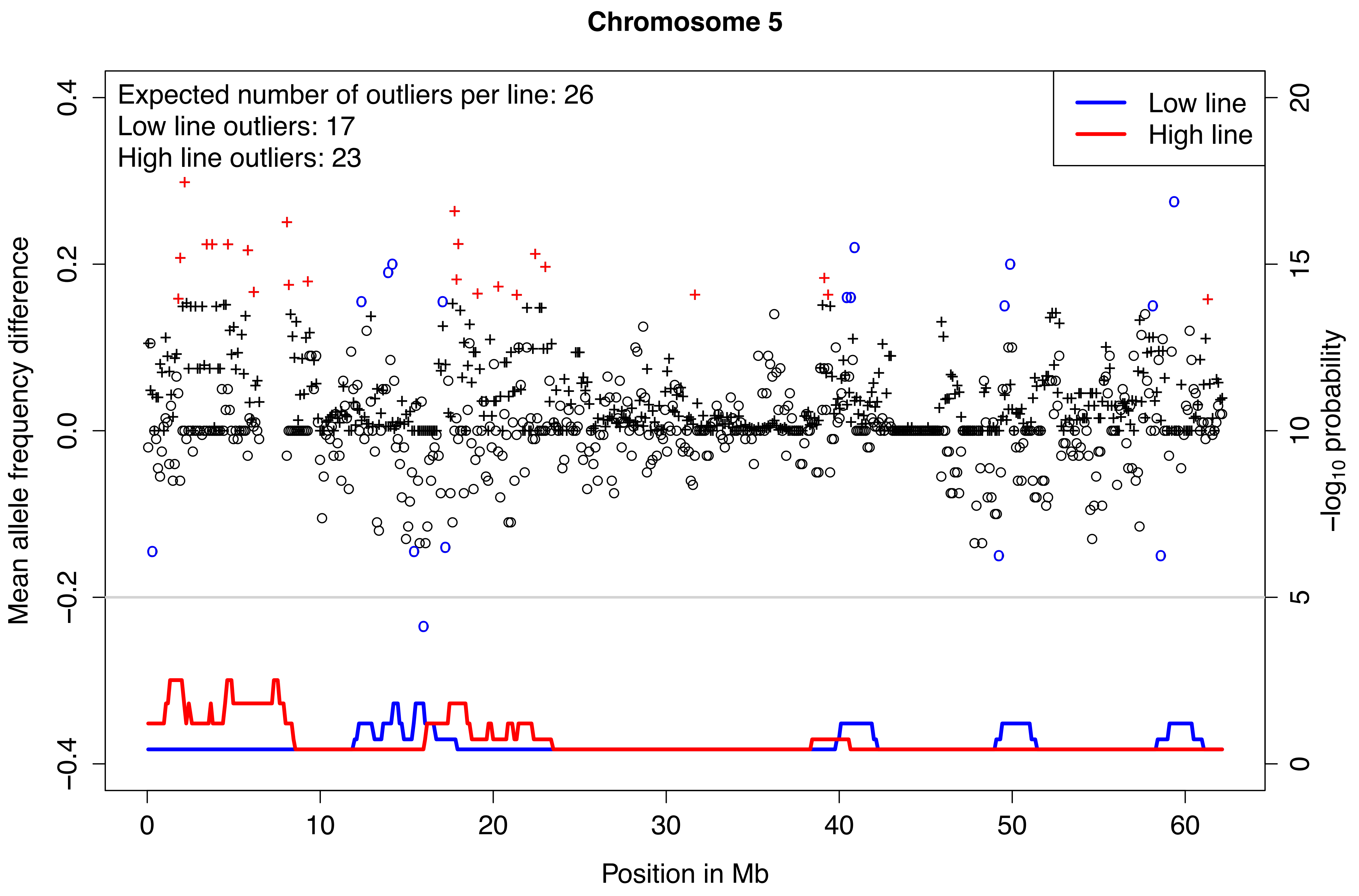

Chromosome 5
Expected number of outliers per line: 26
Low line outliers: 17
High line outliers: 23
Low line
High line
Mean allele frequency difference
−log10 probability
Position in Mb
0.4
0.2
0.0
−0.2
−0.4
20
15
10
5
0
0
10
20
30
40
50
60

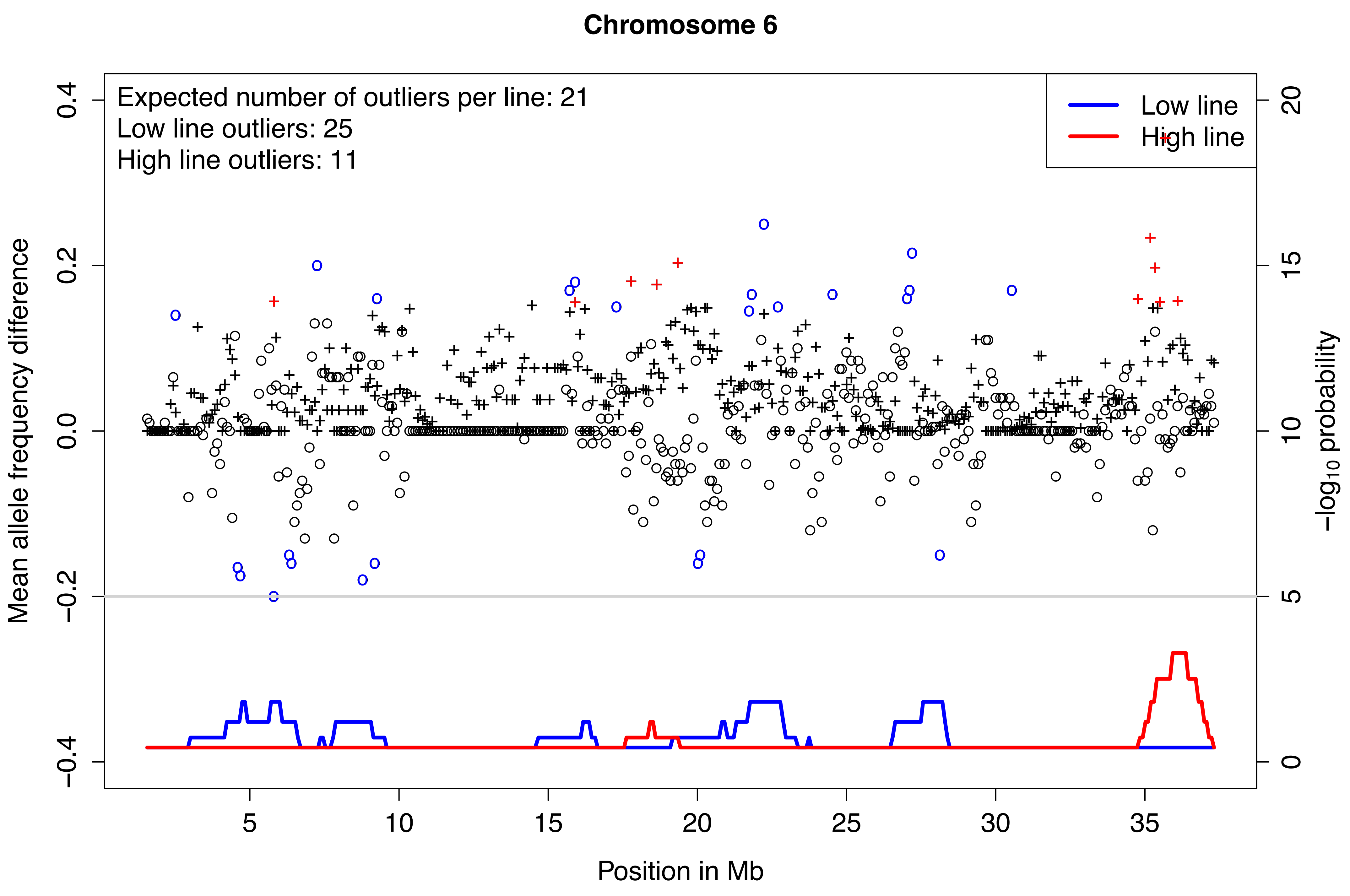

Chromosome 6
Expected number of outliers per line: 21
Low line outliers: 25
High line outliers: 11
Low line
High line
Mean allele frequency difference
0.4
0.2
0.0
−0.2
−0.4
−log10 probability
20
15
10
5
0
5
10
15
20
25
30
35
Position in Mb

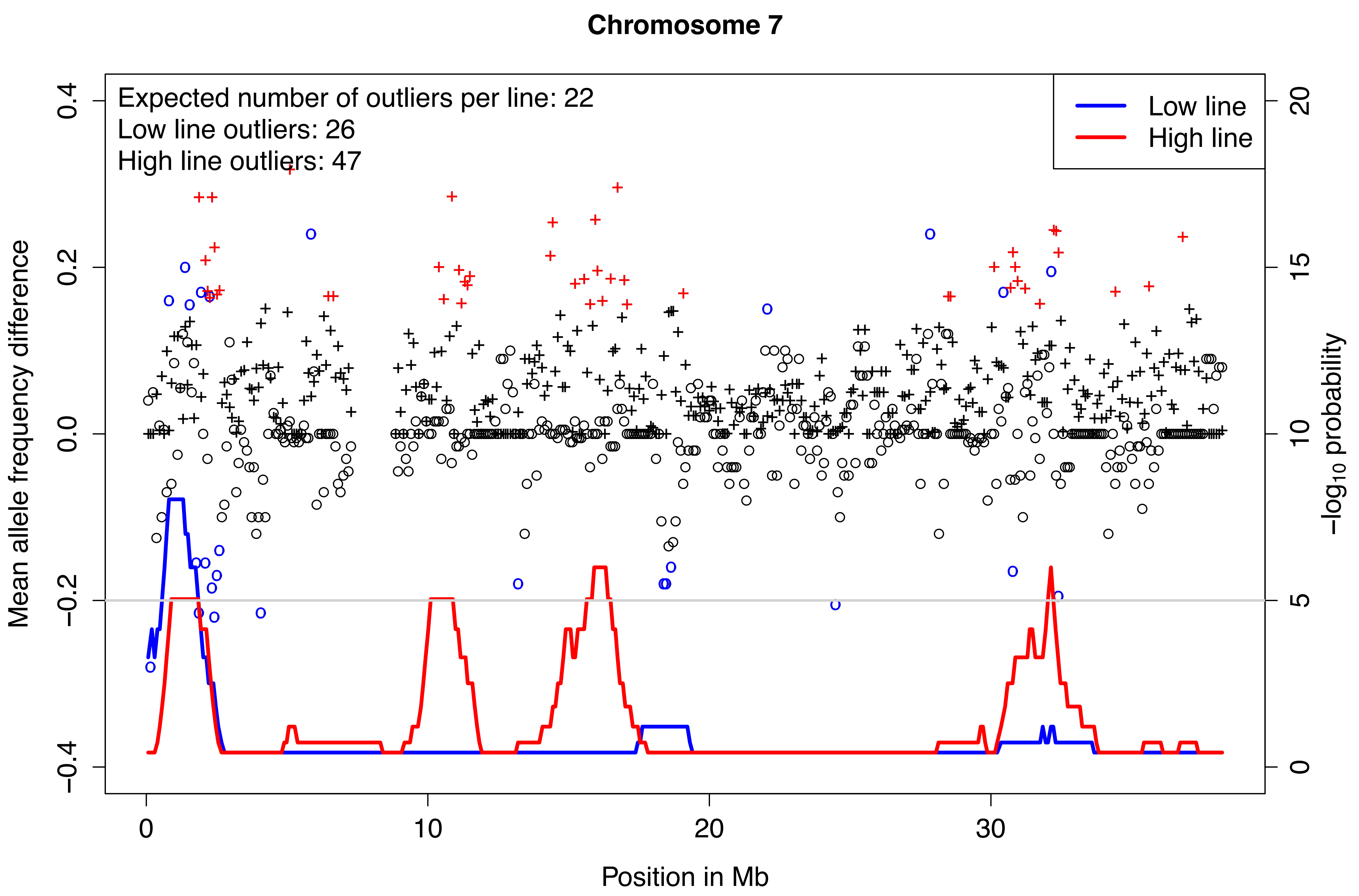

Chromosome 7
Expected number of outliers per line: 22
Low line outliers: 26
High line outliers: 47
Low line
High line
Mean allele frequency difference
−log10 probability
Position in Mb
0.4
0.2
0.0
−0.2
−0.4
20
15
10
5
0
0
10
20
30

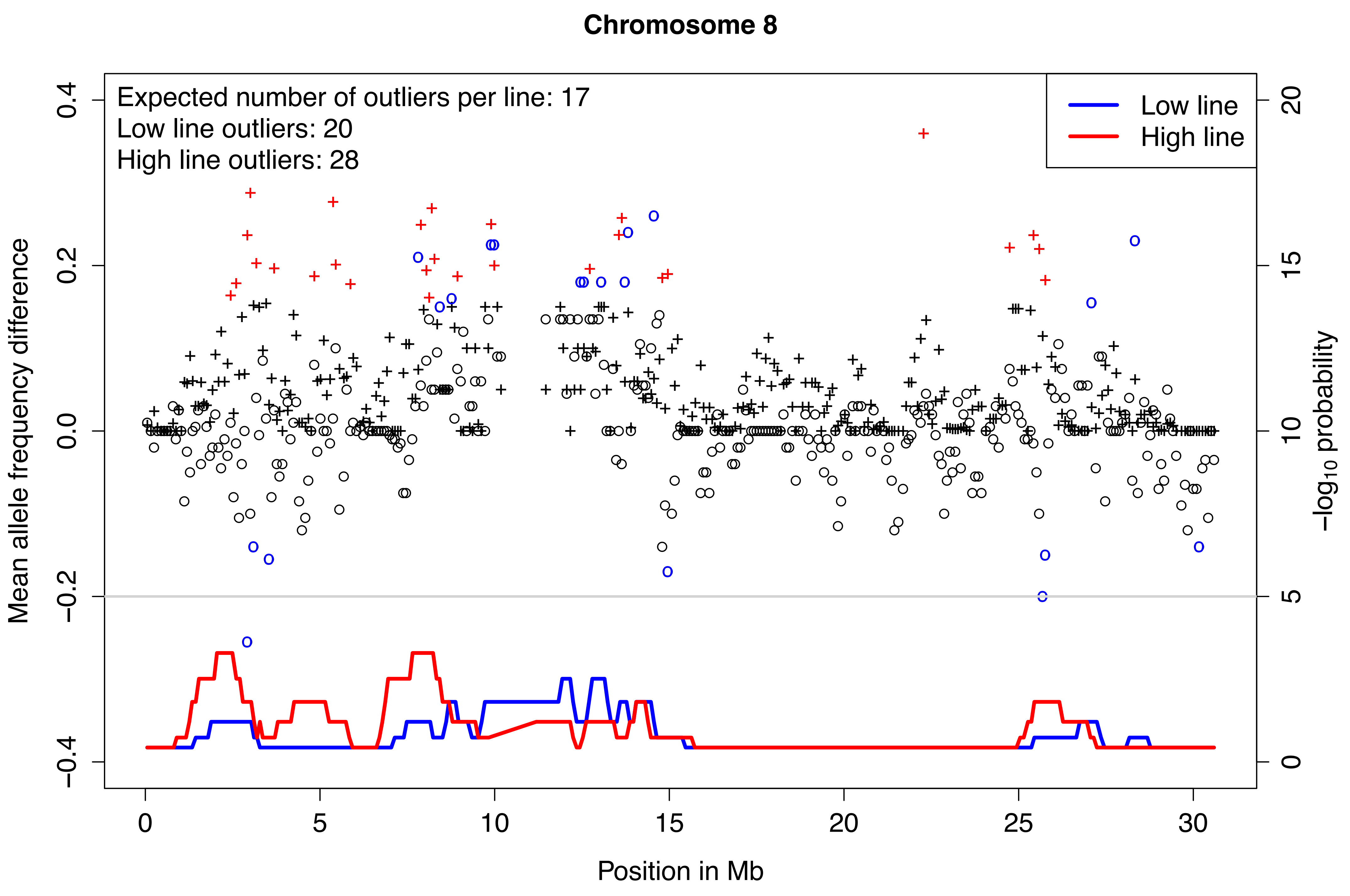

Chromosome 8
Expected number of outliers per line: 17
Low line outliers: 20
High line outliers: 28
Low line
High line
Mean allele frequency difference
−log10 probability
Position in Mb
0.4
0.2
0.0
−0.2
−0.4
20
15
10
5
0
0
5
10
15
20
25
30

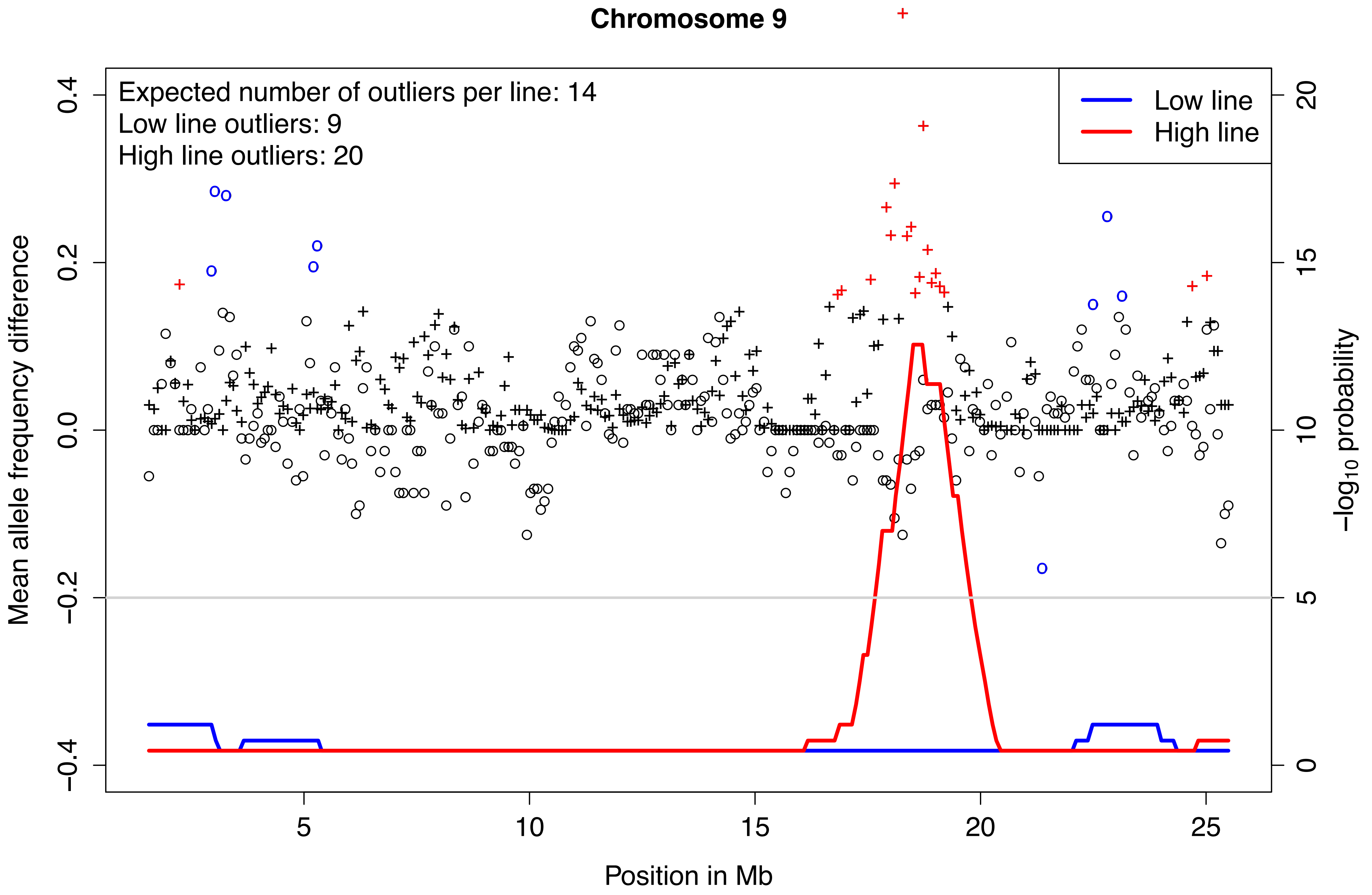

Chromosome 9
Expected number of outliers per line: 14
Low line outliers: 9
High line outliers: 20
Low line
High line
Mean allele frequency difference
−log10 probability
Position in Mb
0.4
0.2
0.0
−0.2
−0.4
20
15
10
5
0
5
10
15
20
25

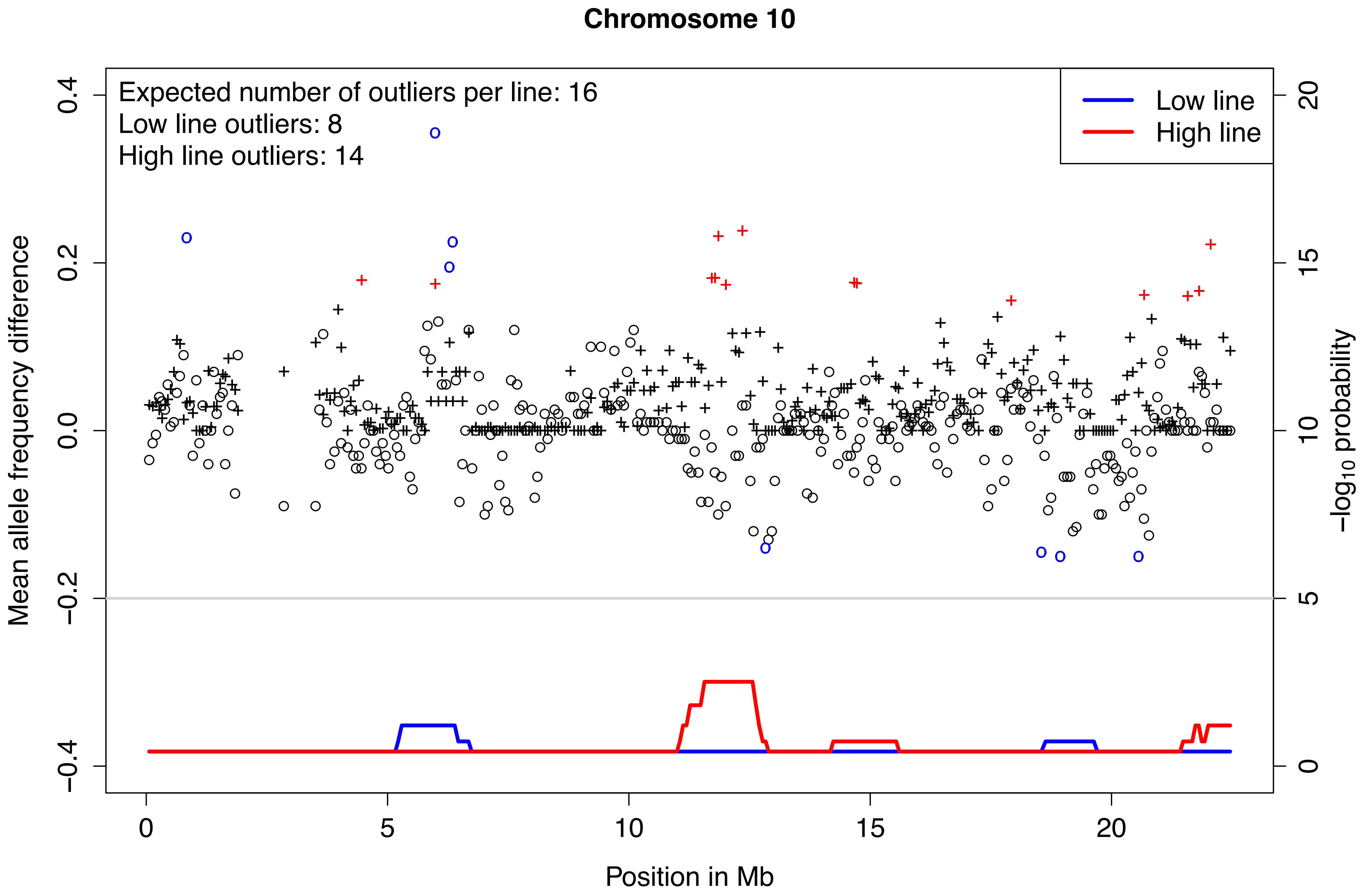

Chromosome 10
Expected number of outliers per line: 16
Low line outliers: 8
High line outliers: 14
Low line
High line
Mean allele frequency difference
−log10 probability
Position in Mb

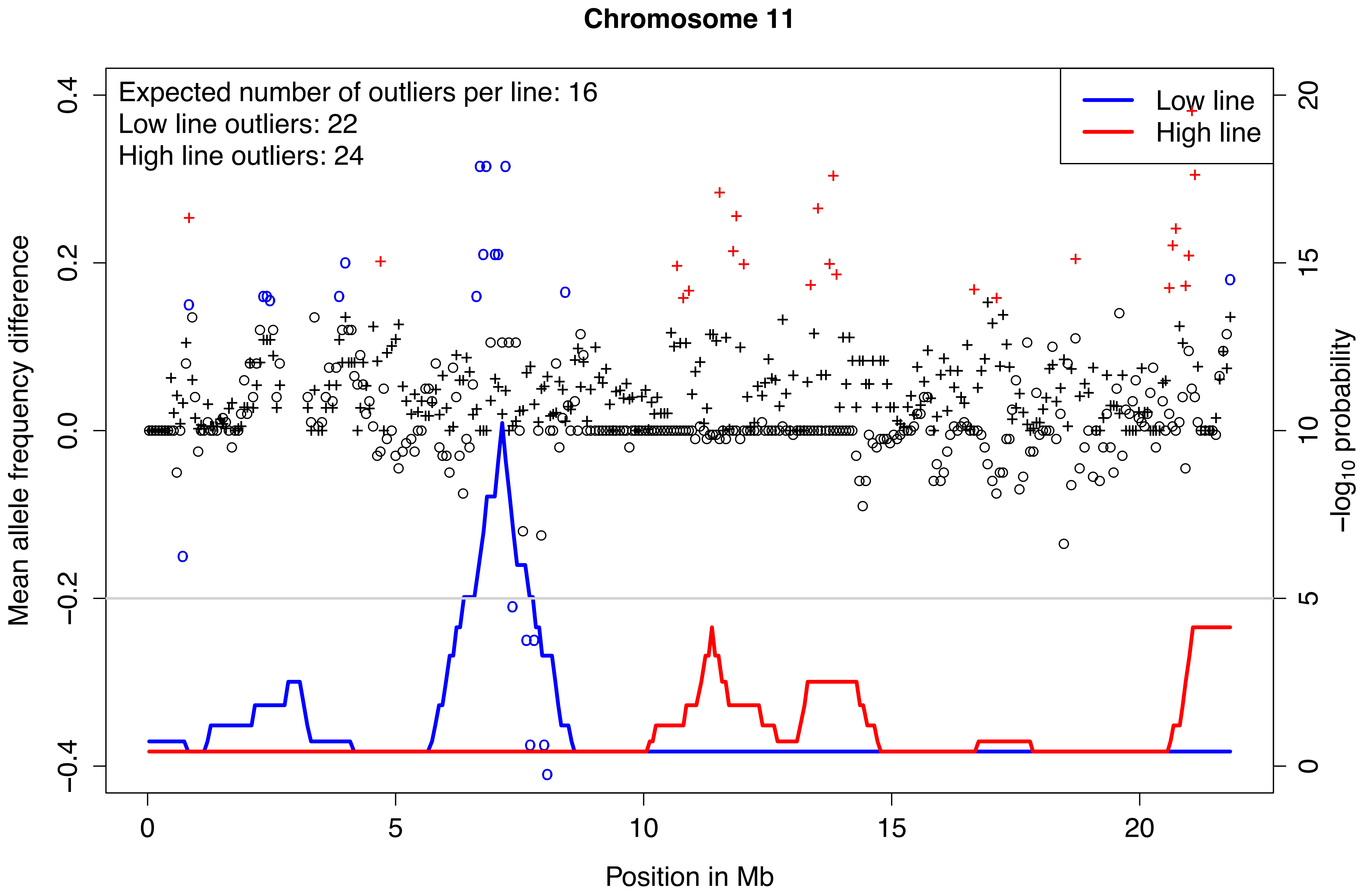

Chromosome 11
Expected number of outliers per line: 16
Low line outliers: 22
High line outliers: 24
Low line
High line
Mean allele frequency difference
−log10 probability
Position in Mb
0
5
10
15
20
0.4
0.2
0.0
−0.2
−0.4

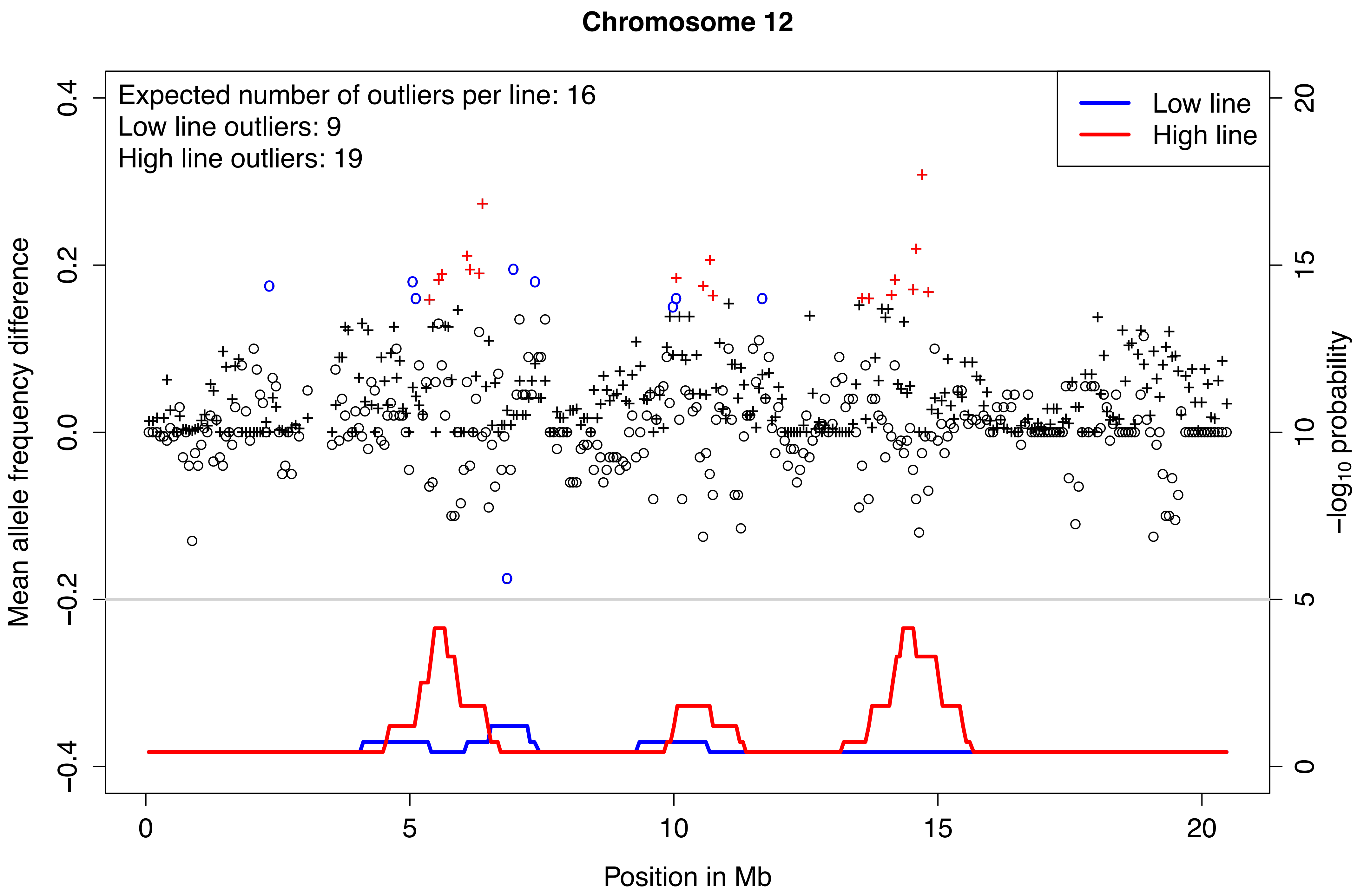

Chromosome 12
Expected number of outliers per line: 16
Low line outliers: 9
High line outliers: 19
Low line
High line
Mean allele frequency difference
−log10 probability
Position in Mb
0.4
0.2
0.0
−0.2
−0.4
20
15
10
5
0
0
5
10
15
20

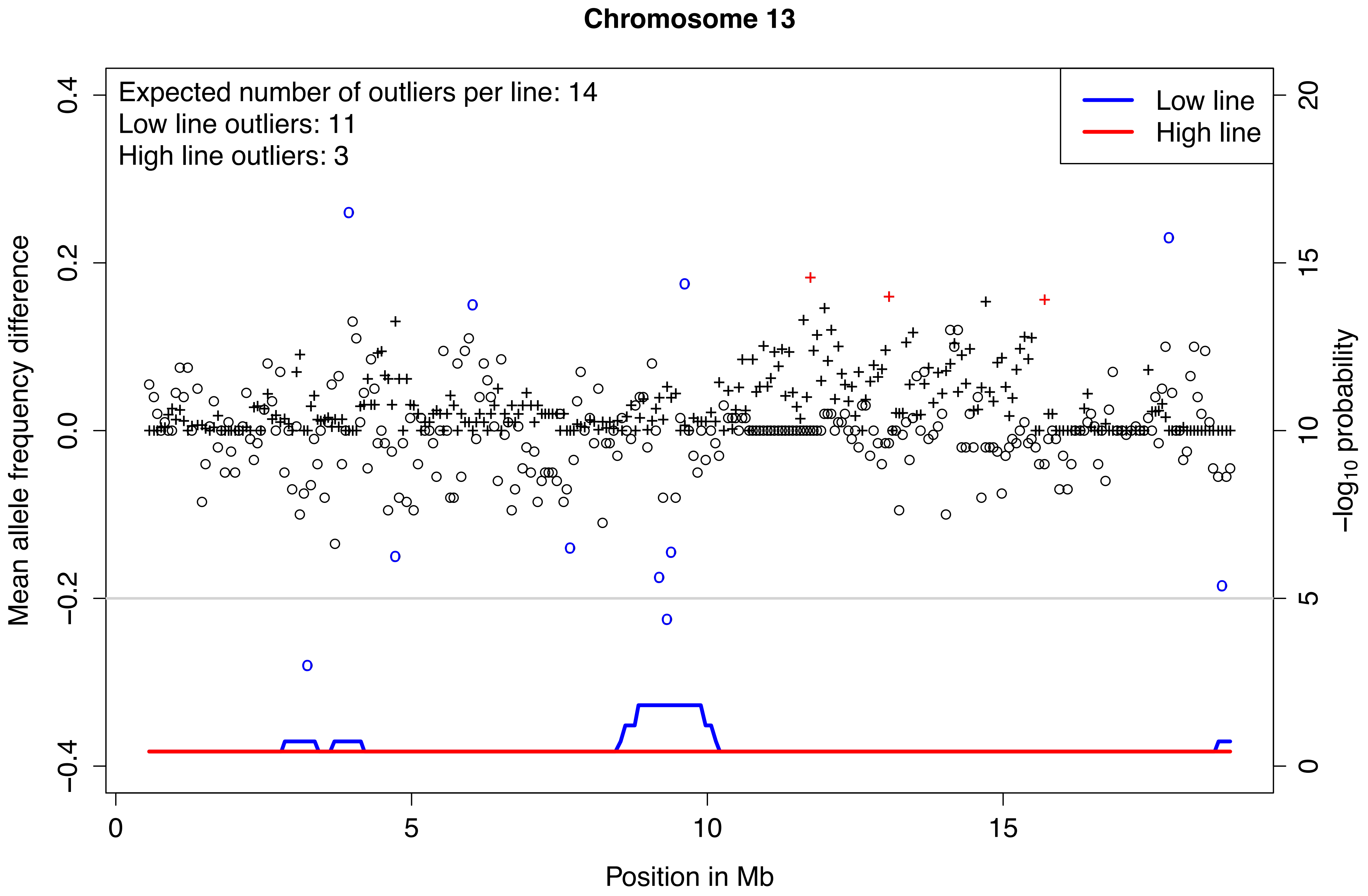

Chromosome 13
Expected number of outliers per line: 14
Low line outliers: 11
High line outliers: 3
Low line
High line
Mean allele frequency difference
−log10 probability
Position in Mb
0.4
0.2
0.0
−0.2
−0.4
20
15
10
5
0
0
5
10
15

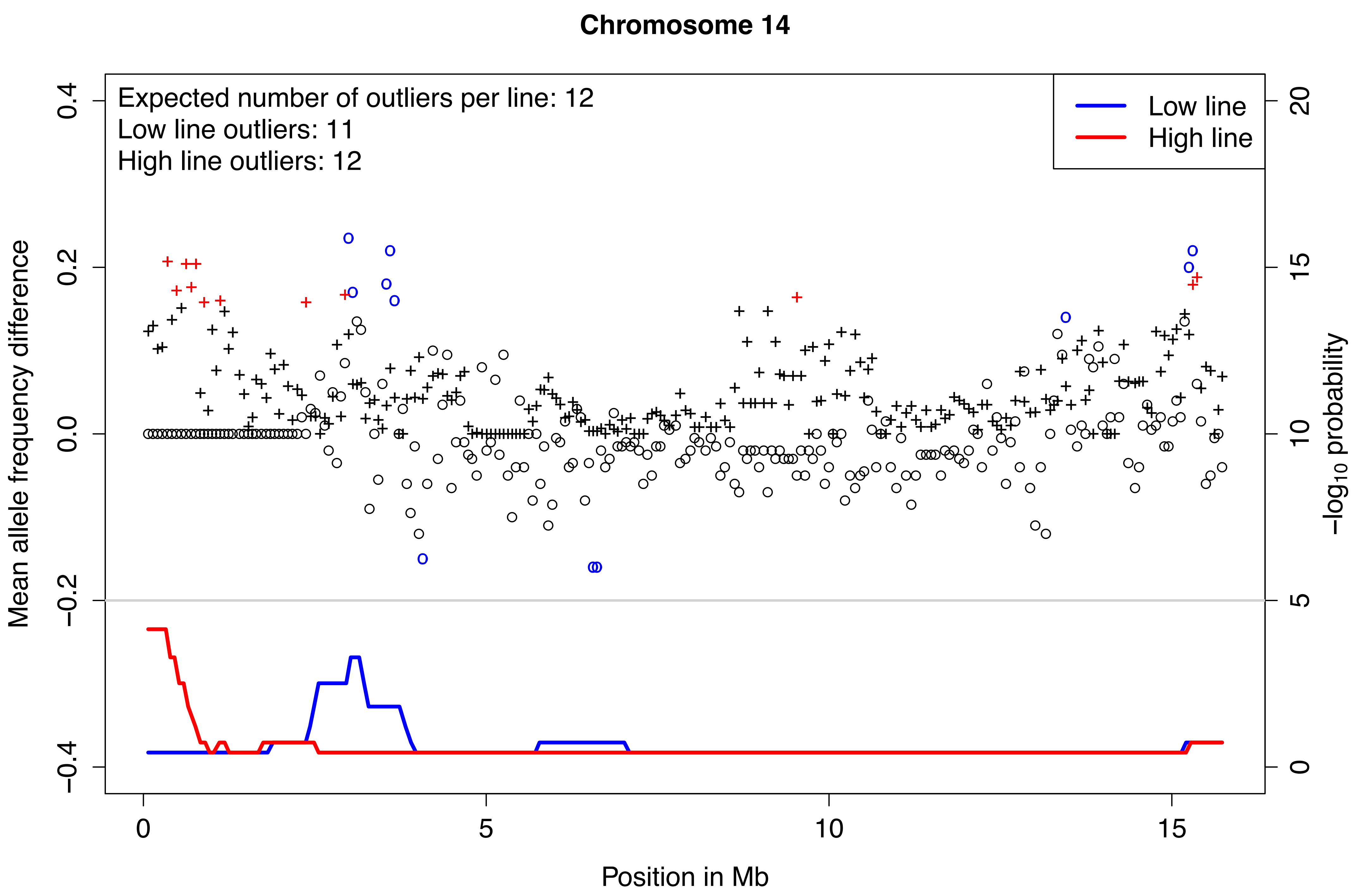
Chromosome 14
Expected number of outliers per line: 12
Low line outliers: 11
High line outliers: 12
Low line
High line
Mean allele frequency difference
−log10 probability
Position in Mb
0.4
0.2
0.0
−0.2
−0.4
20
15
10
5
0
0
5
10
15

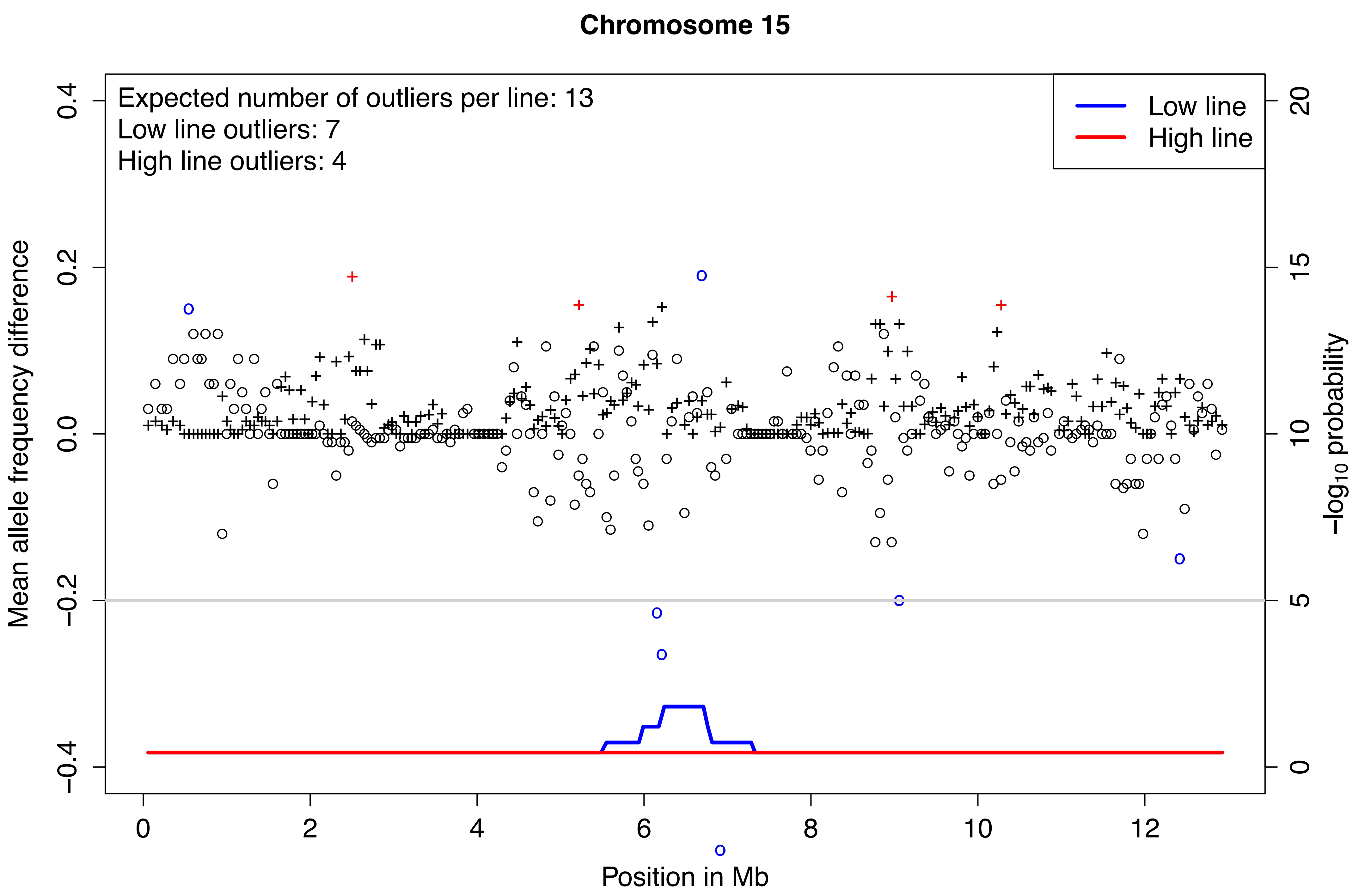

Chromosome 15
Expected number of outliers per line: 13
Low line outliers: 7
High line outliers: 4
Low line
High line
Mean allele frequency difference
−log10 probability
Position in Mb

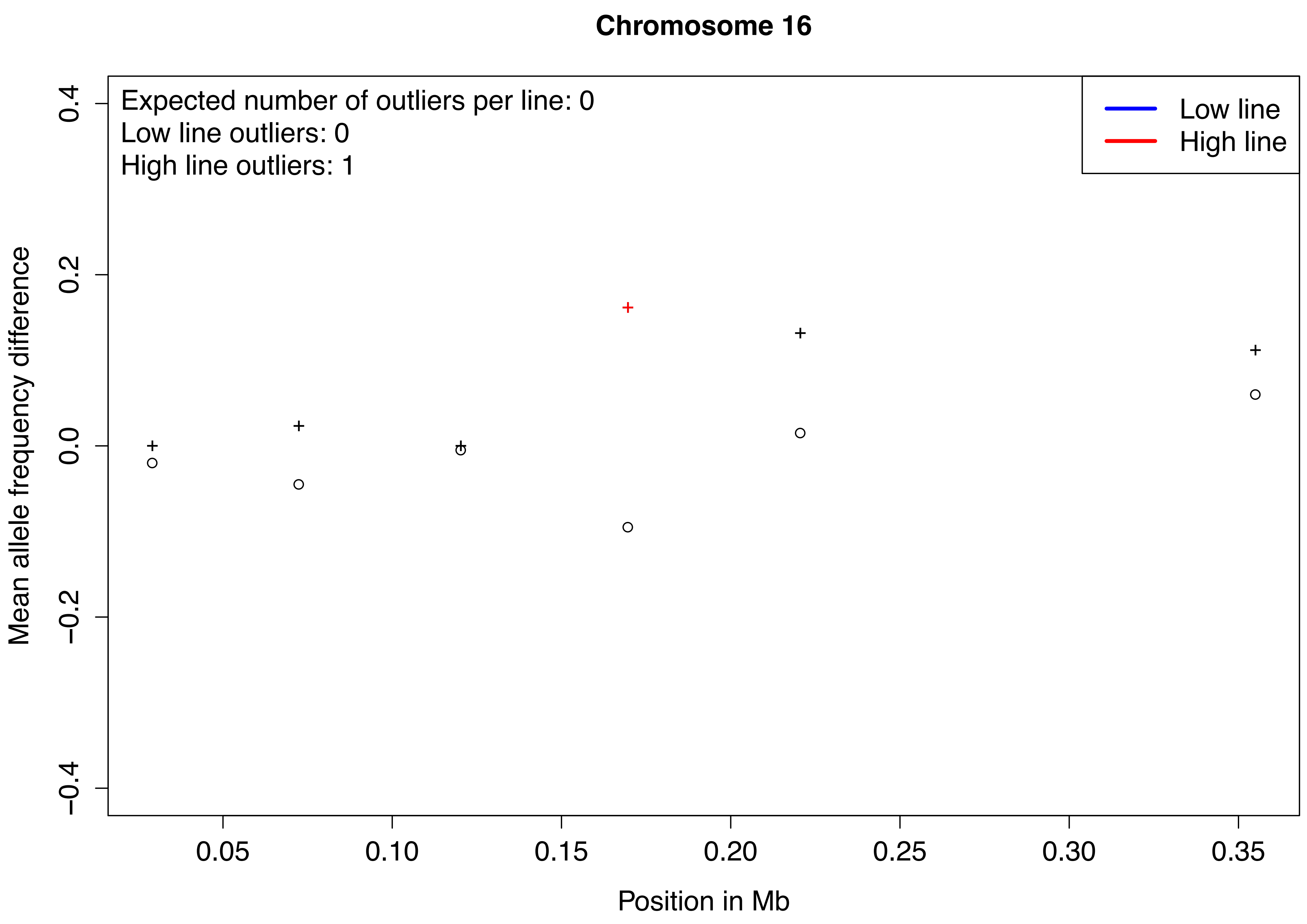

Chromosome 16
Expected number of outliers per line: 0
Low line outliers: 0
High line outliers: 1
Low line
High line
Mean allele frequency difference
0.4
0.2
0.0
−0.2
−0.4
0.05
0.10
0.15
0.20
0.25
0.30
0.35
Position in Mb

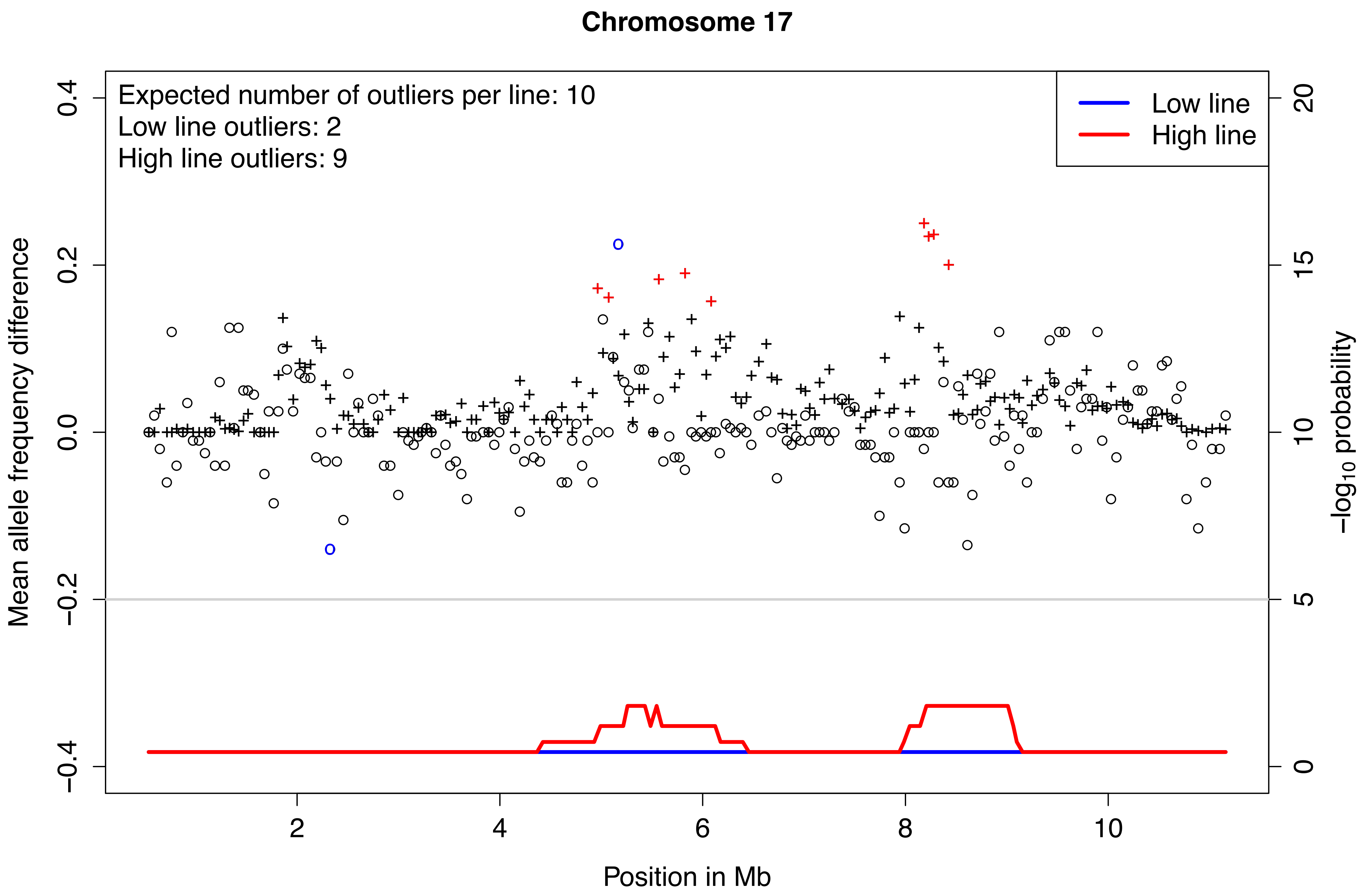

Chromosome 17
Expected number of outliers per line: 10
Low line outliers: 2
High line outliers: 9
Low line
High line
Mean allele frequency difference
−log10 probability
Position in Mb
0.4
0.2
0.0
−0.2
−0.4
20
15
10
5
0
2
4
6
8
10

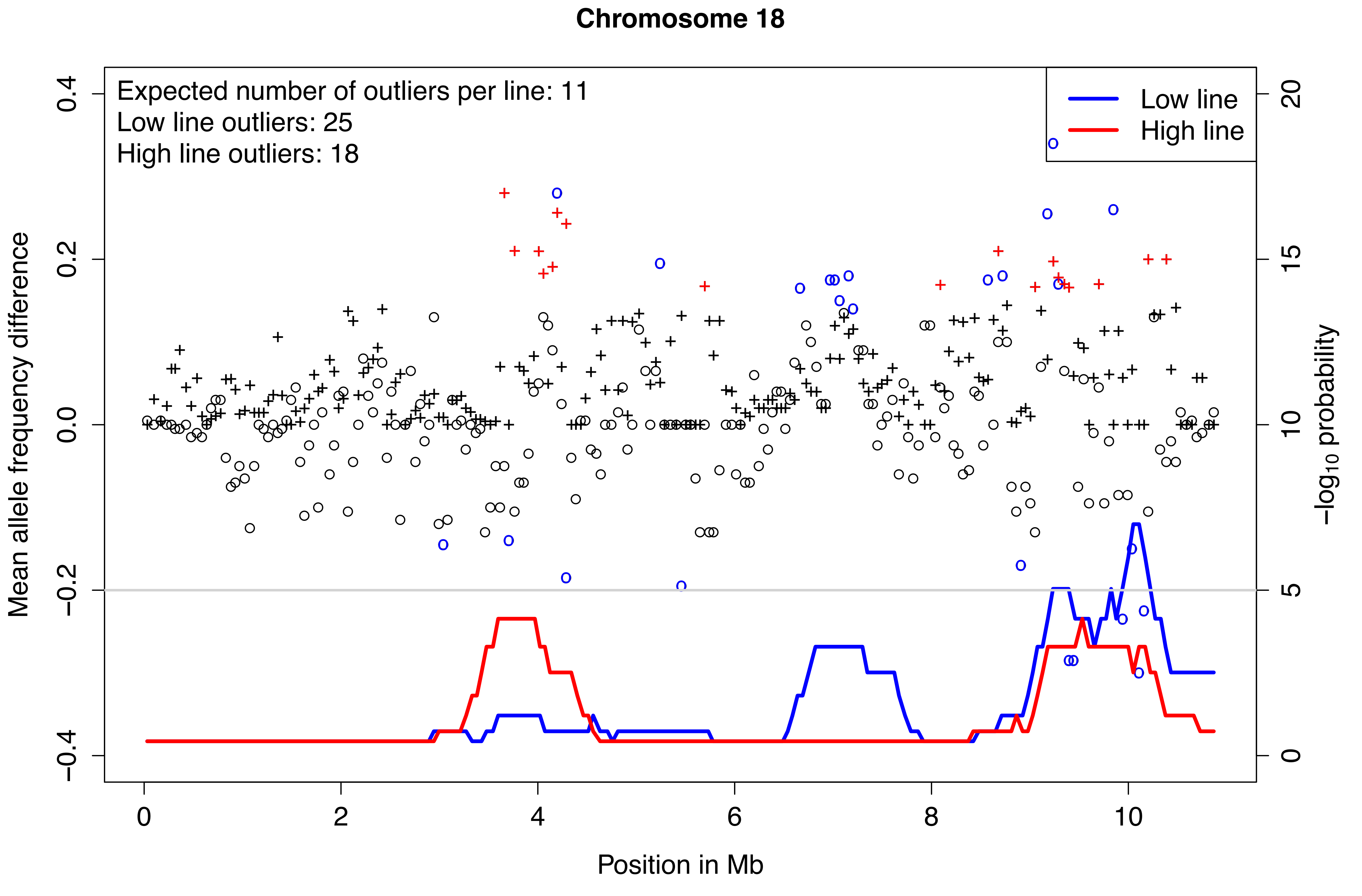

Chromosome 18
Expected number of outliers per line: 11
Low line outliers: 25
High line outliers: 18
Low line
High line
Mean allele frequency difference
−log10 probability
Position in Mb
0.4
0.2
0.0
−0.2
−0.4
20
15
10
5
0
0
2
4
6
8
10

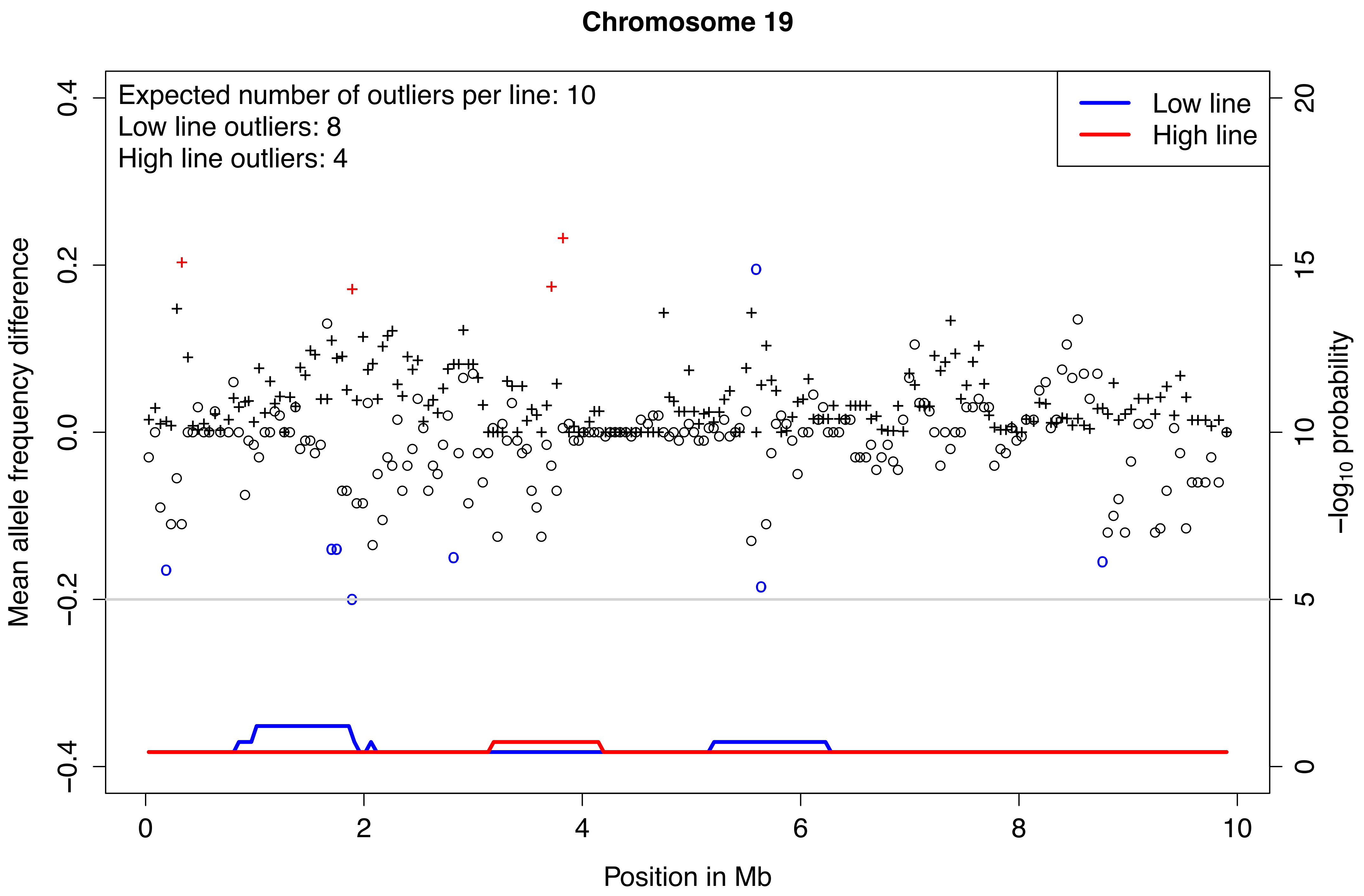
Chromosome 19
Expected number of outliers per line: 10
Low line outliers: 8
High line outliers: 4
Low line
High line
Mean allele frequency difference
−log10 probability
Position in Mb
0.4
0.2
0.0
−0.2
−0.4
20
15
10
5
0
0
2
4
6
8
10

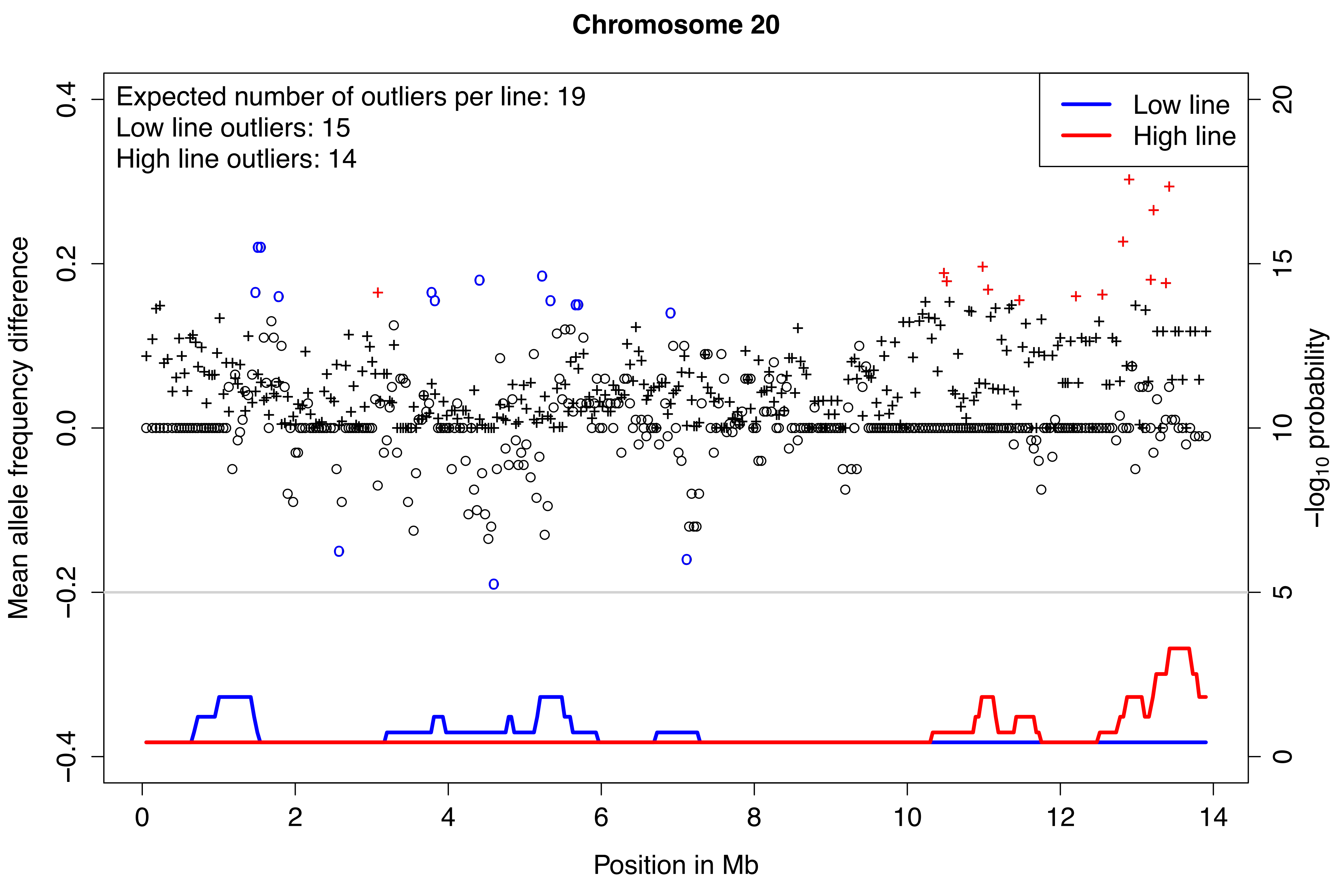

Chromosome 20
Expected number of outliers per line: 19
Low line outliers: 15
High line outliers: 14
Low line
High line
Mean allele frequency difference
−log10 probability
Position in Mb
0.4
0.2
0.0
−0.2
−0.4
20
15
10
5
0
0
2
4
6
8
10
12
14

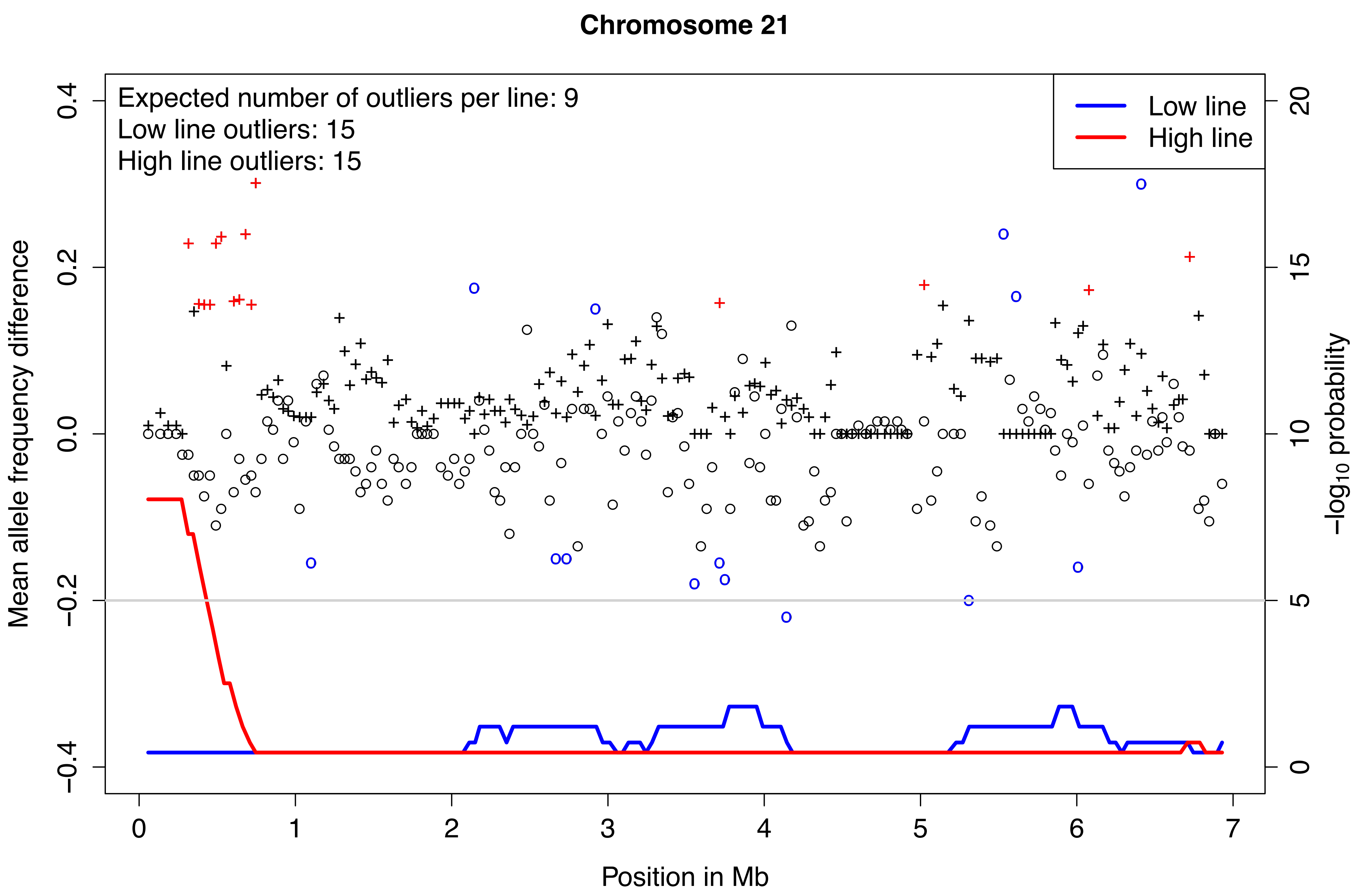
Chromosome 21
Expected number of outliers per line: 9
Low line outliers: 15
High line outliers: 15
Low line
High line
Mean allele frequency difference
−log10 probability
Position in Mb
0.4
0.2
0.0
−0.2
−0.4
20
15
10
5
0
0
1
2
3
4
5
6
7

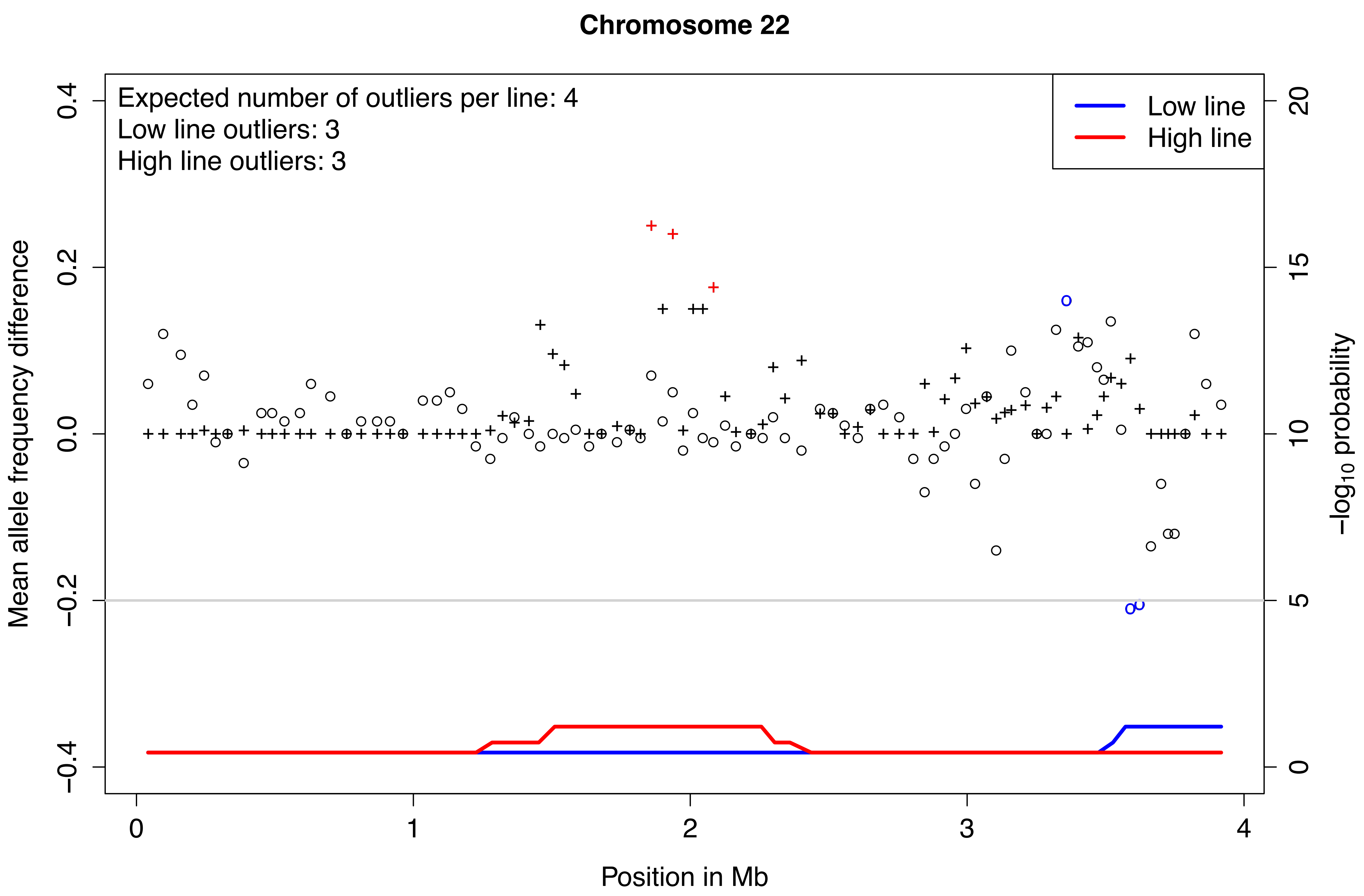

Chromosome 22
Expected number of outliers per line: 4
Low line outliers: 3
High line outliers: 3
Low line
High line
Mean allele frequency difference
−log10 probability
Position in Mb
0.4
0.2
0.0
−0.2
−0.4
20
15
10
5
0
0
1
2
3
4

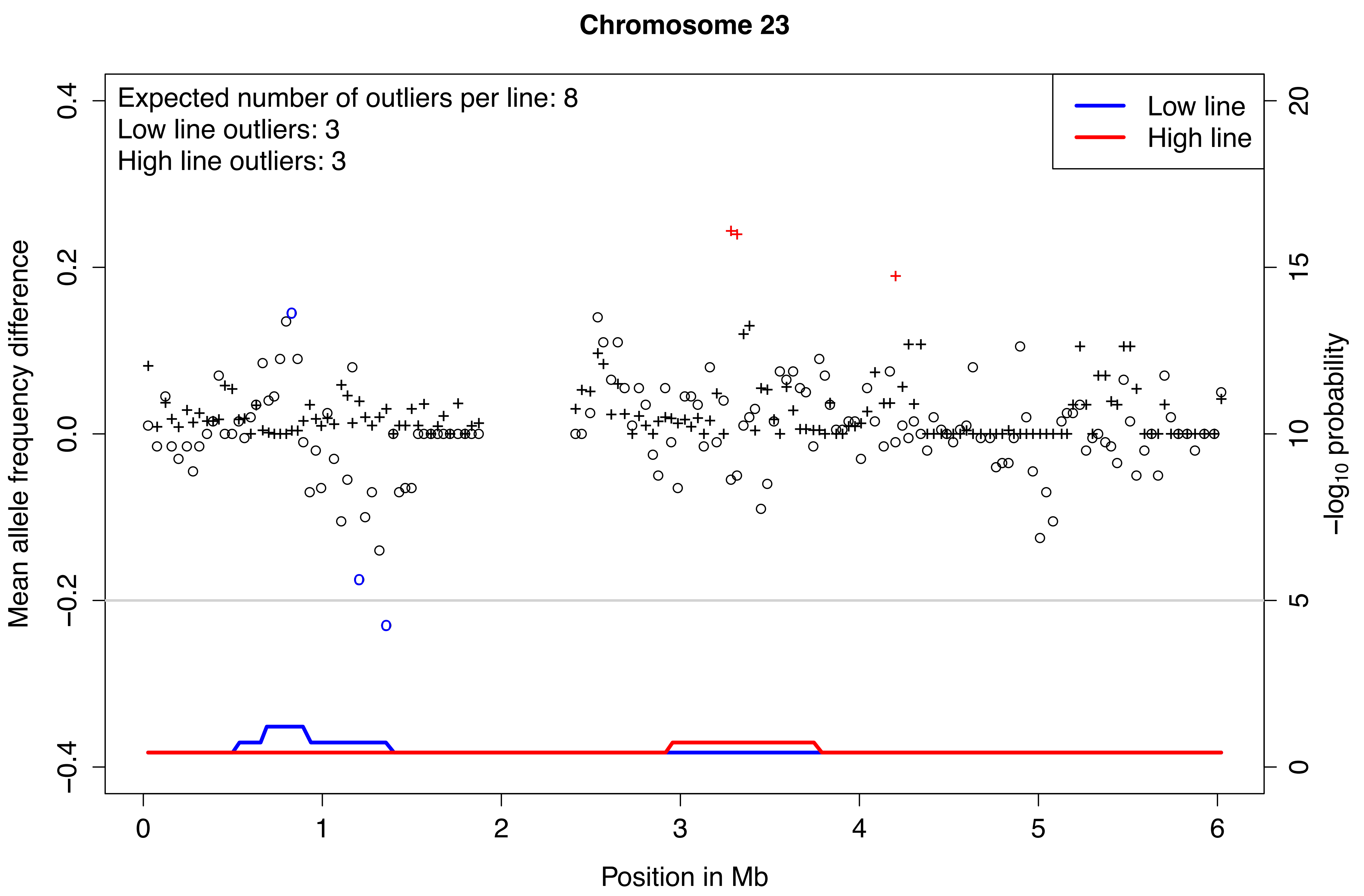

Chromosome 23
Expected number of outliers per line: 8
Low line outliers: 3
High line outliers: 3
Low line
High line
Mean allele frequency difference
−log10 probability
Position in Mb
0.4
0.2
0.0
−0.2
−0.4
20
15
10
5
0
0
1
2
3
4
5
6

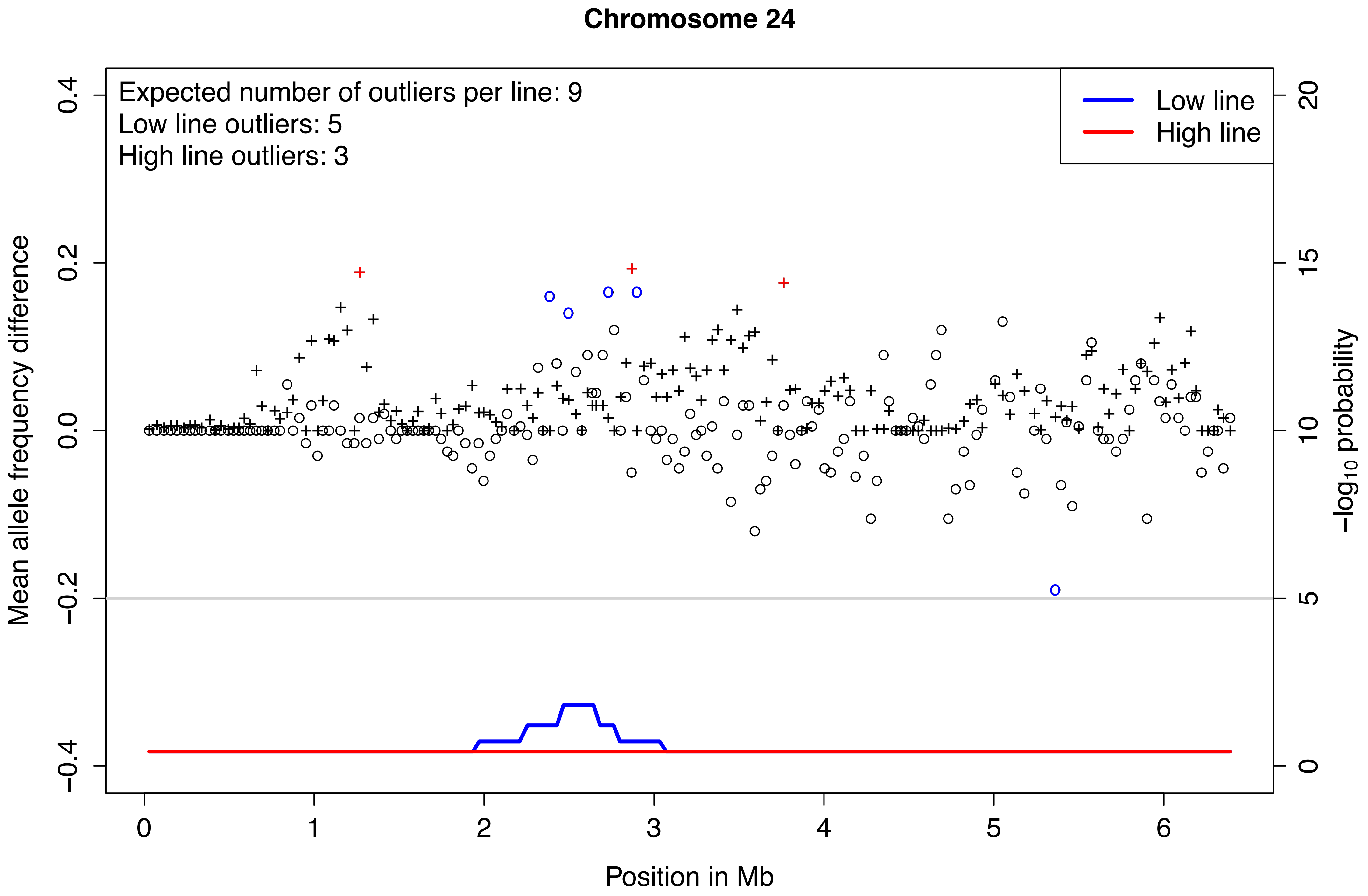

Chromosome 24
Expected number of outliers per line: 9
Low line outliers: 5
High line outliers: 3
Low line
High line
Mean allele frequency difference
−log10 probability
Position in Mb
0.4
0.2
0.0
−0.2
−0.4
20
15
10
5
0
0
1
2
3
4
5
6

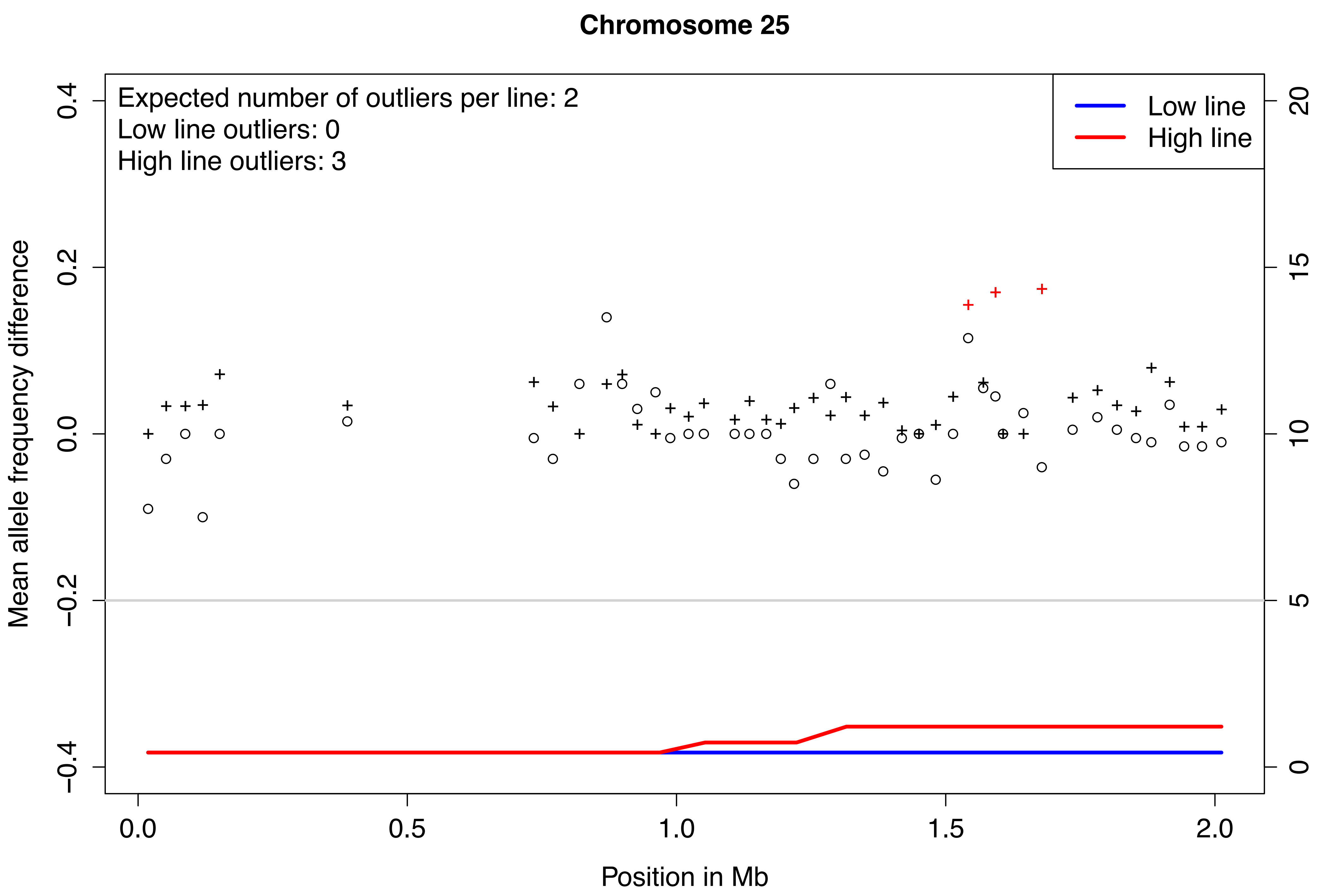
Chromosome 25
Expected number of outliers per line: 2
Low line outliers: 0
High line outliers: 3
Low line
High line
Mean allele frequency difference
Position in Mb
0.4
0.2
0.0
−0.2
−0.4
0.0
0.5
1.0
1.5
2.0
20
15
10
5
0

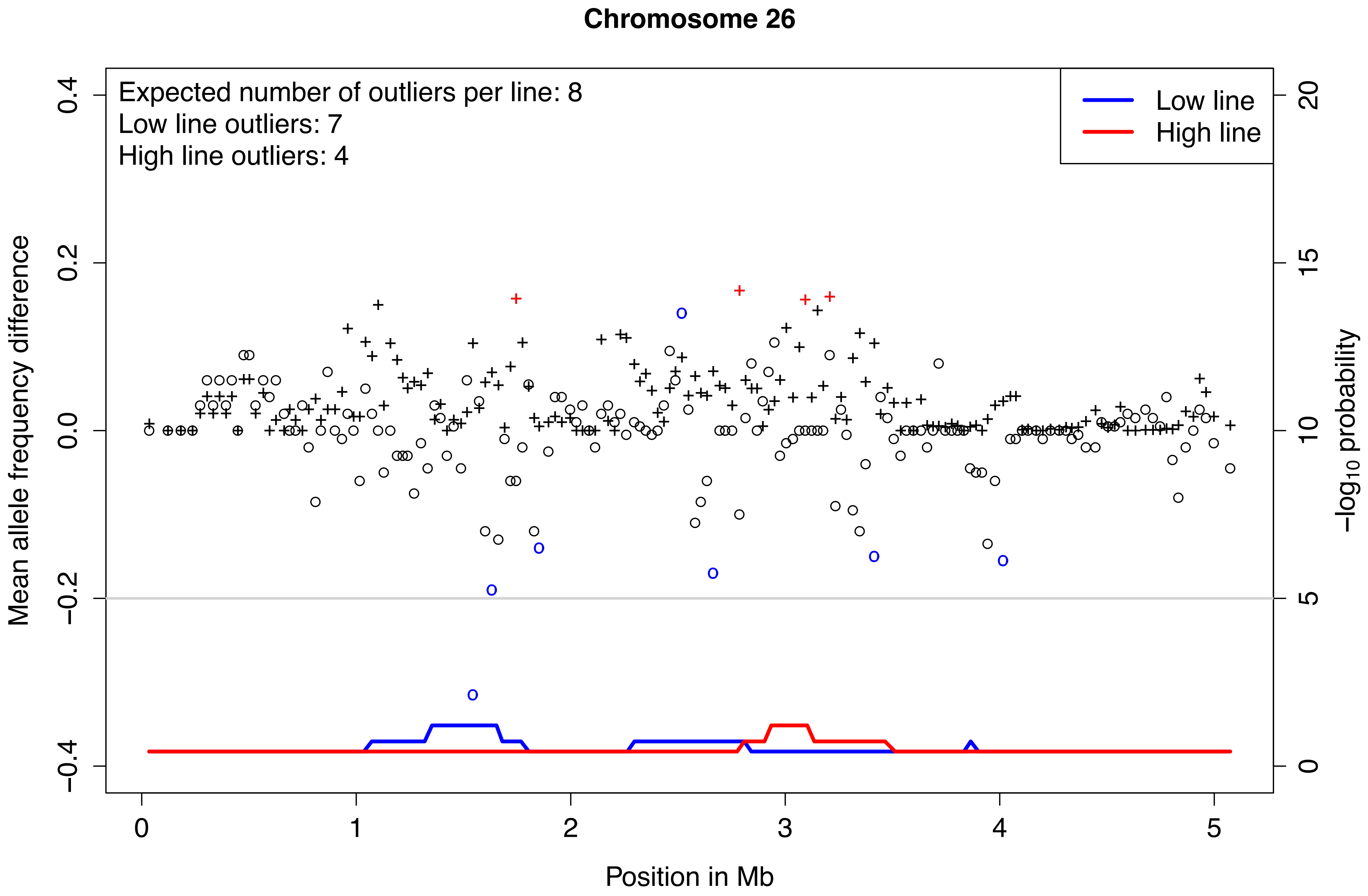
Chromosome 26
Expected number of outliers per line: 8
Low line outliers: 7
High line outliers: 4
Low line
High line
Mean allele frequency difference
−log10 probability
Position in Mb
0.4
0.2
0.0
−0.2
−0.4
20
15
10
5
0
0
1
2
3
4
5

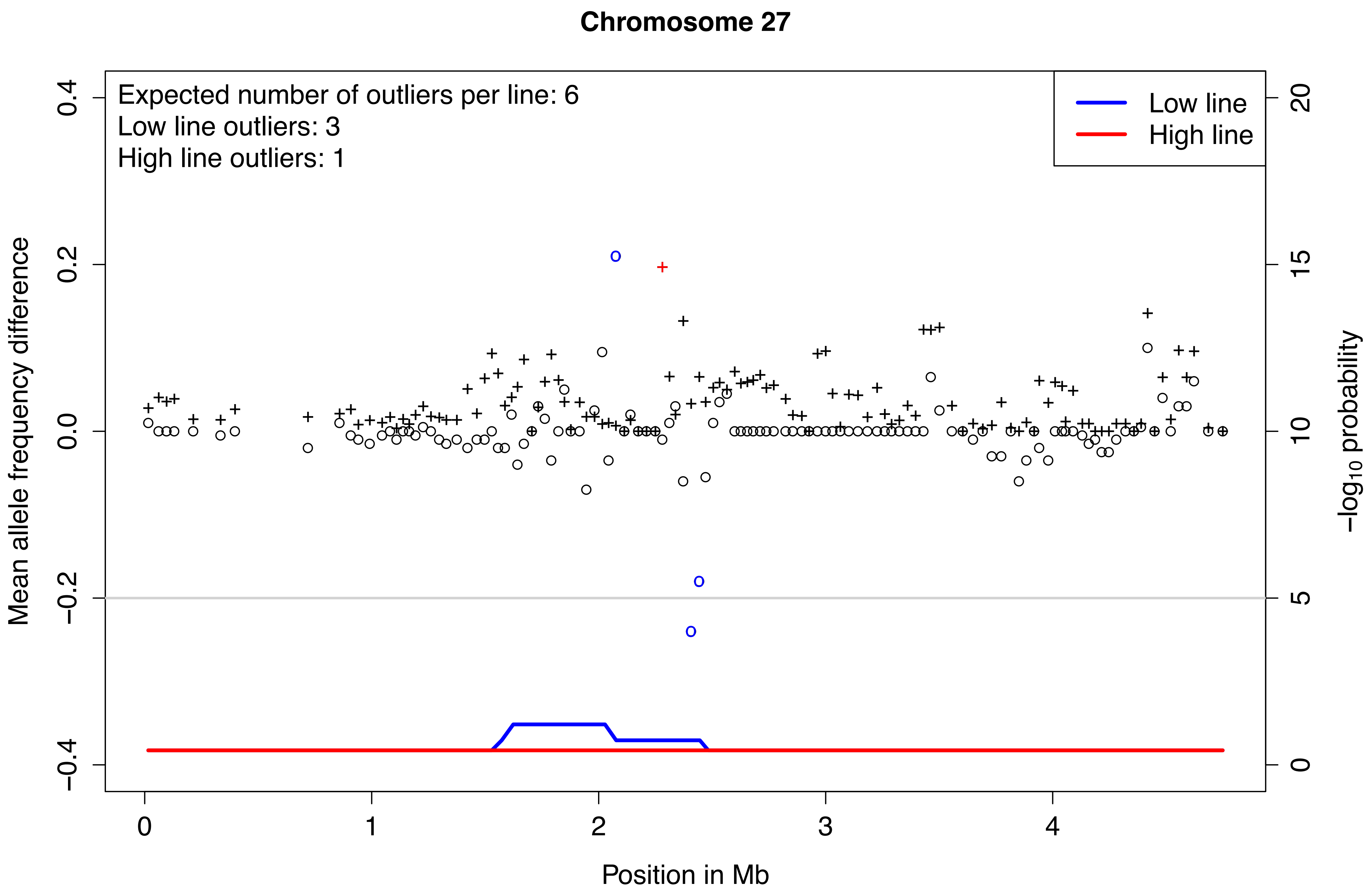
Chromosome 27
Expected number of outliers per line: 6
Low line outliers: 3
High line outliers: 1
Low line
High line
Mean allele frequency difference
−log10 probability
Position in Mb
0.4
0.2
0.0
−0.2
−0.4
20
15
10
5
0
0
1
2
3
4

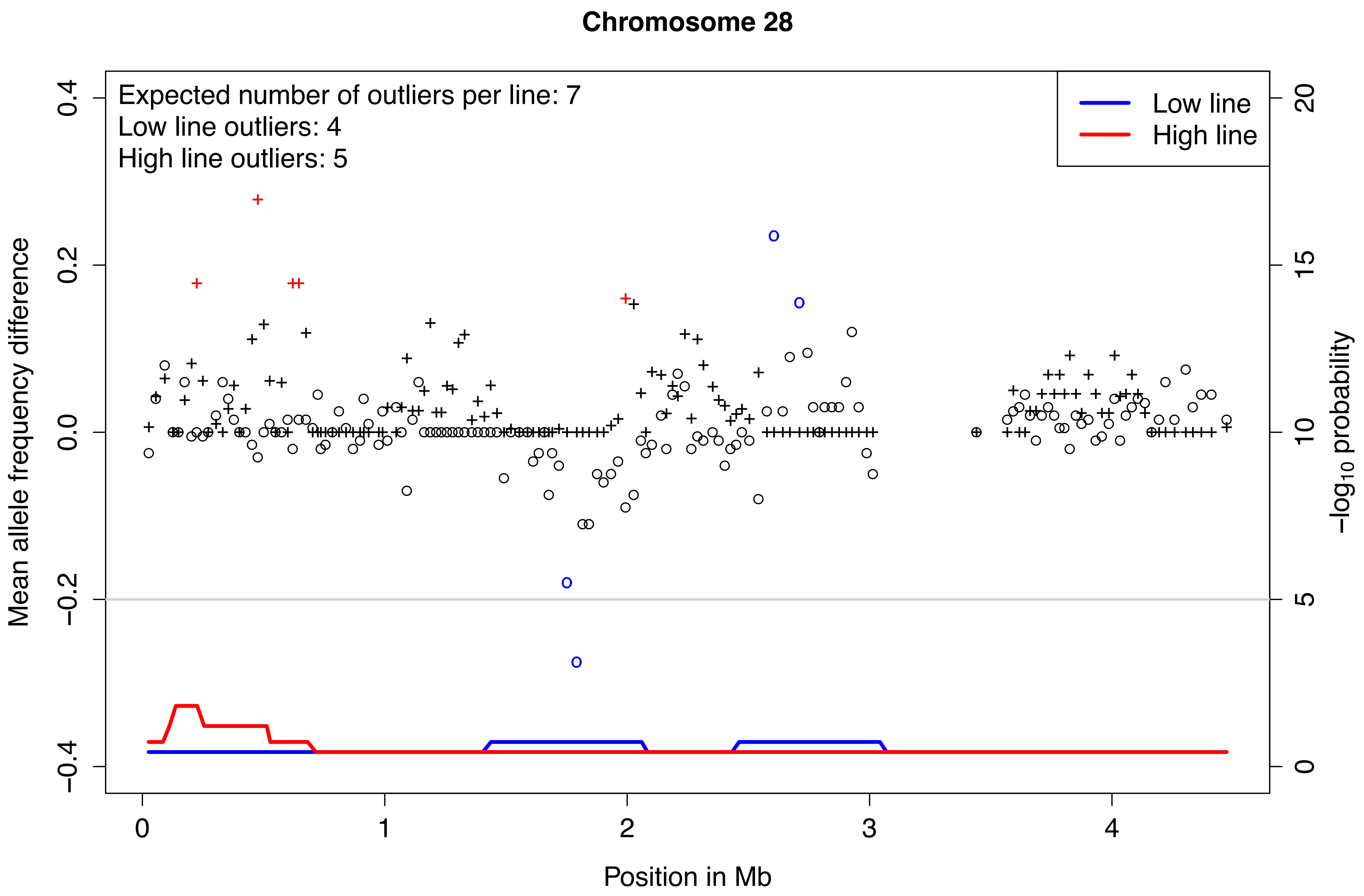

Chromosome 28
Expected number of outliers per line: 7
Low line outliers: 4
High line outliers: 5
Low line
High line
Mean allele frequency difference
−log10 probability
Position in Mb
0.4
0.2
0.0
−0.2
−0.4
20
15
10
5
0
0
1
2
3
4

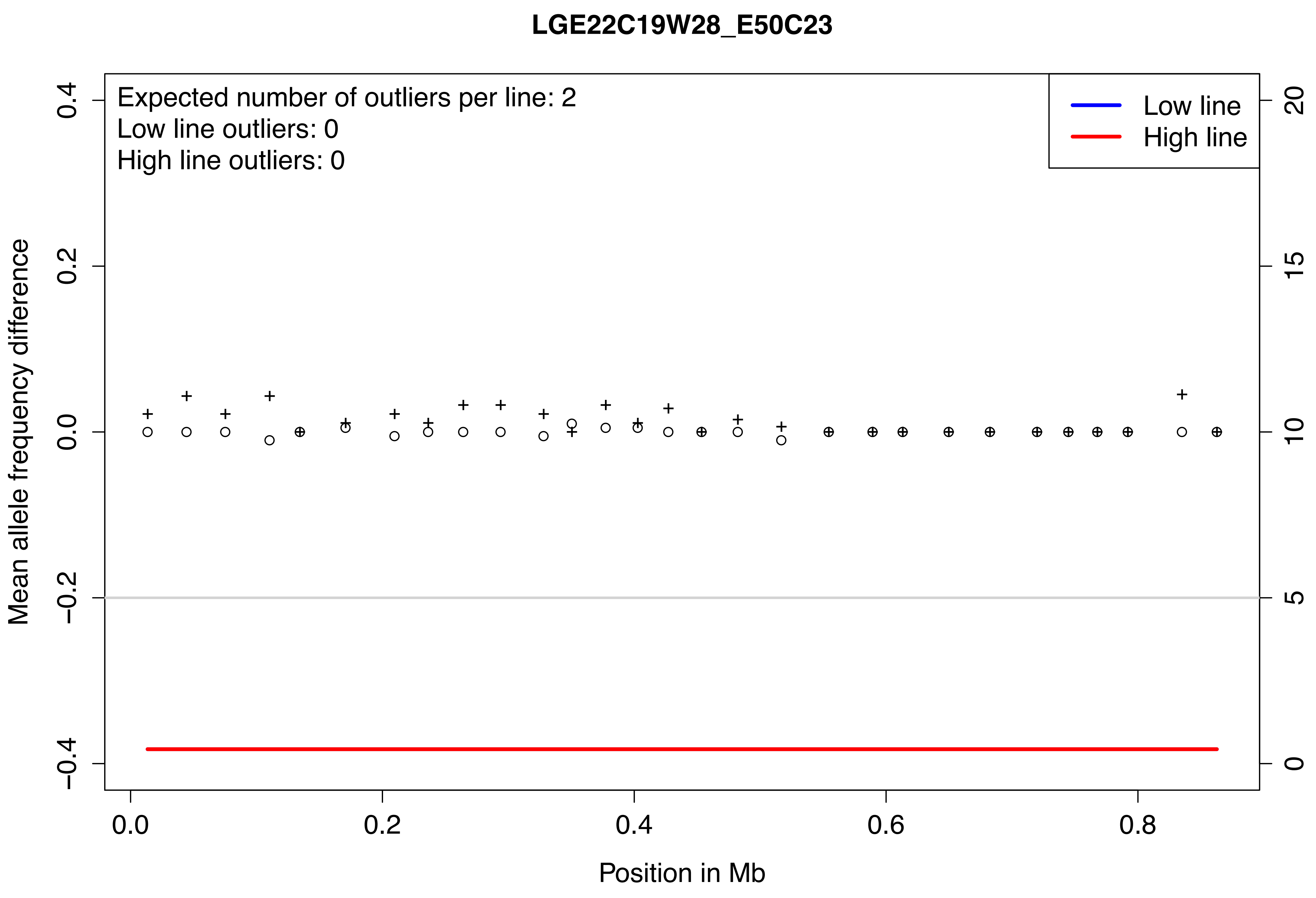
LGE22C19W28_E50C23
Expected number of outliers per line: 2
Low line outliers: 0
High line outliers: 0
Low line
High line
Mean allele frequency difference
Position in Mb
0.4
0.2
0.0
−0.2
−0.4
20
15
10
5
0
0.0
0.2
0.4
0.6
0.8

LGE64

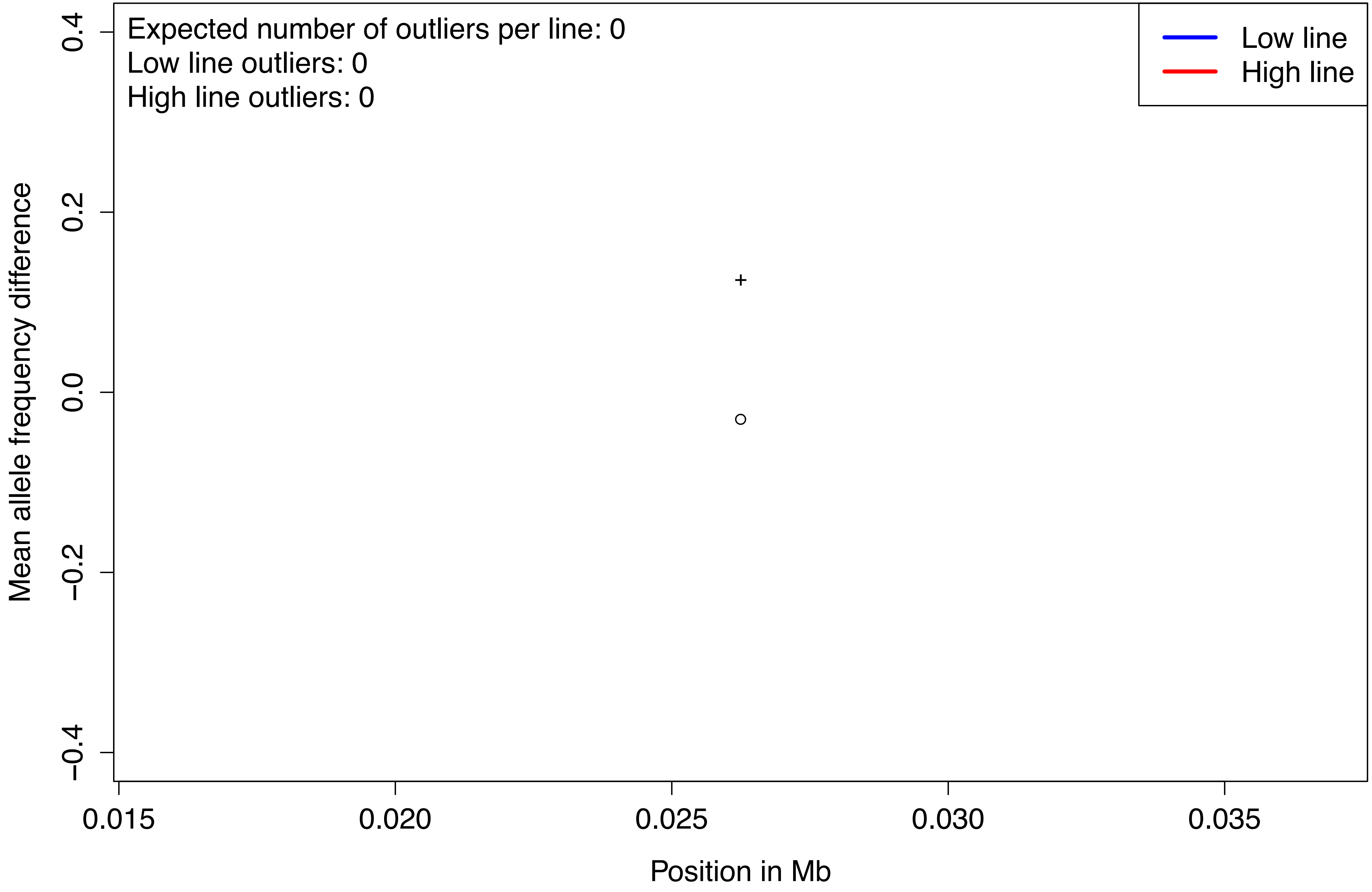
Expected number of outliers per line: 0
Low line outliers: 0
High line outliers: 0
Low line
High line
Mean allele frequency difference
0.4
0.2
0.0
−0.2
−0.4
0.015
0.020
0.025
0.030
0.035
Position in Mb

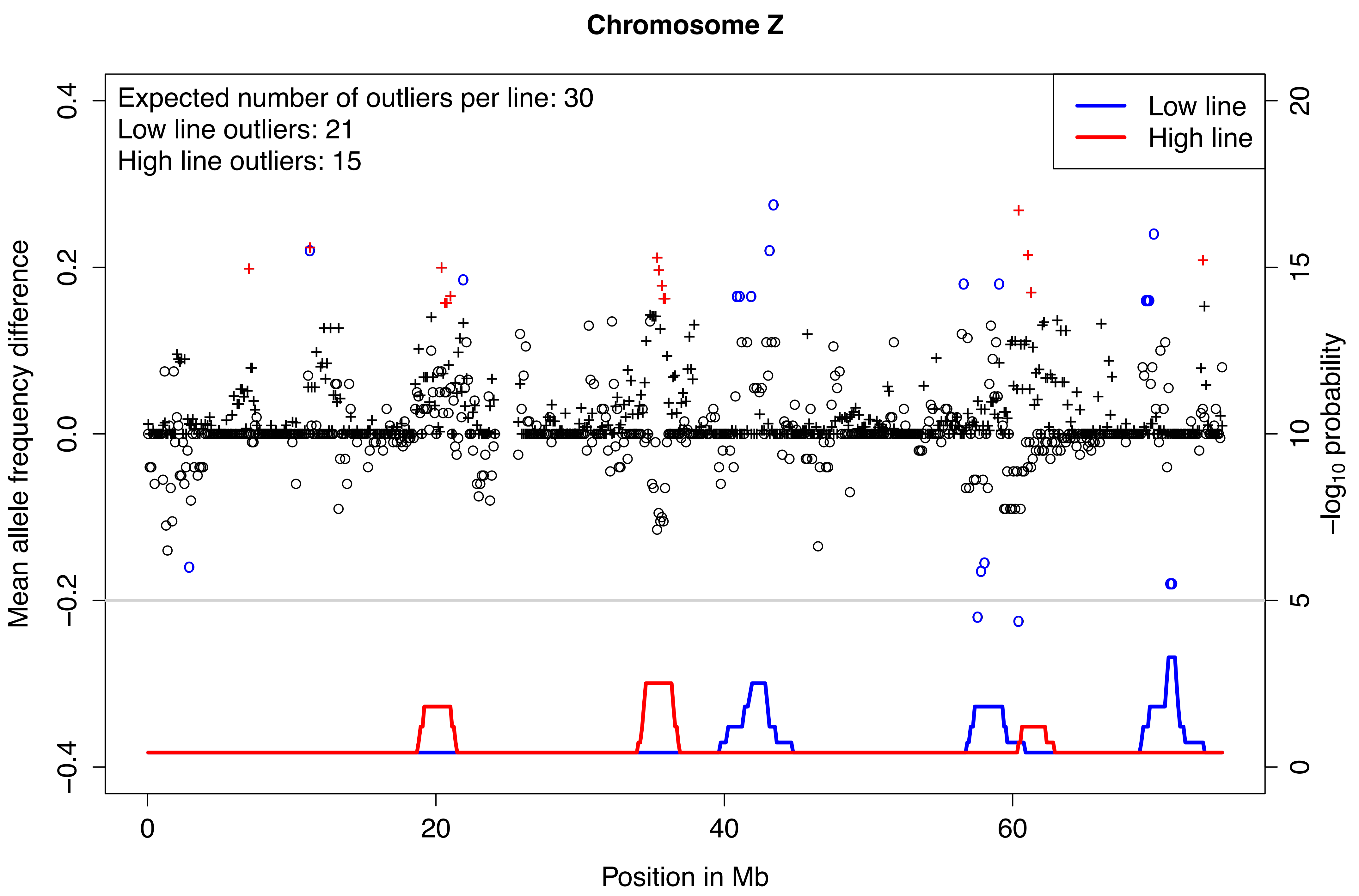

Chromosome Z
Expected number of outliers per line: 30
Low line outliers: 21
High line outliers: 15
Low line
High line
Mean allele frequency difference
−log10 probability
Position in Mb
0.4
0.2
0.0
−0.2
−0.4
20
15
10
5
0
0
20
40
60
