# Supplementary figures and images for: Genome-Wide Effects of Long-Term Divergent Selection
Source: PLoS Genet. 2010 Nov 4;6(11):e1001188. doi: 10.1371/journal.pgen.1001188 (PMC2973821; doi:10.1371/journal.pgen.1001188)

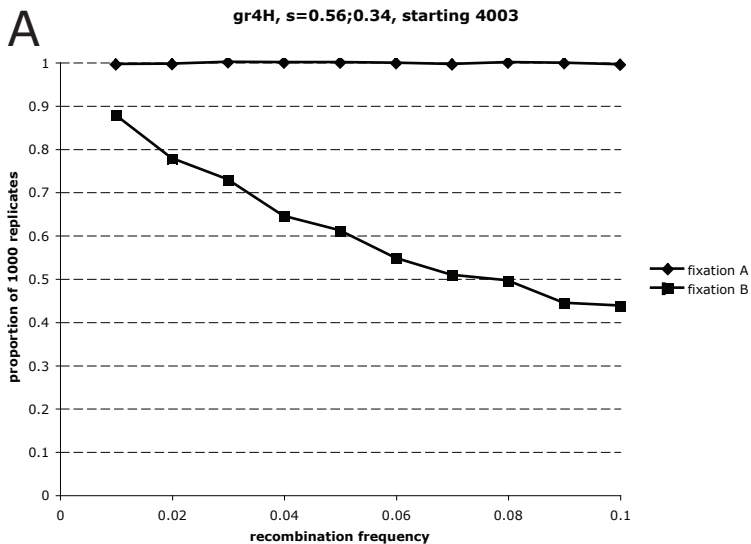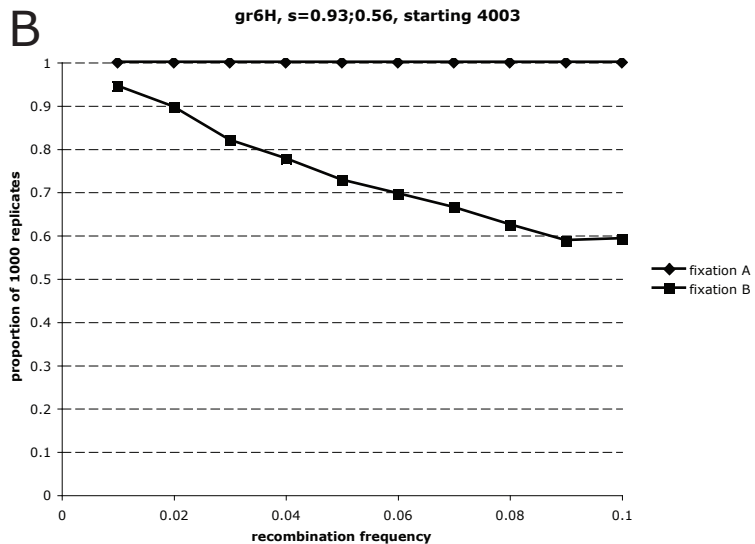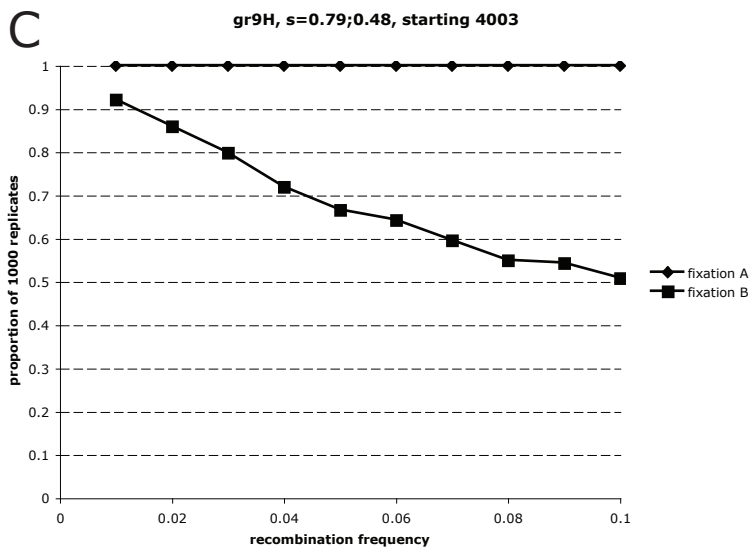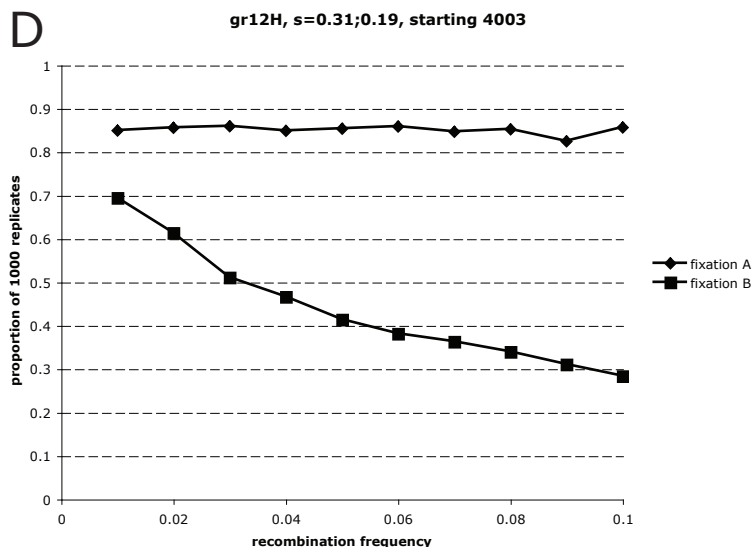

Supplement: Figure S4 — Simulations in the high line with h = 0.5 and selection coefficients from A) Growth4, B) Growth6, C) Growth9, and D) Growth12 with starting haplotype frequencies of 4003, i.e. 4/7 AB and 3/7 ab. The selection is strong enough to always lead to fixation at locus A except for Growth12 where fixation is reached in around 85% of replicates. A linked neutral locus often reaches fixation at recombination frequencies below 1–2 cM. The probability of fixation at the linked but unselected locus B is affected by the initial haplotype frequencies. A higher frequency from the beginning leads to a higher probability of fixation. (0.04 MB PDF) [file pgen.1001188.s004.pdf]

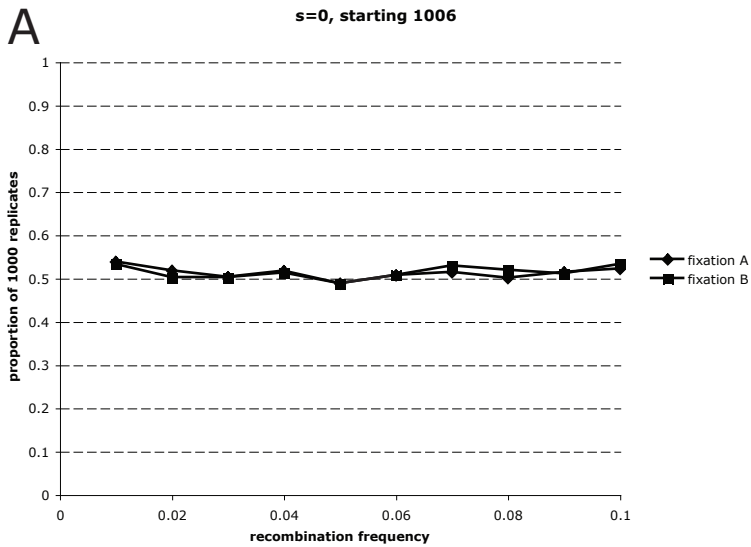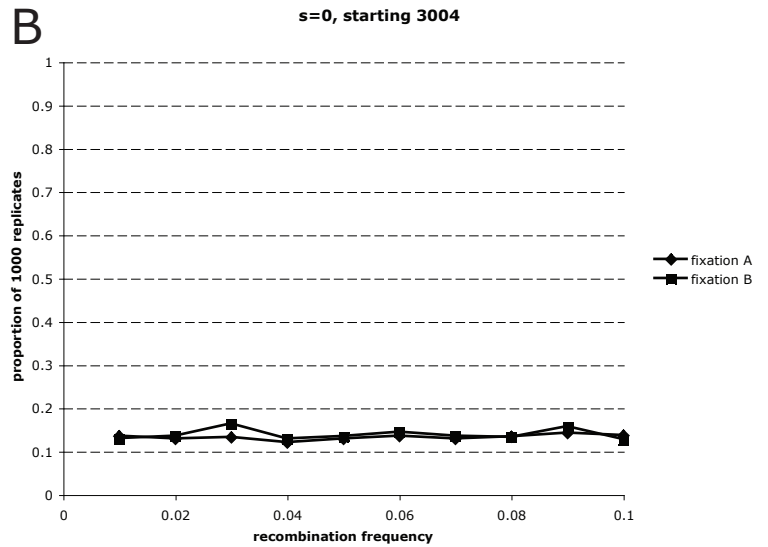

Supplement: Figure S5 — Simulations with no selection shows that fixation only occurs in 10–20% of the replicates for initial haplotype frequencies of 1006 i.e. 1/7 AB and 6/7 ab (A) and around 50% for initial haplotype frequencies of 3004 i.e. 3/7 AB and 4/7 ab (B). (0.03 MB PDF) [file pgen.1001188.s005.pdf]

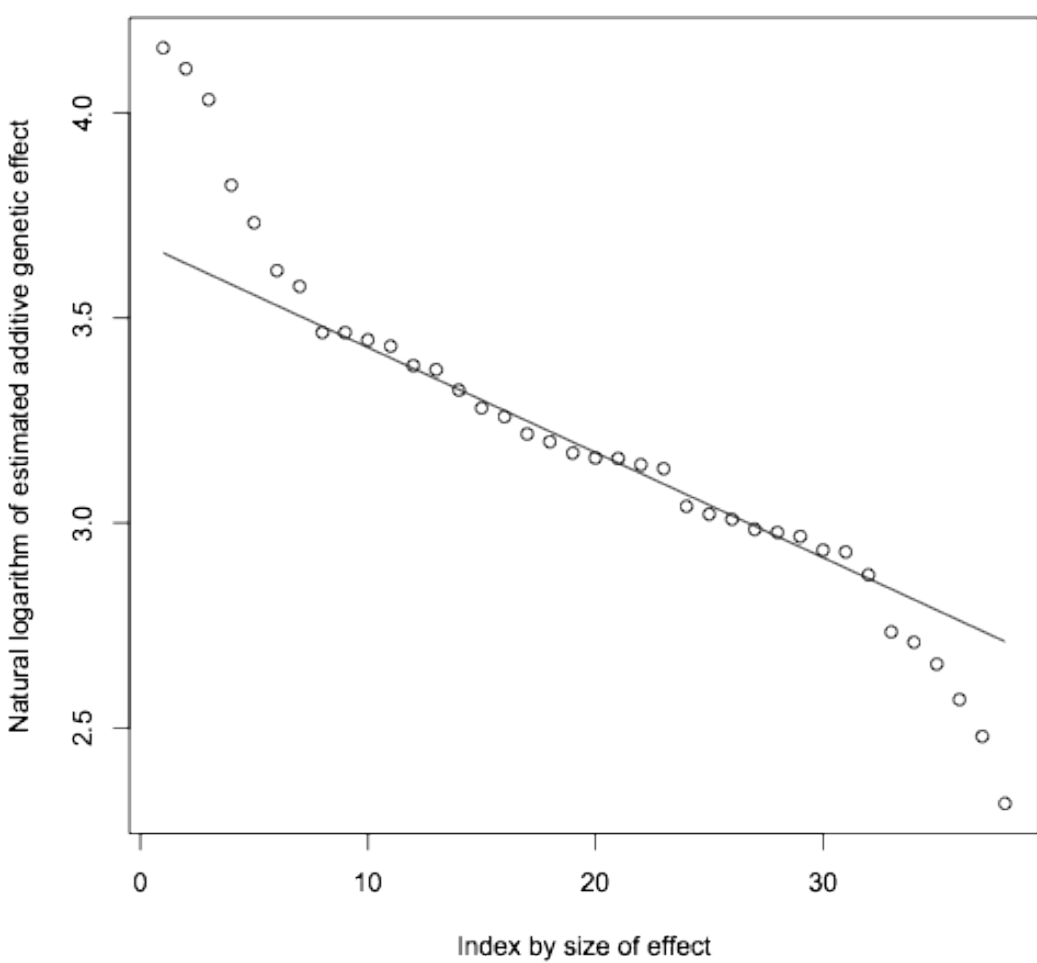

Supplement: Figure S6 — Distribution of estimated effects in the original F2 population. Distribution of the additive effect estimates in the genome scan for QTL affecting body weight at 56 days of age in an F2 intercross between the high- and low- Virginia lines. The effects are given on a natural log-scale and ordered by size. The solid line shows the linear logarithmic trend for the effects in the range 10–30. This illustrates that the relative difference between the ordered genetic effects is close to constant. At both ends of the distribution, the differences between the neighbouring effects is greater, which could indicate that these are over- and under- estimates of effects due to sampling. During selection, this distribution indicates that there will always be a smaller set of loci (often 5–10) that will have be dominant over the rest in mediating response to selection, given that the relation between the effects does not change as larger effects go to fixation. (0.03 MB PDF) [file pgen.1001188.s006.pdf]
