# Supplementary material for: Genome-Wide Effects of Long-Term Divergent Selection
Source: PLoS Genet. 2010 Nov 4;6(11):e1001188. doi: 10.1371/journal.pgen.1001188 (PMC2973821; doi:10.1371/journal.pgen.1001188)
Supplement: Table S1 — Fixation in the low and high line in different generations and sample sizes. The number of fixed alleles is dependent on the sample size, and thus results based on the total number of genotyped individuals is not directly comparable since the number of sampled individuals is not the same at the two time points. For comparison, fixation was also computed in a random sample of 10 individuals from each of the lines at both time points and the general trend is very similar regardless of the sample size. The number of SNPs fixed for different alleles has increased by 75.0% (whole data set) or 63.8% (10+10 individuals) during the ten generations. The number of SNPs fixed for the same allele has increased by 6.7% (whole data set) or 6.6% (10+10 individuals) during the ten generations. (0.03 MB PDF) [file pgen.1001188.s007.pdf]

	Earlier generation		Later generation	
Sample size (low+high)	**20+20**	**10+10**	**10+49**	**10+10**
Genotypes in both lines	56586	56573	56561	56550
Fixed for different alleles	998	1217	1746	1994
L fixed, H not fixed	8032	8126	9097	8364
H fixed, L not fixed	10237	10514	10118	10893
Fixed for same allele	23740	24201	25328	25805
