# Supplementary material for: Genome-Wide Effects of Long-Term Divergent Selection
Source: PLoS Genet. 2010 Nov 4;6(11):e1001188. doi: 10.1371/journal.pgen.1001188 (PMC2973821; doi:10.1371/journal.pgen.1001188)
Supplement: Table S2 — Fixation dynamics during 10 generations, depending on sample size. The numbers indicate the number of SNPs that are present in the two compared sets. The set for generation 40 is given before the arrow and generation 50 after the arrow. Comparison with Table S1 shows that the majority of the fixed alleles in generation 40 also are fixed in generation 50, indicating that we have large enough sample size to accurately estimate fixed alleles. Diff = fixed for different alleles in the high and low line, Same = fixed for same allele, H not L = fixed in high but not in low, L not H = fixed in low but not high. (0.03 MB PDF) [file pgen.1001188.s008.pdf]

Fixation pattern	20+20 vs 10+10	20+20 vs 10+49	10+10 vs 10+10	10+10 vs 10+49
Diff -> Diff	957	953	1090	1048
L not H -> Diff	425	225	345	181
H not L -> Diff	467	467	448	445
L not H -> L not H	6571	7164	6608	7146
H not L -> H not L	8549	8545	8734	8670
Same -> Same	23589	23584	23836	23800
L not H -> Same	937	544	866	492
H not L -> Same	1127	1125	991	984
