# Supplementary material for: Genome-Wide Effects of Long-Term Divergent Selection
Source: PLoS Genet. 2010 Nov 4;6(11):e1001188. doi: 10.1371/journal.pgen.1001188 (PMC2973821; doi:10.1371/journal.pgen.1001188)
Supplement: Table S3 — Selection coefficients for the QTLs in the body weight selected lines, calculated using i from generation 5–25 and additive effects for body weight at 56 days from [7] and [17]. (0.03 MB PDF) [file pgen.1001188.s009.pdf]

QTL	Chromosome	Low males	Low females	High males	High females
Growth1	1	0.47 (0.66)	0.27 (0.38)	0.49 (0.69)	0.30 (0.42)
Growth2	2	0.43	0.25	0.45	0.27
Growth3	2	0.54	0.31	0.56	0.34
Growth4	3	0.54	0.31	0.56	0.34
Growth5	3	0.72	0.42	0.75	0.46
Growth6	4	0.89 (0.70)	0.51 (0.40)	0.93 (0.73)	0.56 (0.44)
Growth7	4	0.64	0.37	0.67	0.40
Growth8	5	0.42	0.24	0.44	0.27
Growth9	7	0.76 (0.83)	0.44 (0.48)	0.79 (0.87)	0.48 (0.53)
Growth10	13	0.41	0.24	0.43	0.26
Growth12	20	0.29	0.17	0.31	0.19
