# Supplementary material for: Genome-Wide Effects of Long-Term Divergent Selection
Source: PLoS Genet. 2010 Nov 4;6(11):e1001188. doi: 10.1371/journal.pgen.1001188 (PMC2973821; doi:10.1371/journal.pgen.1001188)
Supplement: Table S4 — Number of generations until fixation for different QTLs. All simulations have starting frequencies 4/7 AB and 3/7 ab. A single additive QTL is assumed. H denotes the high line and L the low line. (0.04 MB PDF) [file pgen.1001188.s010.pdf]

QTL	s males	s females	50% fixed	90% fixed	95% fixed	100% fixed
Growth12 H	0.31	0.19	27	-	-	-
Growth4 H	0.56	0.34	17	26	28	-
Growth9 H	0.79	0.48	12	18	20	32
Growth6 H	0.93	0.56	11	15	17	28
Growth12 L	0.29	0.17	28	-	-	-
Growth4 L	0.54	0.31	17	27	31	-
Growth9 L	0.76	0.44	12	19	22	39
Growth6 L	0.89	0.51	11	16	18	29
