# Supplementary material for: Genome-Wide Effects of Long-Term Divergent Selection
Source: PLoS Genet. 2010 Nov 4;6(11):e1001188. doi: 10.1371/journal.pgen.1001188 (PMC2973821; doi:10.1371/journal.pgen.1001188)
Supplement: Table S5 — Number of generations until fixation, for different starting frequencies at Growth9 in the high line, assuming additive QTL effects. Our notation for the starting allele frequencies of two loci A and B with alleles A/a and B/b is a four digit code xyzw, where x is the proportion of haplotype AB, y is the proportion of haplotype Ab, z is the proportion of haplotype aB and w is the proportion of haplotype ab. (0.02 MB PDF) [file pgen.1001188.s011.pdf]

Starting frequency	Generation with 50% fixed	Generation with 90% fixed	Generation with 95% fixed	Generation with 100% fixed
1006	17	22	24	32
3004	13	20	21	37
4003	12	18	20	32
6001	8	14	16	25
