# Supplementary material for: Genome-Wide Effects of Long-Term Divergent Selection
Source: PLoS Genet. 2010 Nov 4;6(11):e1001188. doi: 10.1371/journal.pgen.1001188 (PMC2973821; doi:10.1371/journal.pgen.1001188)
Supplement: Table S6 — Population parameters for the body weight selected lines, p is proportion selected, i is selection intensity calculated from p. p was calculated separately for males and females by dividing the number of selected by the average number of individuals in each generation (n = 268 in the high line and n = 309 in the low line, and assuming equal sex ratio in the offspring). The selection intensities, i, were retrieved from p using the tables on pp 379–380 in Falconer and Mackay [18]. Since the number of males and females selected in each generation are not equal, i is different for males and females leading to different s for males and females. The effective population size for each of the three generation intervals was estimated as 4*Nm*Nf/(Nm+Nf). The effective population size for generations 1–40 estimated as the harmonic mean is 40/((4/27.43)+(26/38.40)+(15/44.80)) = 34.55, whereas until generation 50 it is 40/((4/27.43)+(26/38.4)+(25/44.80)) = 36.21. (0.03 MB PDF) [file pgen.1001188.s012.pdf]

| | **Generation 1-4** | **Generation 5-25** | **Generation 26-50** |
|---|---|---|---|
| Selected males ($N_m$) | 8 | 12 | 14 |
| Selected females ($N_f$) | 48 | 48 | 56 |
| Effective population size | 27.43 | 38.40 | 44.80 |
| Low line males *p*, (*i*) | 0.059 (1.985) | 0.089 (1.804) | 0.10 (1.755) |
| Low line Females *p*, (*i*) | 0.36 (1.039) | 0.36 (1.039) | 0.41 (0.948) |
| High line males *p*, (*i*) | 0.052 (2.063) | 0.077 (1.887) | 0.090 (1.804) |
| High line females *p*, (*i*) | 0.31 (1.138) | 0.31 (1.138) | 0.36 (1.039) |
