# Supplementary material for: Genome-Wide Effects of Long-Term Divergent Selection
Source: PLoS Genet. 2010 Nov 4;6(11):e1001188. doi: 10.1371/journal.pgen.1001188 (PMC2973821; doi:10.1371/journal.pgen.1001188)
Supplement: Table S7 — Clusters as defined by different maximum distance between, and minimum number of, SNPs. (0.03 MB PDF) [file pgen.1001188.s013.pdf]

Maximum distance	Minimum SNPs	Number of clusters	Number of markers in clusters	Proportion of genome in clusters
0.5 Mb	2	131	936 (93.8%)	7.5%
0.5 Mb	3	98	870 (87.2%)	7.2%
0.5 Mb	4	77	807 (80.7%)	6.6%
0.5 Mb	5	66	763 (76.5%)	6.3%
1 Mb	2	116	959 (96.1%)	10.2%
1 Mb	3	90	907 (90.9%)	9.6%
1 Mb	4	76	865 (86.7%)	9.3%
1 Mb	5	65	821 (82.3%)	8.6%
2 Mb	2	93	963 (96.5%)	14.1%
2 Mb	3	78	933 (93.5%)	13.8%
2 Mb	4	70	909 (91.1%)	13.6%
2 Mb	5	61	873 (87.5%)	12.9%
