# Supplementary material for: Genome-Wide Effects of Long-Term Divergent Selection
Source: PLoS Genet. 2010 Nov 4;6(11):e1001188. doi: 10.1371/journal.pgen.1001188 (PMC2973821; doi:10.1371/journal.pgen.1001188)
Supplement: Table S8 — Number of clusters with at least 5 SNPs fixed for different alleles in the two lines in generation 40 and 50, together with their length distribution. (0.02 MB PDF) [file pgen.1001188.s014.pdf]

	Total	≥1 Mb	≥2 Mb	≥3 Mb
40 gen, all chromosomes	65	33	15	8
40 gen, macro chromosomes	33	21	11	6
40 gen, Z chromosome	6	2	1	1
40 gen, smaller chromosomes	26	10	3	1
50 gen, all chromosomes	102	58	23	12
50 gen, macro chromosomes	51	30	13	8
50 gen, Z chromosome	7	4	2	2
50 gen, other chromosomes	44	24	8	2
