# Supplementary material for: Genome-Wide Effects of Long-Term Divergent Selection
Source: PLoS Genet. 2010 Nov 4;6(11):e1001188. doi: 10.1371/journal.pgen.1001188 (PMC2973821; doi:10.1371/journal.pgen.1001188)
Supplement: Table S9 — Number of clusters with at least 2 SNPs fixed for different alleles in the two lines in generation 40 and 50, together with their length distribution. (0.02 MB PDF) [file pgen.1001188.s015.pdf]

	**Total**	**≥1 Mb**	**≥2 Mb**	**≥3 Mb**
40 gen, all chromosomes	116	35	15	8
40 gen, macro chromosomes	59	23	11	6
40 gen, Z chromosome	8	2	1	1
40 gen, smaller chromosomes	49	10	3	1
50 gen, all chromosomes	163	60	23	12
50 gen, macro chromosomes	84	31	13	8
50 gen, Z chromosome	11	4	2	2
50 gen, other chromosomes	68	25	8	2
